# Recognition and management of community-acquired acute kidney injury in low-resource settings in the ISN 0by25 trial: A multi-country feasibility study

**DOI:** 10.1371/journal.pmed.1003408

**Published:** 2021-01-14

**Authors:** Etienne Macedo, Ulla Hemmila, Sanjib Kumar Sharma, Rolando Claure-Del Granado, Henry Mzinganjira, Emmanuel A. Burdmann, Jorge Cerdá, John Feehally, Fredric Finkelstein, Guillermo García-García, Vivekanand Jha, Norbert H. Lameire, Euyhyun Lee, Nathan W. Levin, Andrew Lewington, Raúl Lombardi, Michael V. Rocco, Eliah Aronoff-Spencer, Marcello Tonelli, Karen Yeates, Giuseppe Remuzzi, Ravindra L. Mehta, for the ISN 0by25 Trial Study Group

**Affiliations:** 1 Division of Nephrology, Department of Medicine, University of California San Diego, San Diego, California, United States of America; 2 College of Medicine, University of Malawi, Blantyre, Malawi; 3 Department of Internal Medicine, B.P. Koirala Institute of Health Sciences, Dharan, Nepal; 4 Division of Nephrology, Hospital Obrero #2–Caja Nacional de Salud, School of Medicine, Universidad Mayor de San Simón, Cochabamba, Bolivia; 5 LIM 12, Division of Nephrology, University of São Paulo Medical School, São Paulo, Brazil; 6 Division of Nephrology, Department of Medicine, Albany Medical College, Albany, New York, United States of America; 7 University of Leicester, Leicester, United Kingdom; 8 Yale University, New Haven, Connecticut, United States of America; 9 Hospital Civil de Guadalajara, University of Guadalajara Health Science Center, Guadalajara, Jalisco, Mexico; 10 George Institute for Global Health, University of New South Wales, New Delhi, India; 11 School of Public Health, Imperial College London, London, United Kingdom; 12 Manipal Academy of Higher Education, Manipal, India; 13 Nephrology Section, Department of Internal Medicine, University Hospital, Ghent, Belgium; 14 Altman Clinical and Translational Research Institute, University of California San Diego, La Jolla, California, United States of America; 15 Mount Sinai School of Medicine, Renal Research Institute, New York, New York, United States of America; 16 Department of Nephrology, Leeds Teaching Hospitals NHS Trust, Leeds, United Kingdom; 17 NIHR Leeds In Vitro Diagnostics Co-operative, Leeds, United Kingdom; 18 Department of Critical Care Medicine, Servicio Médico Integral, Montevideo, Uruguay; 19 Section of Nephrology, Department of Internal Medicine, Wake Forest School of Medicine, Winston-Salem, North Carolina, United States of America; 20 University of Calgary, Calgary, Alberta, Canada; 21 Division of Nephrology, Department of Medicine, Queen’s University, Kingston, Ontario, Canada; 22 Istituto di Ricerche Farmacologiche Mario Negri, Istituto di Ricovero e Cura a Carattere Scientifico, Bergamo, Italy; Royal Derby Hospital, UNITED KINGDOM

## Abstract

**Background:**

Acute kidney injury (AKI) is increasingly encountered in community settings and contributes to morbidity, mortality, and increased resource utilization worldwide. In low-resource settings, lack of awareness of and limited access to diagnostic and therapeutic interventions likely influence patient management. We evaluated the feasibility of the use of point-of-care (POC) serum creatinine and urine dipstick testing with an education and training program to optimize the identification and management of AKI in the community in 3 low-resource countries.

**Methods and findings:**

Patients presenting to healthcare centers (HCCs) from 1 October 2016 to 29 September 2017 in the cities Cochabamba, Bolivia; Dharan, Nepal; and Blantyre, Malawi, were assessed utilizing a symptom-based risk score to identify patients at moderate to high AKI risk. POC testing for serum creatinine and urine dipstick at enrollment were utilized to classify these patients as having chronic kidney disease (CKD), acute kidney disease (AKD), or no kidney disease (NKD). Patients were followed for a maximum of 6 months with repeat POC testing. AKI development was assessed at 7 days, kidney recovery at 1 month, and progression to CKD and mortality at 3 and 6 months. Following an observation phase to establish baseline data, care providers and physicians in the HCCs were trained with a standardized protocol utilizing POC tests to evaluate and manage patients, guided by physicians in referral hospitals connected via mobile digital technology. We evaluated 3,577 patients, and 2,101 were enrolled: 978 in the observation phase and 1,123 in the intervention phase. Due to the high number of patients attending the centers daily, it was not feasible to screen all patients to assess the actual incidence of AKI. Of enrolled patients, 1,825/2,101 (87%) were adults, 1,117/2,101 (53%) were females, 399/2,101 (19%) were from Bolivia, 813/2,101 (39%) were from Malawi, and 889/2,101 (42%) were from Nepal. The age of enrolled patients ranged from 1 month to 96 years, with a mean of 43 years (SD 21) and a median of 43 years (IQR 27–62). Hypertension was the most common comorbidity (418/2,101; 20%). At enrollment, 197/2,101 (9.4%) had CKD, and 1,199/2,101 (57%) had AKD. AKI developed in 30% within 7 days. By 1 month, 268/978 (27%) patients in the observation phase and 203/1,123 (18%) in the intervention phase were lost to follow-up. In the intervention phase, more patients received fluids (observation 714/978 [73%] versus intervention 874/1,123 [78%]; 95% CI 0.63, 0.94; *p =* 0.012), hospitalization was reduced (observation 578/978 [59%] versus intervention 548/1,123 [49%]; 95% CI 0.55, 0.79; *p <* 0.001), and admitted patients with severe AKI did not show a significantly lower mortality during follow-up (observation 27/135 [20%] versus intervention 21/178 [11.8%]; 95% CI 0.98, 3.52; *p =* 0.057). Of 504 patients with kidney function assessed during the 6-month follow-up, de novo CKD arose in 79/484 (16.3%), with no difference between the observation and intervention phase (95% CI 0.91, 2.47; *p =* 0.101). Overall mortality was 273/2,101 (13%) and was highest in those who had CKD (24/106; 23%), followed by those with AKD (128/760; 17%), AKI (85/628; 14%), and NKD (36/607; 6%). The main limitation of our study was the inability to determine the actual incidence of kidney dysfunction in the health centers as it was not feasible to screen all the patients due to the high numbers seen daily.

**Conclusions:**

This multicenter, non-randomized feasibility study in low-resource settings demonstrates that it is feasible to implement a comprehensive program utilizing POC testing and protocol-based management to improve the recognition and management of AKI and AKD in high-risk patients in primary care.

## Abstract 2

Etienne Macedo and colleagues report on a point-of-care testing program for acute kidney injury and disease in high-risk primary care patients.

## Author summary

**Why was this study done?:**

- The study was designed to assess the feasibility of implementing interventions to optimize care of acute kidney injury (AKI).
- We used a comprehensive 5R approach—risk, recognition, response, renal support, and rehabilitation—to test the intervention in resource-constrained regions in Africa, Asia, and Latin America.

**What did the researchers do and find?:**

- Patients seen in community healthcare centers were screened and assigned a risk level for AKI based on their presenting signs and symptoms. Patients with moderate to high risk were approached for consent and enrolled in the study, underwent kidney function assessment, and were followed for their disposition and outcomes.
- Kidney function was assessed using point-of-care (POC) tests that included a test strip for measuring creatinine level in the blood using a portable device and a urine dipstick test to evaluate for proteinuria.

**What do these findings mean?:**

- The ISN 0by25 trial successfully demonstrated the utility of a symptom-based health assessment risk score coupled with a POC creatinine and urine dipstick test in early recognition of kidney disease and appropriate triaging and management of patients presenting to primary healthcare centers in low-income countries. Kidney dysfunction was associated with an increased risk of mortality, which was higher in patients with a moderate severity of AKI. Recognition and management of patients was facilitated by the combination of the POC test and guidance through teleconsultation.

## Introduction

The incidence of acute kidney injury (AKI) has substantially increased over the past 2 decades, with the fastest growth occurring in low- and middle-income countries (LMICs). Once AKI is established, it is expensive to manage, prolongs hospitalization, and is associated with increased mortality and risk of development of chronic kidney disease (CKD) [1–5]. The burden of AKI is particularly high in LMICs, where a lack of early identification and limited treatment worsens patient outcomes [6]. Given the healthcare system limitations prevalent in LMICs, it is crucial to provide early intervention designed to avoid progression to severe AKI.

Recognizing AKI as a growing global problem, the International Society of Nephrology (ISN) launched in 2013 the AKI 0by25 initiative [7] with the ambitious goal of reaching 0 preventable AKI deaths worldwide by 2025 [8]. The first project of the initiative, the Global Snapshot [9] recorded information on over 4,000 pediatric and adult patients with AKI encountered in regular practice by 372 physicians from 72 countries over 10 weeks in the last quarter of 2014 [10]. Data from the Global Snapshot revealed differences in recognition, management, and outcomes of AKI in different healthcare settings. Community-acquired AKI was more common than hospital-acquired AKI in LMICs, and was associated with more severe AKI at presentation and worse outcomes.

We hypothesized that a lack of awareness of and a lack of access to diagnostic and therapeutic care contributed to the disparities found in the outcomes of community-acquired AKI. To address these issues, the ISN 0by25 trial was designed to assess the feasibility of implementing an education and training program in low-resource settings and to introduce the use of point-of-care (POC) serum creatinine testing to optimize the identification and management of AKI in the community.

## Methods

### Design and setting

The ISN funded the study, which recruited patients from 1 October 2016 to 29 September 2017, in Asia (Dharan, Nepal), Africa (Blantyre, Malawi), and Latin America (Cochabamba, Bolivia). Each site comprised a cluster of healthcare centers (HCCs), including 3–4 community health centers, 1 district hospital, and 1 regional referral hospital serving the population around the site area (S1 Center Characteristics). This study was designed as a non-randomized exploratory study in 3 phases as we did not have data on the prevalence of kidney disease and incidence of AKI, acute kidney disease (AKD), and CKD in the community health settings in these countries, and it was not possible to do a sample size calculation. We utilized a 4-month observation phase to establish the baseline state of healthcare delivery and prevalence of kidney disease, followed by a 2-month training phase to equip the medical workers with the knowledge to use the POC test and teleconsultations, and a 6-month intervention phase focused on assessing the practicality of implementing the POC test and teleconsultation for triaging patients across the centers.

### Ethics statement

The study was approved by the institutional review board and the ethics committee of University of California San Diego, and by the 3 local sites.

### Patient population

Adult and pediatric patients with symptoms of conditions associated with increased risk of AKI who presented to the HCC or emergency department of the involved hospitals were assessed for eligibility to participate in the study. In some of the HCCs and emergency departments, the number of patients seen daily was high, and it was not possible to screen all patients. In these centers, the study coordinators screened patients who, based on their initial assessment, were more likely to have a higher risk score. We used a clinical assessment tool to predict the development of severe AKI (defined as KDIGO stage 2 or 3), the need for dialysis, and mortality based on data from the ISN Global Snapshot study [9]. We used data from 3,283 adult patients in the Global Snapshot study with available signs and symptoms at presentation to the clinic or hospital before AKI diagnosis. We utilized the presence of decreased urine output, hypotension/shock, coma, jaundice, anemia, confusion, dyspnea, symptoms of respiratory infection, petechiae, ecchymosis, bleeding, and hypertension (in pregnant women) to construct a risk score based on the odds ratio (OR) estimate effect values of the regression model (S1 Risk Score). The research team recorded all the pertinent clinical data in a mobile-enabled, web-based, open-source database (KEEP). The KEEP database was accessed through computers (PC and Mac), Android tablets, and cell phones. The risk score was calculated by adding 1 point for each individual symptom and classified patients as high (>6), moderate (3–5), or low risk (0–2) for developing AKI. Patients with moderate or high risk signed an informed consent before enrolling in the study. Written consent was obtained from the patient or surrogate, parent/guardian in the case of minors or those in whom the capacity to make an informed decision was impaired. Patients receiving renal replacement therapy (dialysis or kidney transplantation) were not eligible for the study.

### Observation phase (4 months)

During the observation phase, consenting moderate- and high-risk patients had a POC test for serum creatinine (StatSensor Xpress Creatinine, Nova Biomedical, Waltham, MA, US) and a urine dipstick for proteinuria performed by the research coordinator. POC results were given to the healthcare provider at the site. Pertinent clinical data were recorded in a mobile-enabled secure online platform [9]. In hospitalized patients, the serum creatinine POC test was repeated on day 2 after enrollment, and available clinical and laboratory data were recorded daily during hospitalization. All patients were scheduled to return for follow-up at 7 days and 1, 3, and 6 months, when serum creatinine was remeasured. If the patient did not return for follow-up in the HCC, research coordinators attempted contact by home visit or phone. During the observation phase, patients were tracked throughout the healthcare evaluation, but no specific clinical guidance was provided.

### Education/Training phase (2 months)

Following the observation phase, healthcare providers at each of the participating sites received training on risk assessment, recognition, and management of AKI. In Malawi and Nepal, not all healthcare providers were medical doctors; thus, the terminology includes nurses and medical officers. They were also trained on the use of the POC tests, interpretation of test results, and teleconsultation protocols with physicians at the referral hospitals. Physicians were trained on the STOP AKI protocol (S1 STOP Protocol) and teleconsultation protocols to guide the HCC healthcare providers on initial treatment and triaging for ongoing care and follow-up. Communications between the healthcare provider and the teleconsultation physicians at each site using text messaging were tested and confirmed using mobile digital technology (S1 Fig).

### Intervention phase (6 months)

During this phase, risk assessment and screening were performed in the same manner as during the observational phase. Test results were given to the healthcare providers, who engaged the teleconsultation physician for real-time patient management guidance. Teleconsultation physicians reviewed the clinical information and communicated their recommendations for implementation via the teleconsultation evaluation form (see S1 Fig). Research coordinators facilitated these interactions and recorded treatment and triage decisions.

### Definitions

Kidney function at enrollment was defined based on prior history, serum creatinine assessed by POC test, and urinalysis. Patients were stratified into 3 groups: no kidney disease (NKD), AKD, and CKD. Patients with an estimated glomerular filtration rate (eGFR) < 60 ml/min/1.73 m^2^, calculated by the Chronic Kidney Disease Epidemiology Collaboration (CKD-EPI) formula based on serum creatinine within the previous 12 months, were defined as having CKD. AKD was defined using the operational definition proposed by the KDIGO AKI Work Group using structural and functional criteria: the presence of kidney damage for <3 months (proteinuria) and/or eGFR at enrollment < 60 ml/min/1.73 m^2^ without a prior history of these abnormalities or structural damage (proteinuria). Patients not fulfilling these criteria were classified as NKD (Table 1). Based on serum creatinine within 7 days of presentation, patients in each of the 3 groups were further classified as having AKI if they exhibited an increase or decrease in serum creatinine of ≥26.5 μmol/l within 48 hours or an increase to >1.5 times the reference serum creatinine value within 7 days compared to the value at enrollment [11]. In patients without a history of CKD, we used the first serum creatinine value at enrollment to detect changes in kidney function. The patient course following the initial encounter was classified as discharged home if the patient left the HCC within 24 hours or admitted if the hospital stay was >24 hours. Serum creatinine POC tests were repeated at 48 hours if the patient was hospitalized, and at day 7 and months 1, 3, and 6. In patients who developed AKI or had normal eGFR at enrollment, the persistence of renal dysfunction beyond 7 days and up to 90 days was also classified as AKD. Patients were further classified as having new-onset CKD if eGFR at 3 and/or 6 months was <60 ml/min/1.73 m^2^, or as having CKD progression (in those pre-existing CKD) if eGFR decreased from the value at enrollment (Table 1). AKI patients were further classified as having complete, partial, or no AKI recovery. Recovery was defined as complete when serum creatinine returned to the same as or lower than the enrollment value, or partial when serum creatinine was lower than peak but higher than the enrollment value.

**Table 1:** Renal function status definitions at different time points.

Terminology	Definition	Time of assessment
Chronic kidney disease (CKD)	Prior knowledge of CKD or previous baseline serum creatinine within 12 months yielding an eGFR < 60 ml/min/1.73 m^2^ as calculated by the CKD-EPI equation	Enrollment, 7 days, 1 month, and 3 months
Acute kidney disease (AKD)	Patients with unknown history of renal dysfunction presenting with 1 or more of the following:	
	• sCr at enrollment corresponding to an eGFR < 60 ml/min/1.73 m^2^ as calculated by the CKD-EPI equation	Enrollment
	• Urine dipstick at enrollment with proteinuria (≥1+)	Enrollment
	• Patients with AKD at enrollment but not meeting the criteria for AKI within 7 days	7 days and 1 month
	• Patient with NKD at 7 days with an eGFR < 60 ml/min/1.73 m^2^ or proteinuria at 1 month	1, 3, and 6 months
	• Patient with persistent AKI with sCr greater than reference sCr for AKI diagnosis (partial recovery) or dialysis-dependent or sCr corresponding to eGFR < 15 ml/min/1.73 m^2^ (no recovery)	1, 3, and 6 months
Acute kidney injury (AKI)	Increase in sCr by ≥26.5 μmol/l within 48 hours or increase to >1.5 times the reference sCr within 7 days or decrement in enrollment sCr by ≥26.5 μmol/l within 48 hours or decrement to >1.5 times the enrollment sCr within 7 days	7 days
AKI recovery	Complete: sCr at 1 month less than or equal to reference sCr for AKI diagnosis (in patients with AKI by increment in sCr)	1 month
No kidney disease (NKD)	Patients with no history of CKD, no proteinuria, and sCr corresponding to an eGFR ≥ 60 ml/min/1.73 m^2^ as calculated by the CKD-EPI equation	Enrollment, 7 days, 1 month, and 3 months
New-onset CKD	NKD, AKI, and AKD groups only: sCr persistently elevated and corresponding to an eGFR < 60 ml/min/1.73 m^2^ as calculated by the CKD-EPI equation	3 and 6 months
CKD progression	CKD group only: decline in eGFR at 3 or 6 months relative to enrollment eGFR	3 and 6 months

CKD-EPI, Chronic Kidney Disease Epidemiology Collaboration; eGFR, estimated glomerular filtration rate; sCr, serum creatinine.

### Outcome measures

We assessed the frequency of development of AKI, AKD, CKD, and the need for renal replacement therapy in the observational and interventional phases. Kidney recovery and mortality were assessed at 1, 3, and 6 months. Patients who did not return to the HCC or hospital for their follow-up appointment were tracked based on their phone number or house address. Not all patients had that information recorded, and that was one of the main reasons for the loss of follow-up.

### Statistical analysis

We present continuous variables as mean (SD) or median (IQR), as appropriate. We used the Kolmogorov–Smirnov test to check data normality. Continuous variables are compared with the 2-sample *t* test and Mann–Whitney U test for normally distributed and non-normally distributed variables, respectively. Categorical variables are presented as proportions and compared with Fisher’s exact test; the 95% confidence interval for testing hypotheses about the OR was calculated using the method by Fay [12]. We used Fisher’s exact test with the Bonferroni–Holm correction for comparison across more than 2 groups. Statistical tests were 2-sided, and *p <* 0.05 was considered statistically significant. The analysis was conducted using SPSS 25 (IBM SPSS Statistics) and R version 3.2.1.

## Results

### Patient characteristics and course

**Fig 1:**
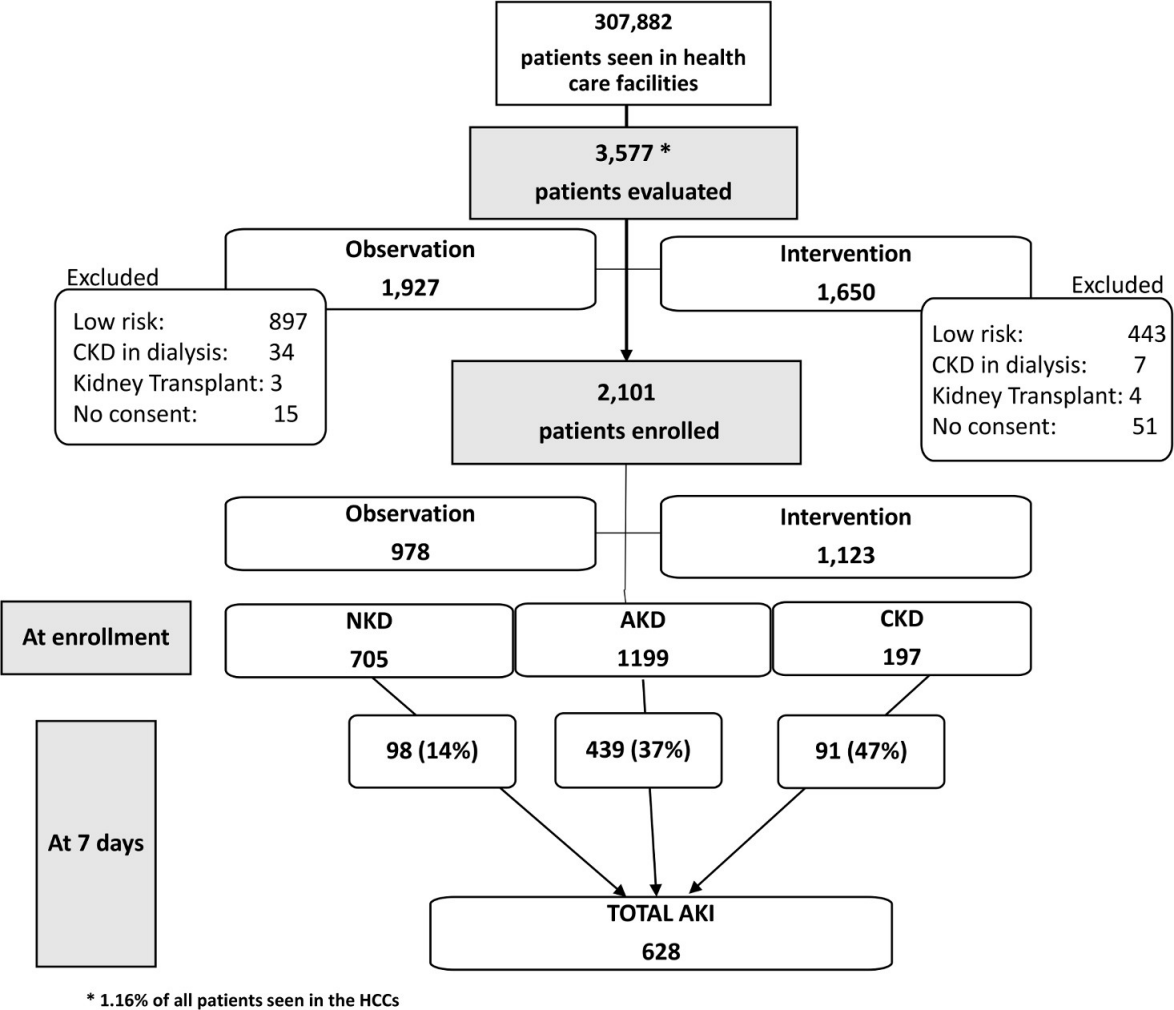
Study flowchart and renal function status at enrollment and 7 days. At enrollment 1,199 (57%) of patients were classified as having AKD, 197 (9%) as having CKD, and 705 (34%) as having NKD. After 7 days, 98 (14%) NKD patients, 439 (37%) AKI patients, and 91 (47%) CKD patients had developed AKI. AKD, acute kidney disease; AKI, acute kidney injury; CKD, chronic kidney disease; HCC, healthcare center; NKD, no kidney disease.

We screened 3,577 patients in the HCCs of the 3 participating countries (S2 Table); 1,927 in the observation and 1,650 in the intervention phase, representing 1.36% and 0.98%, respectively, of the population seen in the participant centers during that period (Fig 1). We recognize that the patient characteristics and standard of care were heterogeneous within the 3 participating countries and among the centers (S1 Table). We intend to publish a more detailed analysis of these different factors and how they could have affected patient outcomes in a separate paper.

Of screened patients, 1,340 were classified as having low risk for AKI: 897 (46%) in the observation phase and 443 (26%) in the intervention phase. Of patients classified as having moderate or high risk for AKI, 2,101 patients were enrolled: 978 in the observation phase (47%) and 1,123 in the intervention phase (53%). Of enrolled patients, 1,825 (87%) were adults, 1,117 (53%) were females, 399 were from Bolivia, 813 were from Malawi, and 889 were from Nepal (S1 Table). The age of enrolled patients ranged from 1 month to 96 years, with a mean of 43 years (SD 21) and median of 43 (IQR 27–62); of 275 (13%) children, mean age was 8 years (Table 2). Within both phases, information at enrollment on the prior state of kidney health (serum creatinine, proteinuria, imaging studies) was available in 351 patients (16.7%), of whom 197 were classified as having CKD. At enrollment, 1,199 (57%) patients had evidence of alterations in kidney function and/or structure (AKD). Reduced eGFR based solely on their initial POC creatinine was found in 789 patients, proteinuria was found in 192 patients, and a combination of reduced eGFR and proteinuria was found in 218 patients. A total of 705 participants (34%) were classified as having NKD.

**Table 2:** Patient characteristics at enrollment in the observation and intervention phase.

Characteristic	Category	Observation *N =* 978	Intervention *N =* 1,123	All *N =* 2,101	*p*-Value*
Age	Children (<18 years)	130 (13.3%)	142 (12.7%)	272 (13.0%)	0.70
	Median age—children (years)	7.0 (3.2–13.0)	6.5 (1.6–13.7)	7.0 (3.0–13.0)	0.19
	Adult	848 (86.7%)	977 (87.3%)	1,825 (87.0%)	0.70
	Median age—adults (years)	46 (33–63)	47 (33–64)	47 (33–65)	0.26
Sex	Male	468 (48.0%)	515 (46.0%)	983 (47.0%)	0.41
	Female	510 (52.1%)	607 (54.1%)	1,117 (53.2%)	
Ethnicity	Quechua	57 (5.8%)	92 (8.2%)	149 (7.1%)	0.06
	Hispanic	98 (10.0%)	135 (12.0%)	233 (11.1%)	
	Asian	426 (43.6%)	462 (41.1%)	888 (42.3%)	
	African	390 (40.0%)	423 (37.7%)	813 (38.7%)	
	Other	5 (0.5%)	11 (1.0%)	16 (0.8%)	
Comorbidities	Diabetes mellitus	117 (12.0%)	139 (12.4%)	256 (12.2%)	0.79
	Liver disease	34 (3.5%)	53 (4.7%)	87 (4.1%)	0.19
	Heart disease	50 (5.1%)	38 (3.4%)	88 (4.2%)	0.05
	Lung disease	75 (7.7%)	44 (3.9%)	119 (5.7%)	<0.001
	HIV	144 (14.7%)	229 (20.4%)	373 (17.8%)	<0.001
	Previous diagnosis of anemia	109 (11.1%)	146 (13.0%)	255 (12.1%)	0.21
	Cancer	15 (1.5%)	21 (1.9%)	36 (1.7%)	0.61
	Hypertension	203 (20.8%)	215 (19.1%)	418 (19.9%)	0.38

Data are shown as *n* (%) or median (IQR). Three children did not have a precise age. One adult did not have a precise age.

*p-Value for difference between observation and intervention phase. Fisher’s exact test was used for categorical variables, and a 2-sample *t* test was used for continuous variables.

The most common comorbidities present were hypertension (20%), human immunodeficiency virus (HIV) infection (18%), and diabetes mellitus (12%). The vast majority of the patients with HIV infection (365; 98%) were from Malawi. Generalized weakness was the most common complaint, followed by dehydration and infection. Decreased urinary output was encountered in about one-third of the patients. Patients with NKD had a lower frequency of comorbidities and were less likely to present with dehydration (S1 Table).

Seven-day follow-up was complete in 1,754 (83.5%) of the patients, including 88% of those admitted and 79% of those sent home. AKI based on serum creatinine KDIGO criteria in the first 7 days occurred in 628 (30%) of the patients: 439 (37%) from the AKD group, 91 (47%) from the CKD group, and 98 (14%) from the NKD group. AKI based on serum creatinine decline was encountered in 261 (41.5%), including 207 (47%) in the AKD group, 30 (33%) in the CKD group, and 24 (25%) in the NKD group. A higher percentage of patients with AKI (388; 62%) was identified in the intervention phase than in the observation phase (240; 38%, *p <* 0.001). We did not record adverse events as study coordinators did not have the knowledge and training to differentiate complications of the disease state or the standard treatment from adverse events. The local principal investigators reviewed all the cases in each center, and based on their assessment, patients did not incur any adverse events during the study.

Overall, 975 (46%) patients were sent home following the initial evaluation at the HCC, while 1,126 (54%) were admitted to a healthcare facility. In the intervention phase, patients spent a longer time in the HCC (intervention 4.0 hours versus observation 1.6 hours; *p* < 0.001; OR 95% CI 0.55, 0.79), and the proportion of patients sent home was significantly higher (intervention 51% versus observation 41%; *p <* 0.001; OR 95% CI –2.12, –1.00) (Table 3). Most patients (76%) received fluid therapy in the healthcare facility; 39% received oral fluids, and 70% intravenous fluids, following enrollment (S3 Table). In the intervention phase more patients received fluids (observation 73% versus intervention 78%; OR 95% CI 0.63, 0.94; *p =* 0.012), and patients received a higher volume, both orally and intravenously. Diuretics were given in 170 (9%) of the patients, with no difference between the 2 phases.

**Table 3:** Course of patients in observation and intervention phases.

Clinical or treatment variable	All *N =* 2,101	Observation *N =* 978 (46%)	Intervention *N =* 1,123 (53%)	*p*-Value
***Renal function at enrollment***				
**CKD**^**1**^	197 (9.4%)	80 (8.2%)	117 (10.4%)	0.085
With proteinuria	66 (3.9%)	39 (5.1%)	27 (2.9%)	0.023
**AKD**	1,199 (57.1%)	552 (56.4%)	647 (57.6%)	0.596
Based on sCr alone	789 (65.8%)	338 (61.2%)	451 (69.7%)	<0.001
Based on sCr and proteinuria	218 (18.2%)	95 (17.2%)	123 (19.0%)	
Based on proteinuria alone	192 (16.0%)	119 (21.6%)	73 (11.3%)	
**NKD**	705 (33.6%)	346 (35.4%)	359 (32.0%)	0.105
***Renal function at 7 days ***				
**No AKI**	1,473 (70.1%)	738 (75.5%)	735 (65.4%)	<0.001
**CKD**	106 (53.8%)	50 (62.5%)	56 (47.9%)	0.058
**AKD**	760 (63.4%)	375 (67.9%)	385 (59.5%)	0.003
**NKD**	607 (86.1%)	313 (90.5%)	294 (81.9%)	0.001
**AKI**	628 (29.9%)	240 (24.5%)	388 (34.6%)	<0.001
Stage 1	252 (40.1%)	91 (37.9%)	161 (41.5%)	0.403
Stage 2	138 (22.0%)	46 (19.2%)	92 (23.7%)	0.198
Stage 3	238 (37.9%)	103 (42.9%)	135 (34.8%)	0.043
Severe AKI (stage2/3)	376 (59.9%)	149 (62.1%)	227 (58.5%)	0.403
***Disposition***				
**Sent home**	975 (46.4%)	400 (40.9%)	575 (51.2%)	<0.001
**Admitted**	1,126 (53.6%)	578 (59.1%)	548 (48.8%)	
NKD	208 (18.5%)	125 (21.6%)	83 (15.1%)	0.006
AKI	518 (46.0%)	211 (36.5%)	307 (56.0%)	<0.001
AKD	351 (31.2%)	208 (36.0%)	143 (26.1%)	<0.001
CKD	49 (4.4%)	34 (5.9%)	15 (2.7%)	0.012
**Time (hours) in healthcare facility**^**2**^	28.69 (4.25–120.00)	27.00 (3.52–120.78)	31.00 (4.85–118.00)	0.243
Sent home	3.80 (1.00–6.07)	1.56 (0.21–5.53)	4.00 (2.00–6.48)	<0.001
Admitted	96.00 (46.70–167.13)	95.05 (29.08–177.35)	97.75 (49.92–158.95)	0.167
***Dialysis requirement***				
**Dialysis indication**	130 (6.2%)	54 (5.5%)	76 (6.8%)	0.276
**Dialyzed**	63 (3.0%)	32 (3.3%)	31 (2.8%)	0.523
AKD	14 (22.2%)	11 (34.4%)	3 (9.7%)	0.027
AKI	43 (68.3%)	20 (62.5%)	23 (74.2%)	
CKD	6 (9.5%)	1 (3.1%)	5 (16.1%)	
**Dialysis indication, but not dialyzed**	67 (3.2%)	22 (2.2%)	45 (4.0%)	0.025
AKD	31 (46.3%)	11 (50.0%)	20 (44.4%)	0.768
AKI	21 (31.3%)	5 (22.7%)	16 (35.6%)	
CKD	12 (17.9%)	5 (22.7%)	7 (15.6%)	
NKD	3 (4.5%)	1 (4.5%)	2 (4.4%)	

Data are shown as *n* (%) or median (IQR). *p*-Values refer to the difference between the observation and intervention phases.

^1^Compares the proportion of CKD patients between the observation and intervention phases.

^2^*p*-Values associated with time in healthcare facility (all, sent home, and admitted) are based on Mann–Whitney U test.

AKD, acute kidney disease; AKI, acute kidney injury; CKD, chronic kidney disease; sCr, serum creatinine.

Dialysis was performed in 63 patients, with no difference in the frequency of dialysis indication or provision between the intervention and observation phases. Sixty-seven additional patients met criteria for dialysis but did not receive it, 22 (2.2%) in the observation phase versus 45 (4%) in the intervention phase (Table 3).

### Outcomes

#### Kidney function recovery

By 1 month, 268/978 (27%) patients in the observation phase and 203/1,123 (18%) in the intervention phase were lost to follow-up. Of patients with clinical information at 1 month, serum creatinine was available in 981 patients (69%): 259 with NKD at 7 days, 402 with AKI, 282 with AKD, and 38 with CKD. Among patients with NKD at 7 days, 20/258 (7.8%) had eGFR less than 60 ml/min/1.73 m^2^ at 1 month. Among those with AKI in whom serum creatinine was measured (*n =* 402) and with no history of CKD, serum creatinine at 1 month was lower than at enrollment in 336/402 (84%). Of patients with AKD (at 7 days), serum creatinine at 1 month was lower than at enrollment in 241/282 (85.5%). New onset of CKD over the 6-month follow-up period was detected in 37 of 230 patients (16%) (Table 4). Overall, there was no difference in renal recovery or progression to CKD in the observation and intervention phase.

**Table 4:** CKD development and progression during follow-up in the observation and intervention phase based on renal function status at 7 days.

Renal recovery	Overall	Observation	Intervention	*p*-Value
**At 3 months**				
Overall recovery	344/425 (80.9%)	119/152 (78.3%)	225/273 (82.4%)	0.305
New-onset AKD on NKD	5/140 (3.6%)	2/54 (3.7%)	3/86 (3.5%)	1
CKD progression	5/10 (50.0%)	2/4 (50.0%)	3/6 (50.0%)	1
New-onset CKD in AKD without AKI	38/115 (33.0%)	19/45 (42.2%)	19/70 (27.1%)	0.107
New-onset CKD in AKI	28/160 (17.5%)	8/49 (16.3%)	20/111 (18.0%)	1
De novo CKD	66/415 (15.9%)	27/148 (18.2%)	39/267 (14.6%)	0.331
**At 6 months**				
Overall recovery	189/235 (80.4%)	73/98 (74.5%)	116/137 (84.7%)	0.066
New-onset AKD on NKD	4/89 (4.5%)	2/39 (5.1%)	2/50 (4.0%)	1
CKD progression	4/5 (80.0%)	1/2 (50.0%)	3/3 (100.0%)	0.4
New-onset CKD in AKD without AKI	24/68 (35.3%)	14/32 (43.8%)	10/36 (27.8%)	0.208
New-onset CKD in AKI	13/73 (17.8%)	7/25 (28.0%)	6/48 (12.5%)	0.118
De novo CKD	37/230 (16.1%)	21/96 (21.9%)	16/134 (11.9%)	0.047
**Overall**				
Overall recovery	398/495 (80.4%)	145/190 (76.3%)	253/305 (83.0%)	0.081
New-onset AKD on NKD	7/172 (4.1%)	4/72 (5.6%)	3/100 (3.0%)	0.454
CKD progression	6/11 (54.5%)	2/4 (50.0%)	4/7 (57.1%)	1
New-onset CKD in AKD without AKI	47/138 (34.1%)	25/61 (41.0%)	22/77 (28.6%)	0.149
New-onset CKD in AKI	32/174 (18.4%)	12/53 (22.6%)	20/121 (16.5%)	0.396
De novo CKD	79/484 (16.3%)	37/186 (19.9%)	42/298 (14.1%)	0.101

Data are shown as *n/N* (%). *p-*Values refer to the comparison between the observation and intervention phases.

AKD, acute kidney disease; AKI, acute kidney injury; CKD, chronic kidney disease.

#### Mortality

**Fig 2:**
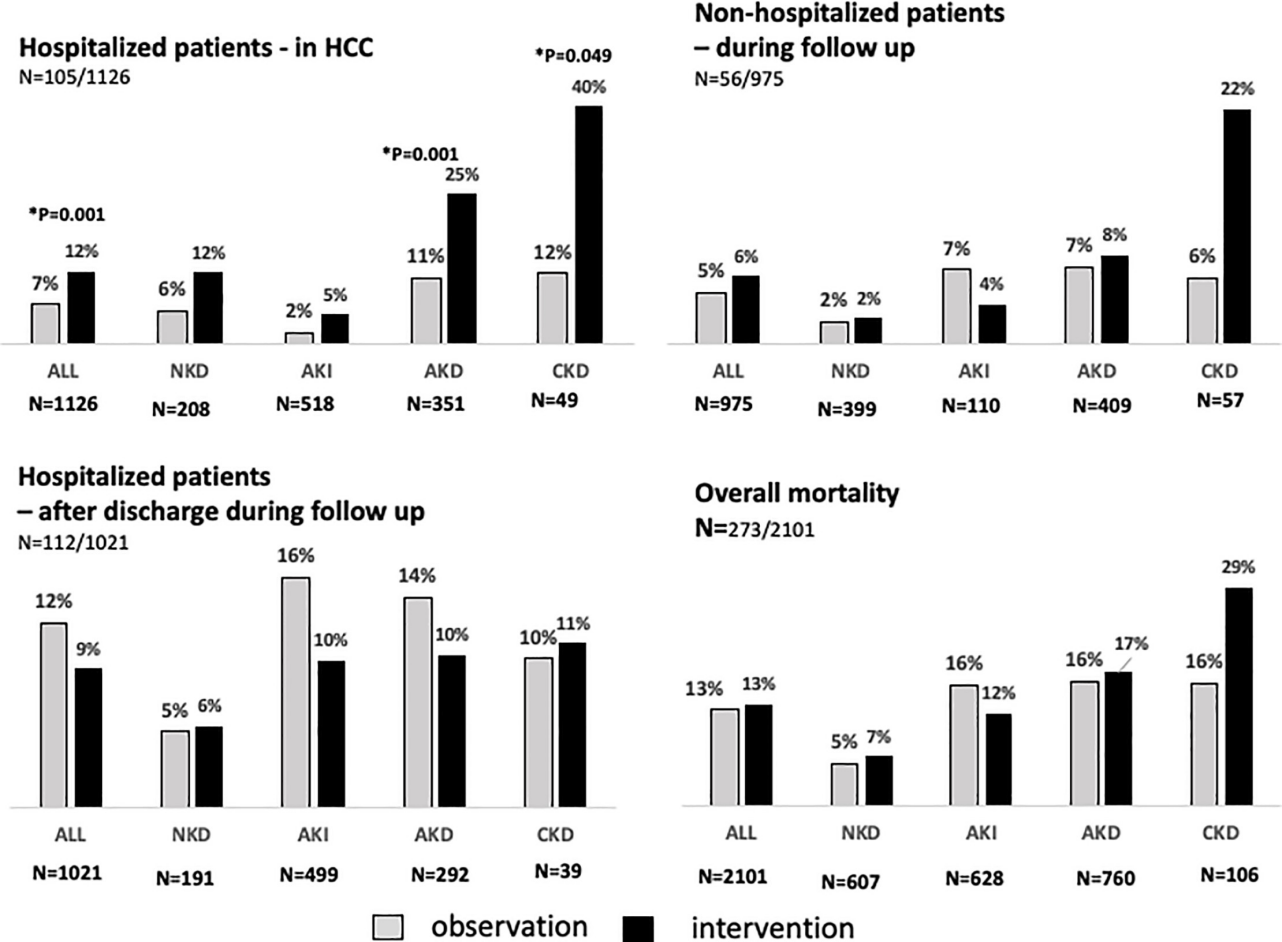
Mortality rate in hospitalized and non-hospitalized patients during the observation and intervention phases in HCCs. *Statistically significant *p-*values comparing observation and intervention phase in each group are shown in the figure; other values were nonsignificant (*p* ≥ 0.05). AKD, acute kidney disease; AKI, acute kidney injury; CKD, chronic kidney disease; HCC, healthcare center; NKD, no kidney disease.

Overall mortality was 273/2,101 (13%), and 105 of 1,126 hospitalized patients died in the hospital—32 within 24 hours and the remaining 112 during the 6 months of follow-up (Fig 2). Overall mortality increased from 7% (156) at 7 days to 10% (206) at 1 month and 12% (258) at 3 months.

Hospitalization was reduced during the intervention phase (observation 578/978 [59%] versus intervention 548/1,123 [49%]; 95% CI 0.55, 0.79; *p <* 0.001). In-hospital mortality of patients classified as having AKD at 7 days was 17% (59 individuals), versus 4% in AKI patients (Table 5). The after-discharge mortality rate in AKD patients who were admitted to healthcare facilities was similar in the observation and intervention phase (observation 14% [30/212] versus intervention 9% [11/125]; *p =* 0.170) (Fig 2). There was no difference in overall mortality between AKI patients in the observation and intervention phase. Still, in hospitalized patients with severe AKI (KDIGO stages 2 and 3), there was a nonsignificant reduction of mortality during the intervention phase (observation 20% [27/135] versus intervention 11.8% [21/178]; *p =* 0.057; 95% CI 0.98, 3.52) (Table 5).

**Table 5:** Mortality during the follow-up period by AKI status and stage in the observation and intervention phases.

Mortality and clinical/treatment variable	All *N =* 2,101	Observation *N =* 978 (46)	Intervention *N =* 1,123 (53)	*p*-Value
**Mortality in hospital—hospitalized patients**	105/1,126 (9.3%)	38/578 (6.6%)	67/548 (12.2%)	0.001
NKD	17/208 (8.2%)	7/125 (5.6%)	10/83 (12.0%)	0.122
No AKI	86/608 (14.1%)	34/367 (9.3%)	52/241 (21.6%)	<0.001
AKI	19/518 (3.7%)	4/211 (1.9%)	15/307 (4.9%)	0.096
Stage 1	5/191 (2.6%)	1/73 (1.4%)	4/118 (3.4%)	0.651
Stage 2	3/116 (2.6%)	1/41 (2.4%)	2/75 (2.7%)	1
Stage 3	11/211 (5.2%)	2/97 (2.1%)	9/114 (7.9%)	0.068
Severe AKI (stages 2 and 3)	14/327 (4.3%)	3/138 (2.2%)	11/189 (5.8%)	0.165
Dialysis indication but not dialyzed	19/45 (42.2%)	5/15 (33.3%)	14/30 (46.7%)	0.526
Dialyzed	2/58 (3.4%)	1/31 (3.2%)	1/27 (3.7%)	1
AKD	59/351 (16.8%)	23/208 (11.1%)	36/143 (25.2%)	<0.001
CKD	10/49 (20.4%)	4/34 (11.8%)	6/15 (40.0%)	0.049
**Mortality during follow-up—hospitalized patients**	112/1,021 (11.0%)	67/540 (12.4%)	45/481 (9.4%)	0.133
NKD	10/191 (5.2%)	6/118 (5.1%)	4/73 (5.5%)	1
No AKI	51/522 (9.8%)	35/333 (10.5%)	16/189 (8.5%)	0.54
AKI	61/499 (12.2%)	32/207 (15.5%)	29/292 (9.9%)	0.072
Stage 1	13/186 (7.0%)	5/72 (6.9%)	8/114 (7.0%)	1
Stage 2	17/113 (15.0%)	10/40 (25.0%)	7/73 (9.6%)	0.051
Stage 3	31/200 (15.5%)	17/95 (17.9%)	14/105 (13.3%)	0.436
Severe AKI (stages 2 and 3)	48/313 (15.3%)	27/135 (20.0%)	21/178 (11.8%)	0.057
Dialysis indication but not dialyzed	6/26 (23.1%)	2/10 (20.0%)	4/16 (25.0%)	1
Dialyzed	15/56 (26.8%)	6/30 (20.0%)	9/26 (34.6%)	0.243
AKD	37/292 (12.7%)	26/185 (14.1%)	11/107 (10.3%)	0.465
CKD	4/39 (10.3%)	3/30 (10.0%)	1/9 (11.1%)	1
**Mortality during follow-up—non-hospitalized patients**	56/975 (5.7%)	19/400 (4.8%)	37/575 (6.4%)	0.327
NKD	9/399 (2.3%)	4/188 (2.1%)	5/211 (2.4%)	1
No AKI	51/865 (5.9%)	17/371 (4.6%)	34/494 (6.9%)	0.189
AKI	5/110 (4.5%)	2/29 (6.9%)	3/81 (3.7%)	0.606
Stage 1	3/61 (4.9%)	2/18 (11.1%)	1/43 (2.3%)	0.205
Stage 2	0/22 (0.0%)	0/5 (0.0%)	0/17 (0.0%)	1
Stage 3	2/27 (7.4%)	0/6 (0.0%)	2/21 (9.5%)	1
Severe AKI (stages 2 and 3)	2/49 (4.1%)	0/11 (0.0%)	2/38 (5.3%)	1
Dialysis indication but not dialyzed	4/22 (18.2%)	1/7 (14.3%)	3/15 (20.0%)	1
Dialyzed	1/5 (20.0%)	0/1 (0.0%)	1/4 (25.0%)	1
AKD	32/409 (7.8%)	12/167 (7.2%)	20/242 (8.3%)	0.852
CKD	10/57 (17.5%)	1/16 (6.2%)	9/41 (22.0%)	0.253
**Overall mortality**	273/2,101 (13.0%)	124/978 (12.7%)	149/1,123 (13.3%)	0.697
NKD	36/607 (5.9%)	17/313 (5.4%)	19/294 (6.5%)	0.61
No AKI	188/1,473 (12.8%)	86/738 (11.7%)	102/735 (13.9%)	0.212
AKI	85/628 (13.5%)	38/240 (15.8%)	47/388 (12.1%)	0.189
Stage 1	21/252 (8.3%)	8/91 (8.8%)	13/161 (8.1%)	0.817
Stage 2	20/138 (14.5%)	11/46 (23.9%)	9/92 (9.8%)	0.039
Stage 3	44/238 (18.5%)	19/103 (18.4%)	25/135 (18.5%)	1
Severe AKI (stages 2 and 3)	64/376 (17.0%)	30/149 (20.1%)	34/227 (15.0%)	0.208
Dialysis indication but not dialyzed	29/67 (43.3%)	8/22 (36.4%)	21/45 (46.7%)	0.447
Dialyzed	18/63 (28.6%)	7/32 (21.9%)	11/31 (35.5%)	0.274
AKD	128/760 (16.8%)	61/375 (16.3%)	67/385 (17.4%)	0.699
CKD	24/106 (22.6%)	8/50 (16.0%)	16/56 (28.6%)	0.164

Numbers are *n* and %. *p-*Values refer to the comparison between observation versus intervention phase.

AKD, acute kidney disease; AKI, acute kidney injury; CKD, chronic kidney disease; NKD, no kidney disease.

At 6 months, overall mortality was 273 (13%) and was higher in hospitalized patients (19%; 217/1,126) than in non-hospitalized patients (6%; 56/975) (Table 5; *p <* 0.001; OR 95% CI 2.88, 5.34). Patients with any form of kidney dysfunction had higher mortality than those with NKD (NKD 6% [26/445], AKI 14% [85/628], AKD 15% [138/922], and CKD 23% [24/106], all *p <* 0.001; OR 95% CI 0.23, 0.54]. Patients with more severe AKI (KDIGO stages 2 and 3) had significantly (*p =* 0.0139) higher mortality (17% [64/376]) than individuals with no AKI (13%) or stage 1 AKI (8% [209/1,725]). Among non-hospitalized patients, the CKD group had the highest mortality (18% [10/57]) (Fig 2). AKD patients who did not meet AKI criteria had higher in-hospital mortality than AKI patients (AKD 17% [59/351] versus AKI 4% [19/518]; *p <* 0.001; OR 95% CI 3.10, 9.38). Mortality among dialyzed patients was 29% (Table 5). Patients with dialysis indication who could not be dialyzed had the highest mortality (43%).

## Discussion

In this feasibility study, we screened 3,577 patients in HCCs and emergency departments of 3 different countries and enrolled 2,101 patients with risk for AKI. The majority of enrolled patients had kidney dysfunction based on the first serum creatinine assessment, and 30% met criteria for AKI within 7 days. Although our loss of follow-up was high (36% of patients in the observation and 29% in the intervention phase, by 1 month), we were able to demonstrate that a high proportion of patients developed de novo CKD—170/486 (35%). Previous studies have shown that over two-thirds of AKI in LMICs occurs in the community setting, with a high frequency of AKD at presentation and with high mortality [9,13]. Our study confirmed high mortality, at 13% (273/2,101), and mortality was highest in those who had CKD (24/106 [22.6%]), followed by those with AKD (128/760 [16.8%]), AKI (85/628 [13.5%]), and NKD (36/607 [5.9%]).

In these low-resource settings, acute kidney dysfunction is seldom recognized, due to inaccessibility of diagnostic tools, limited access to healthcare, and a lack of awareness of the impact of kidney disfunction on patient outcomes [14–18]. Delays in recognizing AKI are common and have been associated with a 30% higher risk of mortality in hospitalized patients [19]. Timely recognition is a significant component of managing patients with kidney dysfunction and requires a comprehensive approach to educate caregivers on identifying patients at increased risk, addressing modifiable factors, and implementing best practices for prevention and management tailored to the local environment [20–26]. To address this issue in low-resource settings, we designed our study to assess the feasibility of identifying patients at high risk for AKI in community health centers and to test the practicality of a triaging system based on POC testing and teleconsultation for management.

One of our initial challenges was the large volume of patients seen daily in the HCCs, precluding universal screening. We applied a symptom-based risk score for AKI to identify a high-risk population for the POC creatinine test and identified kidney abnormalities in over 66% of the patients at initial presentation. The POC test and urinary dipstick results at enrollment showed that more than half of the patients had decreased eGFR and/or proteinuria, and only a small fraction had prior evidence of CKD. These findings highlight a key issue in our study, where only a minority of the patients had any data on their prior state of kidney health. In this situation, it is difficult to ascertain the chronicity of the kidney impairment and differentiate it from an evolving or recovering episode of AKI. We decided to apply the KDIGO criteria to classify these patients with “kidney impairment of unknown chronicity” as having AKD at enrollment, thus distinguishing them from the NKD group, with no renal abnormalities by POC test or urinary dipstick [27].

The stratification of patients into distinct categories of NKD, CKD, and AKD at enrollment provided an opportunity to evaluate their course and outcomes. About 30% of the enrolled patients developed AKI within 7 days, with 60% reaching KDIGO AKI stage 2 or 3, and more than 50% were hospitalized. Over 40% of AKI patients met the expanded diagnostic criterion of a decline in serum creatinine [11], and the majority were classified as having AKD at enrollment, suggesting they probably were in the resolving phase of AKI at the time of presentation. These data provide further evidence that AKI in the community may be missed if an increase in creatinine is the only criterion considered [28–31]. AKD patients on average had a worse course and worse outcomes than those with NKD. They had a higher likelihood of developing AKI, and those who did not meet AKI criteria still had a mortality rate similar to AKI patients.

Although we were unable to get complete follow-up on all patients, our findings illustrate the importance of sequential testing for renal function with POC tests and the urinary dipstick in high-risk patients in community settings [32,33]. Of patients classified as having AKD not meeting AKI criteria, and with kidney function data available after 3 months, over one-third had persistent kidney dysfunction, indicating that they may have had previously unrecognized CKD. The loss to follow-up likely led to an underestimation of the true risk of CKD. Despite having a team dedicated to arranging follow-up visits, only half of our patients could be followed up for 3 months. Some patients living in remote locations did not return to the hospital or HCC for follow-up.

We identified access to care as a potential barrier for managing patients in low-resource settings. Patients with AKD and AKI had a high risk of hospitalization, while patients with CKD were less frequently admitted. It is unclear whether this indicates that CKD patients were better managed (due to timely recognition of their kidney disease) and/or subjected to a bias of limiting care to patients who might require dialysis, which was likely unaffordable for many [26,34]. Olowu et al. [26], in a meta-analysis of studies of AKI in sub- Saharan Africa, showed that mortality exceeds 30% in both adults and pediatric patients, and increases significantly when dialysis is needed but not provided. In that study, major barriers to care and delays in treatment included out-of-pocket costs, erratic hospital resources, late presentation, and female sex. In our study, 28% of patients needing dialysis did not receive it due to lack of funding and determination of futility, and had high mortality.

Our findings comparing the observation and intervention phases support our hypothesis that a training and education program to improve recognition of AKI at HCCs and to provide telemedicine physician support both is feasible and can influence management. Although the clinical characteristics of patients in the observation and intervention phases were similar at presentation, more AKI patients and more severe AKI cases (stages 2 and 3) were identified in the intervention phase than in the observation phase. This suggests that the training and use of provided tools allowed the site staff to become more efficient in identifying at-risk cases. The initial management and disposition of patients first encountered in the HCCs was focused mainly on fluid management and triaging of patients to either home or a higher level of care. Among the known modifiable factors associated with AKI development and progression, extracellular volume depletion was likely the most common factor. In our cohort, 80% of AKI cases were associated with some degree of dehydration, and about 75% of the patients received fluid therapy, the majority with IV fluids. Our management protocol included initial fluid administration, which was utilized among patients in the intervention phase, who spent more time in the HCCs, received more fluids, and required less frequent hospitalization than those in the observation phase.

Our study has several strengths. We effectively utilized a clinical symptom-based score and deployed POC creatinine and urine dipstick tests for screening and identification of patients with kidney dysfunction across 3 different low-resource settings. We described a practical approach for evaluating and classifying patients as AKD, CKD, or NKD in the absence of knowledge of their prior state of kidney health, to guide further evaluation and follow-up. We used local mobile phone connectivity to obtain pertinent clinical data, and guided management of patients with kidney dysfunction using local resources. Our findings demonstrate that an intervention program at primary health centers in resource-constrained areas can identify and treat a high percentage of patients with AKI, and decrease the need for hospitalization in an often very distant care facility.

Our study has limitations, which potentially reflect the barriers for studies of community AKI in low-resource settings. The high number of patients seen daily at most of the HCCs made it not feasible to evaluate all patients. Thus, we were unable to determine the actual incidence of moderate to high AKI risk. Delays in patient care were frequent because some HCCs did not have regular access to intravenous fluids, and dialysis was frequently unavailable. Finally, we used technology (internet, mobile phones) to acquire data, and it may not be possible to replicate these conditions in the most remote areas with intermittent connectivity.

Our study provides evidence that there is a high prevalence of kidney disease in community health settings that can be recognized with the application of symptom-based risk assessment and POC testing. Patient management can be improved by raising awareness of AKI among healthcare workers and by incorporating teleconsultation for triaging. However, because this was a feasibility study, our sample size precluded conclusions being drawn about the effectiveness of these interventions.

Our findings also highlight some of the requirements for implementing and scaling our approach for broader application. We embedded a research coordinator at each site to enroll the patients and gather the pertinent data from the POC tests and assist the healthcare provider in implementing the study procedures. The research coordinators and healthcare workers were trained and provided with the POC tests, Android tablets, and cell phone connectivity to communicate with the teleconsultation team and record the data. Additionally, teleconsultation physicians at each center were compensated for their time. It is evident that to implement this program, health centers would need to provide the POC tests, have access to locally trained personnel and physician support at the district hospitals, and establish procedures to follow up on patients. While these barriers appear formidable, we believe they are surmountable with incremental changes in practice and reallocation of resources, especially with increasing mobile coverage and opportunities for teleconsultation and follow-up. However, we recognize that changes in healthcare policy and resource allocations are complex and varied across countries and regions.

In summary, this study shows that in low-resource settings, education of healthcare providers and provision of POC tests and sustainable mobile health technology are feasible to improve the early identification of patients with kidney dysfunction and high-risk AKI patients, and potentially improve their clinical course and outcomes. These interventions must be coupled with programs to ensure that identified patients receive all necessary treatments to prevent, minimize, and treat AKI, including dialysis, when indicated. We expect these results will spur further studies expanding our findings and will promote advocacy and action to implement early AKI recognition and response, to better patient outcomes in this frequent and deadly syndrome.

## Supporting information

S1 STROBE Checklist Checklist of items that should be included in reports of observational studies. (DOCX)

S1 Acknowledgments (DOCX)

S1 Center Characteristics Description of center characteristics from the 3 countries. (DOCX)

S1 Fig Study design and differences between the observation and intervention phases. (DOCX)

S1 Risk Score Description of the risk score utilized for patient selection. (DOCX)

S1 Protocol (PDF)

S1 STOP Protocol Protocol for management of high risk for AKI during the intervention phase. (DOCX)

S1 Table Table A: Patient characteristics and risk factors by renal function status at enrollment. Table B: Patient characteristics by country.
(DOCX)

S2 Table Screening and enrollment by center—number of patients seen in each healthcare center and proportion of screened and enrolled and AKD patients during the observation and intervention phases. (DOCX)

S3 Table Number and proportion of patients receiving fluids and diuretic therapy on enrollment day. (DOCX)

## Abbreviations

AKD: acute kidney disease
AKI: acute kidney injury
CKD: chronic kidney disease
CKD-EPI: Chronic Kidney Disease Epidemiology Collaboration
eGFR: estimated glomerular filtration rate
HCC: healthcare center
ISN: International Society of Nephrology
LMIC: low- and middle-income countries
NKD: no kidney disease
POC: point-of-care
