# Supplementary material for: Recognition and management of community-acquired acute kidney injury in low-resource settings in the ISN 0by25 trial: A multi-country feasibility study
Source: PLoS Med. 2021 Jan 14;18(1):e1003408. doi: 10.1371/journal.pmed.1003408 (PMC7808595; doi:10.1371/journal.pmed.1003408)
Supplement: S1 Acknowledgments — (DOCX) [file pmed.1003408.s002.docx]

**Supporting Information**

S1 Acknowledgments

**Bolivia**

Víctor H. García-Carlo, Maria F. Iturricha-Cáceres, Marcelo Olmos-Baya, Jheydi Condori-Saldaña and Andrea Gaspar for screening and enrolling patients in Bolivia.

**Malawi**

Naomi Sibale, Chimwemwe Mandula, Tiwonde Chinunda, Gift Sagawa,  Mickel Mollen, Enos Banda, Mwayi Mtekateka and Zuze Kawale for screening and enrolling patients in Malawi.

**Nepal**

Bhupendra Shah, Bijay Bartaula, Rina Shah, Prakriti Banjara, Pratibha Bhetwal, Chandra Shah, Badal Gajmer, Ganesh Bdr. Budathoki, Dhana Lal Ra and Mamit Rai, for screening and enrolling patients in Nepal.

**San Diego**

Ian Simpson-Shelton, Sara B Brenner for helping with the development of the educational material. Ganz Chockalingam, Ara Jermakyan, Justin Chou and Timothy Lam for developing the Android application.

**NovaBiomedical**

Jim Sidwell and Jeff Dubois for providing serum creatinine point of care devices and meters at no cost for the entire duration of the study.
