# Supplementary material for: Recognition and management of community-acquired acute kidney injury in low-resource settings in the ISN 0by25 trial: A multi-country feasibility study
Source: PLoS Med. 2021 Jan 14;18(1):e1003408. doi: 10.1371/journal.pmed.1003408 (PMC7808595; doi:10.1371/journal.pmed.1003408)
Supplement: S1 Center Characteristics — (DOCX) [file pmed.1003408.s003.docx]

**Supporting Information**

**S1 Centers Characteristics**

**Bolivia**

**Caja Nacional de Salud – Cochabamba, Bolivia**

The National Health Fund (Caja Nacional de Salud)¸ is a decentralized non-profit public institution¸ in charge of managing¸ application and execution of the Bolivian Social Security System covering the diagnosis and treatment of acute and chronic diseases¸ maternity¸ professional risks and covering the subsidies of birth, lactation and burial.

In Cochabamba, the main hospital is “Hospital Obrero No 2” this is a third level academic medical center located in the city of Cochabamba. The hospital offers both primary care and specialized services, including surgery, pediatrics, obstetrics & Gynecology, diagnosis and management of kidney diseases (with an outpatient HD unit that has 28 HD machines, with 2 CRRT devices for providing RRT at ICU, and with a PD program), infectious diseases, gastroenterology, endocrinology, neurology, orthopedics, hematology and oncology. The 360 bed hospital has 4 E.R. one for Primary Care (Internal Medicine), one for Surgery & Trauma, one for Obstetrics & Gynecology, and one for Pediatrics.

**CIMFA (Comprehensive Family Medicine Center) Quillacollo**, is a health care center of family medicine located on the city of Quillacollo (13 Km from downtown Cochabamba) that is focused in providing services that include: Helping obtaining appointments to see medical specialists, reviewing test results and notes from appointments with specialists or diagnostic and laboratory results, charting a strategy with you for managing a chronic condition, diagnosis and evaluation of unresolved health concern or staying the course to maintain good health, helping you keep current with routine examinations, immunizations and screening, and encouraging patient education and involvement in their care.

**CIS (Comprehensive Health Center) Punata** is a health care center located on the city of Punata (62 Km from downtown Cochabamba) that is focused in providing services that include: Helping obtaining appointments to see medical specialists, reviewing test results and notes from appointments with specialists or diagnostic and laboratory results, charting a strategy with you for managing a chronic condition, diagnosis and evaluation of unresolved health concern or staying the course to maintain good health, helping you keep current with routine examinations, immunizations and screening, and encouraging patient education and involvement in their care.

**CIS (Comprehensive Health Center) Sacaba** is a health care center of family medicine located on the city of Sacaba (7 Km from downtown Cochabamba) that is focused in providing services that include: Helping obtaining appointments to see medical specialists, reviewing test results and notes from appointments with specialists or diagnostic and laboratory results, charting a strategy with you for managing a chronic condition, diagnosis and evaluation of unresolved health concern or staying the course to maintain good health, helping you keep current with routine examinations, immunizations and screening, and encouraging patient education and involvement in their care.

*The CIMFA in Quillacollo and CIS in Punata and Sacaba have outpatients’ clinics of family medicine, a small room for emergencies, they do no have bed for hospitalization, only CIMFA Quillacollo has a small lab for running some blood and urine test.

**Nepal**

**B.P. Koirala Institute of Health Sciences**

B.P. Koirala Institute of Health Sciences (BPKIHS) was established on Jan 18, 1993, and subsequently upgraded as an autonomous Health Sciences University on Oct 28, 1998, with a the mandate to work towards developing socially responsible and competent health workforce, providing health care & involving in innovative health research. The Institute, located in Province No. 1, Eastern Nepal, has extended its continued health services through teaching district concept to Primary Health Care Centers, District Hospitals and Zonal Hospitals in different districts of the region.

The Institute operates a 700-bed Teaching Hospital, offering postdoctoral, postgraduate, undergraduate, and university certificate programs. The Institute grants Bachelor's, Master's, Doctoral degrees, and several other certificates. The MBBS program began in March 1994, while the postgraduate programs began in 1999. This hospital is the first hospital out of the capital city (Kathmandu) to start dialysis service and has extensive networking.

**Amda Hospital:** AMDA Hospital is located at Damak, the terai plain of Eastern Region (Province No. 1). It is 60 km east from BPKIHS, Dharan. It was established as AMDA referral center of 15-bed capacity with the help of AMDA International, and Damak Municipality in November 1992. In 1995, it was upgraded to 30 beds and started working as an implementing partner of UNHCR serving as a primary referral hospital to more than 2/3 Bhutanese Refugees in addition to the local patients. But now, AMDA Hospital provides services not only to the refugees and the local municipality but also to the people from remote districts. It has been recognized as a 75-bedded charity hospital from Ministry of Health and more than 100 beds are being used to serve the people in need. AMDA Hospital provides specialty services of Anaesthesiology, General Medicine, Surgery, Gynecology, Radiology, Pediatrics, Orthopedics, ENT, Dental, Eye, etc. This hospital has no facility of dialysis service, so patient who requires dialysis are referred to BPKIHS or other higher centers.

**Dhankuta District Hospital:** As per the National Health Policy of Nepal (1991), at least one district hospital is established in each district of the country, where out-door services, in-door services, family planning, and maternity and child health services, immunization services, and emergency services are provided. Dhankuta Hospital is one of the district hospitals of Nepal. It is located at the heart of Dhankuta, the major hilly town of Eastern Region (Province No. 1). It is 55 km north from BPKIHS, Dharan. The hospital has facilities like inpatient service, emergency services, ultrasonography and x-ray. Besides the government posted doctors, medical and dental intern doctors from BPKIHS are also posted there. This is a key referral center for primary healthcare center (PHC) and health post (HP) of the hilly region. But, there is no facility of dialysis service in this hospital, so severe patient and patient who requires dialysis are referred to BPKIHS or other higher centers. The cost of treatment is free in this hospital. This hospital has inbuilt pharmacy which provides 70 types of free medicine, which is a part of government health scheme.

**Malawi**

Malawi has a national health care service which is government funded and free for all Malawians at the point of delivery.

**Queen Elizabeth Central Hospital (QECH)** is a large tertiary referral hospital in the city of Blantyre serving the southern regions of Malawi. It is the main teaching hospital of the country as the only medical school of Malawi is located in Blantyre. Care for most medical and surgical specialities is available, but many services lack specialist doctors and most tasks are delivered by clinical officers. Basic laboratory tests, native X-rays and ultrasounds performed by radiographers can be done, but more advanced diagnostic tests are most of the time unavailable. There is a small hemodialysis unit in QECH.

**Gateway Health Centre** is an extremely busy outpatient service next to QECH for acutely unwell people. Simple point of care tests (Hb, malaria, HIV, urine dipstick) can be done, but if the patient needs any follow up, intravenous therapies or more advanced diagnostic tests, they need to be referred to QECH.

**Chileka Health Centre** is a small rural primary care centre located about 20km north from Blantyre. It provides a variety of health services, including prenatal follow up, deliveries, minor surgeries, HIV and hypertension clinics, among others. Simple point of care tests are available, but there are no laboratory or X-rays services. Patients can be admitted overnight and some intravenous medications and fluids can be administered, but patients needing further tests or longer admission need to be referred to QECH.

**Bangwe Health Centre** provides similar services with Chileka Health centre, but is busier and servers both urban and rural people close to the city of Limbe. Deliveries and minor surgeries can be done and patients can be admitted for 24 hours. However, patients needing more diagnostic tests or longer admission need to be referred to QECH.

**Chikwawa district hospital** is a small hospital in very rural part of southern Malawi. The weather is very hot compared to the other study centres and the incidence of Malaria is substantially higher. The hospital is led by a physician, but the care is delivered by clinical officers. There is an extremely busy emergency department and medical, surgical, obstetric and pediatric inpatient wards. Many operations can be performed locally by clinical officers (C-sections, laparotomies) and simple laboratory tests and X-rays are available. Patients, who need more advanced diagnostic tests, treatments (for example hemodialysis) or major surgery, need to be referred to QECH.
