# Supplementary material for: Recognition and management of community-acquired acute kidney injury in low-resource settings in the ISN 0by25 trial: A multi-country feasibility study
Source: PLoS Med. 2021 Jan 14;18(1):e1003408. doi: 10.1371/journal.pmed.1003408 (PMC7808595; doi:10.1371/journal.pmed.1003408)
Supplement: S1 Fig — (DOCX) [file pmed.1003408.s004.docx]

**Supporting Information**

**S1 Figure**– Study design and differences between observation and intervention phases.

Legend: During the enrollment study period, adult and pediatric patients with symptoms associated with AKI who presented to the local primary care facility were screened for eligibility to participate in the study. Patients with medium and high risk were consented and enrolled in the study. Consenting patients had a point-of-care (POC) test for serum creatinine (Stat Sensor Xpress Creatinine, Nova Biomedical Cooperation, Waltham, MA, USA) and a urine dipstick for albuminuria performed by the research coordinator and results communicated to the health care provider. Clinical care was provided per usual local practices. The research coordinator captured information on demographics, admission diagnosis, history, physical examination, AKI risk factors and processes of care including information about treatment, e.g., fluids and medications used. The pertinent clinical data was recorded in a mobile-enabled online secure platform accessed through computers, tablets, and mobile phones. All patients were scheduled to return for follow-up at 7 days and 1, 3, and 6 months, when serum creatinine was remeasured. In hospitalized patients, the serum creatinine POC test was repeated on day 2 after enrollment and available clinical and laboratory data were recorded daily. During the observation phase, patients were tracked throughout the healthcare evaluation, but no specific clinical guidance was provided. In the intervention phase, after enrollment teleconsultation physician provided real-time patient management guidance. Research coordinators facilitated these interactions and recorded treatment and triage decisions. Serum creatinine POC tests were repeated at day 7 and months 1, 3 and 6.

NKD: no kidney disease; AKI: acute kidney injury; AKD: acute kidney disease; CKD: chronic kidney disease; RRT: renal replacement therapy
