# Supplementary material for: Recognition and management of community-acquired acute kidney injury in low-resource settings in the ISN 0by25 trial: A multi-country feasibility study
Source: PLoS Med. 2021 Jan 14;18(1):e1003408. doi: 10.1371/journal.pmed.1003408 (PMC7808595; doi:10.1371/journal.pmed.1003408)
Supplement: S1 Risk Score — (DOCX) [file pmed.1003408.s005.docx]

**Supporting Information**

**S1 Risk Score**

Data from the International Society of Nephrology Global Snap Shot (GSN) study was used to develop a simple clinical assessment tool to predict the development of severe AKI (KDIGO stage 3), need for dialysis or mortality ^1^. The GSN utilized a mobile-enabled web-based open source database (KEEP, Distributed Health Labs, San Diego) to capture pre-specified data from patients during a physician's typical clinical day, over a 10-week enrollment period from September through December 2014. Patients who met KDIGO criteria for AKI were enrolled in the study. Data obtained included signs and symptoms at presentation to the clinic or hospital, before AKI diagnosis, as well as renal related outcomes based on serum creatinine data, need for dialysis or death. A risk score for predicting severe AKI was developed based on data from 3,283 adult patients with available outcomes. We utilized a composite outcome of death and/or need for dialysis and/or maximum KDIGO stage 3 AKI. Screening for relevant variables focused on signs and symptoms with at least 200 positive values (removed variables with few positive values to reduce model overfitting) and at least a 5% difference between outcome frequency in patients with and without the variable. Finally, we split the dataset into a training set (80% of the cohort) to build the model, and a testing set (20% of the cohort), to test the model. The risk score was based on the odds ratio (OR) estimate effect values of the regression model. In brief, we took the ratio of each estimate of the logistic regression and the lowest estimate in the logistic regression and rounded to the next integer.

Variables associated with AKI development in the univariate analysis included dehydration associated with low volume intake, diarrhea, and vomiting, decreased urine volume, hematuria, respiratory infection, fever, hypotension, shock, hemorrhage, whole body swelling, abdominal pain, loss of appetite, pallor, confusion, asthenia, and dyspnea. The comorbidities associated with the composite outcome included diabetes mellitus, liver disease, and kidney disease. HIV diagnosis was forced into the model. 50% of the patients with a score of more than 3 developed either death or severe AKI, defined as KDIGO Stage 3 or need for dialysis. The final risk score, developed using regression analysis, included the variables (list those variables with a score of 2 or more) (Table 1).

**Table 1** – Variables and points included in the risk score for predicting severe AKI.


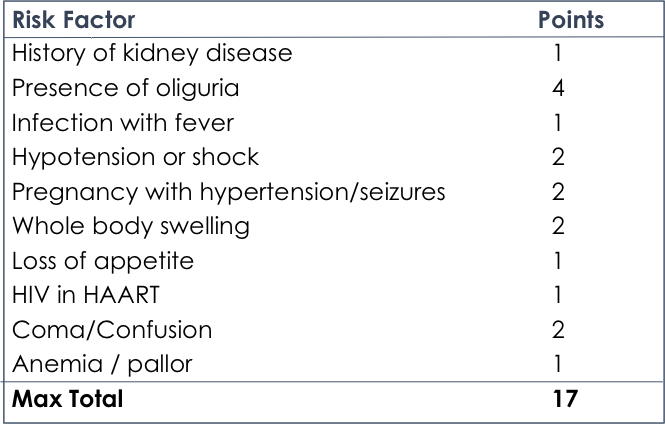


Reference

1. Mehta RL, Burdmann EA, Cerdá J, et al. Recognition and management of acute kidney injury in the International Society of Nephrology 0by25 Global Snapshot: a multinational cross-sectional study. *Lancet.* 2016.
