# Supplementary material for: Recognition and management of community-acquired acute kidney injury in low-resource settings in the ISN 0by25 trial: A multi-country feasibility study
Source: PLoS Med. 2021 Jan 14;18(1):e1003408. doi: 10.1371/journal.pmed.1003408 (PMC7808595; doi:10.1371/journal.pmed.1003408)
Supplement: S1 Protocol — (PDF) [file pmed.1003408.s006.pdf]

UC San Diego
HEALTH SCIENCES
CLINICAL AND TRANSLATIONAL
RESEARCH INSTITUTE

Statistical Analysis extra v9
CTRI Protocol 8425-2

Recognition and Management of Community-Acquired Acute Kidney Injury in Low Resource Settings –The ISN 0by25 Interventional Trial – eGFR < 60

August 6, 2020

Investigator: Ravi Mehta
Investigator: Etienne Macedo
Staff Statistician: Euyhyun Lee

1 Result

Table 1: Appendix Table 2

	Overall ($n = 2101$)	Observation ($n = 978$)	Intervention ($n = 1123$)	p-value
**Mortality at discharge - Hospitalized patients**	105/1126 (9.3%)	38/578 (6.6%)	67/548 (12.2%)	0.001**
NKD	17/208 (8.2%)	7/125 (5.6%)	10/83 (12.0%)	0.122
No AKI	86/608 (14.1%)	34/367 (9.3%)	52/241 (21.6%)	<0.001***
AKI	19/518 (3.7%)	4/211 (1.9%)	15/307 (4.9%)	0.096
stage 1	5/191 (2.6%)	1/73 (1.4%)	4/118 (3.4%)	0.651
stage 2	3/116 (2.6%)	1/41 (2.4%)	2/75 (2.7%)	1.000
stage 3	11/211 (5.2%)	2/97 (2.1%)	9/114 (7.9%)	0.068
severe AKI (stage2/3)	14/327 (4.3%)	3/138 (2.2%)	11/189 (5.8%)	0.165
Dialysis indication but not dialyzed	19/45 (42.2%)	5/15 (33.3%)	14/30 (46.7%)	0.526
Dialyzed	2/58 (3.4%)	1/31 (3.2%)	1/27 (3.7%)	1.000
AKD	59/351 (16.8%)	23/208 (11.1%)	36/143 (25.2%)	<0.001***
CKD	10/49 (20.4%)	4/34 (11.8%)	6/15 (40.0%)	0.049*
**Mortality during follow up - Hospitalized patients**	112/1021 (11.0%)	67/540 (12.4%)	45/481 (9.4%)	0.133
NKD	10/191 (5.2%)	6/118 (5.1%)	4/73 (5.5%)	1.000
No AKI	51/522 (9.8%)	35/333 (10.5%)	16/189 (8.5%)	0.540
AKI	61/499 (12.2%)	32/207 (15.5%)	29/292 (9.9%)	0.072
stage 1	13/186 (7.0%)	5/72 (6.9%)	8/114 (7.0%)	1.000
stage 2	17/113 (15.0%)	10/40 (25.0%)	7/73 (9.6%)	0.051
stage 3	31/200 (15.5%)	17/95 (17.9%)	14/105 (13.3%)	0.436
severe AKI (stage2/3)	48/313 (15.3%)	27/135 (20.0%)	21/178 (11.8%)	0.057
Dialysis indication but not dialyzed	6/26 (23.1%)	2/10 (20.0%)	4/16 (25.0%)	1.000
Dialyzed	15/56 (26.8%)	6/30 (20.0%)	9/26 (34.6%)	0.243
AKD	37/292 (12.7%)	26/185 (14.1%)	11/107 (10.3%)	0.465
CKD	4/39 (10.3%)	3/30 (10.0%)	1/9 (11.1%)	1.000
**Mortality during follow up - Non-hospitalized patients**	56/975 (5.7%)	19/400 (4.8%)	37/575 (6.4%)	0.327
NKD	9/399 (2.3%)	4/188 (2.1%)	5/211 (2.4%)	1.000
No AKI	51/865 (5.9%)	17/371 (4.6%)	34/494 (6.9%)	0.189
AKI	5/110 (4.5%)	2/29 (6.9%)	3/81 (3.7%)	0.606
stage 1	3/61 (4.9%)	2/18 (11.1%)	1/43 (2.3%)	0.205
stage 2	0/22 (0.0%)	0/5 (0.0%)	0/17 (0.0%)	1.000
stage 3	2/27 (7.4%)	0/6 (0.0%)	2/21 (9.5%)	1.000
severe AKI (stage2/3)	2/49 (4.1%)	0/11 (0.0%)	2/38 (5.3%)	1.000
Dialysis indication but not dialyzed	4/22 (18.2%)	1/7 (14.3%)	3/15 (20.0%)	1.000
Dialyzed	1/5 (20.0%)	0/1 (0.0%)	1/4 (25.0%)	1.000
AKD	32/409 (7.8%)	12/167 (7.2%)	20/242 (8.3%)	0.852
CKD	10/57 (17.5%)	1/16 (6.2%)	9/41 (22.0%)	0.253
**Overall Mortality**	273/2101 (13.0%)	124/978 (12.7%)	149/1123 (13.3%)	0.697
NKD	36/607 (5.9%)	17/313 (5.4%)	19/294 (6.5%)	0.610
No AKI	188/1473 (12.8%)	86/738 (11.7%)	102/735 (13.9%)	0.212
AKI	85/628 (13.5%)	38/240 (15.8%)	47/388 (12.1%)	0.189
stage 1	21/252 (8.3%)	8/91 (8.8%)	13/161 (8.1%)	0.817
stage 2	20/138 (14.5%)	11/46 (23.9%)	9/92 (9.8%)	0.039*
stage 3	44/238 (18.5%)	19/103 (18.4%)	25/135 (18.5%)	1.000
severe AKI (stage2/3)	64/376 (17.0%)	30/149 (20.1%)	34/227 (15.0%)	0.208
Dialysis indication but not dialyzed	29/67 (43.3%)	8/22 (36.4%)	21/45 (46.7%)	0.447
Dialyzed	18/63 (28.6%)	7/32 (21.9%)	11/31 (35.5%)	0.274
AKD	128/760 (16.8%)	61/375 (16.3%)	67/385 (17.4%)	0.699
CKD	24/106 (22.6%)	8/50 (16.0%)	16/56 (28.6%)	0.164

Table 2: Table 2 Age Section

	Overall ($n = 2101$)	Observation ($n = 978$)	Intervention ($n = 1123$)	p-value
Children (<18)	272 (13.0%)	130 (13.3%)	142 (12.7%)	0.696
Mean Age	7.93 (5.69)	8.25 (5.35)	7.64 (5.98)	0.376
Adult	1825 (87.0%)	848 (86.7%)	977 (87.3%)	0.696
Mean Age	48.49 (18.06)	49.04 (18.35)	48.02 (17.80)	0.230

Table 3: Table 4

	Overall ($n = 2101$)	Observation ($n = 978$)	Intervention ($n = 1123$)	p-value
**Renal Function at admission**				
CKD	197 (9.4%)	80 (8.2%)	117 (10.4%)	0.094
With albuminuria	66 (3.9%)	39 (5.1%)	27 (2.9%)	
AKD	1199 (57.1%)	552 (56.4%)	647 (57.6%)	
Based on sCr alone	789 (65.8%)	338 (61.2%)	451 (69.7%)	
Based on sCr and albuminuria	218 (18.2%)	95 (17.2%)	123 (19.0%)	
Based on albuminuria alone	192 (16.0%)	119 (21.6%)	73 (11.3%)	
NKD	705 (33.6%)	346 (35.4%)	359 (32.0%)	
**Renal Fuction at 7 days**				
No AKI	1473 (70.1%)	738 (75.5%)	735 (65.4%)	<0.001
CKD	106 (53.8%)	50 (62.5%)	56 (47.9%)	0.058
AKD	760 (63.4%)	375 (67.9%)	385 (59.5%)	0.003
NKD	607 (86.1%)	313 (90.5%)	294 (81.9%)	0.001
AKI	628 (29.9%)	240 (24.5%)	388 (34.6%)	<0.001
Stage 1	252 (40.1%)	91 (37.9%)	161 (41.5%)	0.403
Stage 2	138 (22.0%)	46 (19.2%)	92 (23.7%)	0.198
Stage 3	238 (37.9%)	103 (42.9%)	135 (34.8%)	0.043
severe AKI (stage2/3)	376 (59.9%)	149 (62.1%)	227 (58.5%)	0.403
Disposition				
Sent home	975 (46.4%)	400 (40.9%)	575 (51.2%)	<0.001
Admitted	1126 (53.6%)	578 (59.1%)	548 (48.8%)	
NKD	208 (18.5%)	125 (21.6%)	83 (15.1%)	0.006
AKI	518 (46.0%)	211 (36.5%)	307 (56.0%)	<0.001
AKD	351 (31.2%)	208 (36.0%)	143 (26.1%)	<0.001
CKD	49 (4.4%)	34 (5.9%)	15 (2.7%)	0.012
Time(hours) in health care facility in all patients	28.69 (4.25 - 120.00)	27.00 (3.52 - 120.78)	31.00 (4.85 - 118.00)	0.243
Sent home	3.80 (1.00 - 6.07)	1.56 (0.21 - 5.53)	4.00 (2.00 - 6.48)	<0.001
Admitted	96.00 (46.70 - 167.13)	95.05 (29.08 - 177.35)	97.75 (49.92 - 158.95)	0.167
**Dialysis Requirement**				
Dialysis Indiction	130 (6.2%)	54 (5.5%)	76 (6.8%)	0.276
Dialyzed	63 (3.0%)	32 (3.3%)	31 (2.8%)	0.523
AKD	14 (22.2%)	11 (34.4%)	3 (9.7%)	0.027
AKI	43 (68.3%)	20 (62.5%)	23 (74.2%)	
CKD	6 (9.5%)	1 (3.1%)	5 (16.1%)	
Dialysis indication, but not dialyzed	67 (3.2%)	22 (2.2%)	45 (4.0%)	0.025
AKD	31 (46.3%)	11 (50.0%)	20 (44.4%)	0.768
AKI	21 (31.3%)	5 (22.7%)	16 (35.6%)	
CKD	12 (17.9%)	5 (22.7%)	7 (15.6%)	
NKD	3 (4.5%)	1 (4.5%)	2 (4.4%)	

Table 4: Table 6

	Overall	Observation	Intervention	p-value
**Renal Recovery At 3 months**				
Overall total recovery	344/425 (80.9%)	119/152 (78.3%)	225/273 (82.4%)	0.305
New onset AKD on NKD	5/140 (3.6%)	2/54 (3.7%)	3/86 (3.5%)	1.000
CKD progression	5/10 (50.0%)	2/4 (50.0%)	3/6 (50.0%)	1.000
New onset CKD in AKD without AKI	38/115 (33.0%)	19/45 (42.2%)	19/70 (27.1%)	0.107
New onset CKD in AKI	28/160 (17.5%)	8/49 (16.3%)	20/111 (18.0%)	1.000
**At 6 months**				
Overall total recovery	189/235 (80.4%)	73/98 (74.5%)	116/137 (84.7%)	0.066
New onset AKD on NKD	4/89 (4.5%)	2/39 (5.1%)	2/50 (4.0%)	1.000
CKD progression	4/5 (80.0%)	1/2 (50.0%)	3/3 (100.0%)	0.400
New onset CKD in AKD without AKI	24/68 (35.3%)	14/32 (43.8%)	10/36 (27.8%)	0.208
New onset CKD in AKI	13/73 (17.8%)	7/25 (28.0%)	6/48 (12.5%)	0.118
**Overall**				
Overall total recovery	398/495 (80.4%)	145/190 (76.3%)	253/305 (83.0%)	0.081
New onset AKD on NKD	7/172 (4.1%)	4/72 (5.6%)	3/100 (3.0%)	0.454
CKD progression	6/11 (54.5%)	2/4 (50.0%)	4/7 (57.1%)	1.000
New onset CKD in AKD without AKI	47/138 (34.1%)	25/61 (41.0%)	22/77 (28.6%)	0.149
New onset CKD in AKI	32/174 (18.4%)	12/53 (22.6%)	20/121 (16.5%)	0.396

Table 5: Table 5

		Overall	Observation	Intervention	P
Any fluid - all patients		1588 (75.6%)	714 (73.0%)	874 (77.8%)	0.012*
		$n = 637$	$n = 322$	$n = 315$	
	volume	1600.00 (1130.00 - 3000.00)	1200.00 (900.00 - 1600.00)	2500.00 (1725.00 - 3500.00)	<0.001***
Sent home		598 (61.3%)	220 (55.0%)	378 (65.7%)	
		$n = 239$	$n = 79$	$n = 160$	
	volume	2000.00 (1200.00 - 3000.00)	1200.00 (900.00 - 1600.00)	2500.00 (1500.00 - 3037.50)	<0.001***
Admitted		990 (87.9%)	494 (85.5%)	496 (90.5%)	
		$n = 398$	$n = 243$	$n = 155$	
	volume	1500.00 (1100.00 - 2500.00)	1200.00 (850.00 - 1575.00)	2500.00 (2000.00 - 3500.00)	<0.001***
IV fluid		1377 (69.7%)	592 (62.2%)	785 (76.6%)	<0.001***
		$n = 1186$	$n = 494$	$n = 692$	
	volume	1000.00 (1000.00 - 2000.00)	1000.00 (500.00 - 1000.00)	1500.00 (1000.00 - 2000.00)	<0.001***
Sent home		469 (55.1%)	140 (37.4%)	329 (69.0%)	
		$n = 449$	$n = 138$	$n = 311$	
	volume	1000.00 (1000.00 - 1500.00)	1000.00 (500.00 - 1000.00)	1000.00 (1000.00 - 2000.00)	<0.001***
Admitted		908 (80.6%)	452 (78.2%)	456 (83.2%)	
		$n = 737$	$n = 356$	$n = 381$	
	volume	1000.00 (1000.00 - 2000.00)	1000.00 (500.00 - 1200.00)	1500.00 (1000.00 - 2000.00)	<0.001***
PO fluid		813 (38.7%)	421 (43.0%)	392 (34.9%)	<0.001***
	volume	600.00 (300.00 - 1000.00)	350.00 (200.00 - 600.00)	1000.00 (500.00 - 1500.00)	<0.001***
Sent home		316 (32.4%)	108 (27.0%)	208 (36.2%)	
	volume	1000.00 (400.00 - 1500.00)	475.00 (200.00 - 1000.00)	1000.00 (500.00 - 1962.50)	<0.001***
Admitted		497 (44.1%)	313 (54.2%)	184 (33.6%)	
	volume	500.00 (200.00 - 1000.00)	350.00 (200.00 - 550.00)	1000.00 (775.00 - 1200.00)	<0.001***
Diuretic use					
		170 (8.6%)	86 (9.0%)	84 (8.2%)	0.559

Table 6: Follow up at each time point

	Observation	Intervention	Overall	p-value
follow up 7d	$n = 978$	$n = 1123$	$n = 2101$	
0	143 (14.6%)	204 (18.2%)	347 (16.5%)	0.034*
1	835 (85.4%)	919 (81.8%)	1754 (83.5%)	
follow up 1m	$n = 978$	$n = 1123$	$n = 2101$	
0	352 (36.0%)	325 (28.9%)	677 (32.2%)	<0.001***
1	626 (64.0%)	798 (71.1%)	1424 (67.8%)	
follow up 3m	$n = 978$	$n = 1123$	$n = 2101$	
0	583 (59.6%)	549 (48.9%)	1132 (53.9%)	<0.001***
1	395 (40.4%)	574 (51.1%)	969 (46.1%)	
follow up 6m	$n = 978$	$n = 1123$	$n = 2101$	
0	684 (69.9%)	910 (81.0%)	1594 (75.9%)	<0.001***
1	294 (30.1%)	213 (19.0%)	507 (24.1%)	
Mortality by 7days	$n = 952$	$n = 1029$	$n = 1981$	
0	886 (90.6%)	939 (83.6%)	1825 (86.9%)	0.157
1	66 (6.7%)	90 (8.0%)	156 (7.4%)	
NA	26 (2.7%)	94 (8.4%)	120 (5.7%)	
Mortality by 1month	$n = 952$	$n = 1031$	$n = 1983$	
0	868 (88.8%)	909 (80.9%)	1777 (84.6%)	0.034*
1	84 (8.6%)	122 (10.9%)	206 (9.8%)	
NA	26 (2.7%)	92 (8.2%)	118 (5.6%)	
Mortality by 3months	$n = 952$	$n = 1031$	$n = 1983$	
0	836 (85.5%)	889 (79.2%)	1725 (82.1%)	0.325
1	116 (11.9%)	142 (12.6%)	258 (12.3%)	
NA	26 (2.7%)	92 (8.2%)	118 (5.6%)	
Mortality by 6months	$n = 952$	$n = 1031$	$n = 1983$	
0	828 (84.7%)	882 (78.5%)	1710 (81.4%)	0.392
1	124 (12.7%)	149 (13.3%)	273 (13.0%)	
NA	26 (2.7%)	92 (8.2%)	118 (5.6%)	
mortality at discharge	$n = 578$	$n = 548$	$n = 1126$	
Yes	38 (6.6%)	67 (12.2%)	105 (9.3%)	0.002**
No	540 (93.4%)	481 (87.8%)	1021 (90.7%)	
Mortality at 7days	$n = 914$	$n = 962$	$n = 1876$	
0	886 (90.6%)	939 (83.6%)	1825 (86.9%)	0.451
1	28 (2.9%)	23 (2.0%)	51 (2.4%)	
NA	64 (6.5%)	161 (14.3%)	225 (10.7%)	
Mortality at 1month	$n = 886$	$n = 941$	$n = 1827$	
0	868 (88.8%)	909 (80.9%)	1777 (84.6%)	0.099
1	18 (1.8%)	32 (2.8%)	50 (2.4%)	
NA	92 (9.4%)	182 (16.2%)	274 (13.0%)	
Mortalityat 3months	$n = 868$	$n = 909$	$n = 1777$	
0	836 (85.5%)	889 (79.2%)	1725 (82.1%)	0.086
1	32 (3.3%)	20 (1.8%)	52 (2.5%)	
NA	110 (11.2%)	214 (19.1%)	324 (15.4%)	
Mortality at 6months	$n = 836$	$n = 889$	$n = 1725$	
0	828 (84.7%)	882 (78.5%)	1710 (81.4%)	0.905
1	8 (0.8%)	7 (0.6%)	15 (0.7%)	
NA	142 (14.5%)	234 (20.8%)	376 (17.9%)	

Table 7: table 6 - de novo CKD

	Overall	Observation	Intervention	p-value
**Renal Recovery At 3 months**				
De novo CKD	66/415 (15.9%)	27/148 (18.2%)	39/267 (14.6%)	0.331
**At 6 months**				
De novo CKD	37/230 (16.1%)	21/96 (21.9%)	16/134 (11.9%)	0.047*
**Overall**				
De novo CKD	79/484 (16.3%)	37/186 (19.9%)	42/298 (14.1%)	0.101

Table 8: Mortality - NKD vs. other

	NKD ($n = 607$)	Other ($n = 1494$)	Overall ($n = 2101$)	p-value
Mortality Overall				
Yes	36 (5.9%)	237 (15.9%)	273 (13.0%)	<0.001***
No	571 (94.1%)	1257 (84.1%)	1828 (87.0%)	

Figure 1:

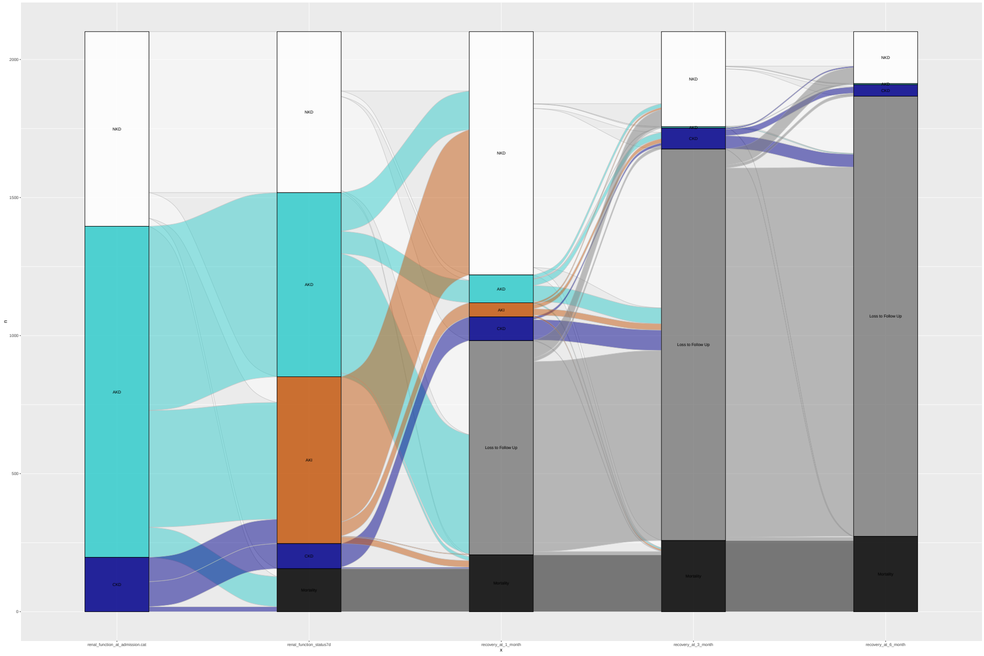

Table 9: Raw groupings of the new variables

	Overall	Observation	Intervention
renal function at admission.cat	$n = 2101$	$n = 978$	$n = 1123$
AKD	1199 (57.1%)	552 (56.4%)	647 (57.6%)
CKD	197 (9.4%)	80 (8.2%)	117 (10.4%)
NKD	705 (33.6%)	346 (35.4%)	359 (32.0%)
renal function status7d	$n = 2101$	$n = 978$	$n = 1123$
AKD	760 (36.2%)	375 (38.3%)	385 (34.3%)
AKI	628 (29.9%)	240 (24.5%)	388 (34.6%)
CKD	106 (5.0%)	50 (5.1%)	56 (5.0%)
NKD	607 (28.9%)	313 (32.0%)	294 (26.2%)
recovery at 1 month	$n = 1189$	$n = 515$	$n = 674$
AKD	83 (4.0%)	35 (3.6%)	48 (4.3%)
AKI	62 (3.0%)	23 (2.4%)	39 (3.5%)
CKD	106 (5.0%)	50 (5.1%)	56 (5.0%)
New onset AKD	20 (1.0%)	12 (1.2%)	8 (0.7%)
NKD	215 (10.2%)	109 (11.1%)	106 (9.4%)
Recovery from AKD	141 (6.7%)	69 (7.1%)	72 (6.4%)
Recovery from AKI	562 (26.7%)	217 (22.2%)	345 (30.7%)
NA	912 (43.4%)	463 (47.3%)	449 (40.0%)
recovery at 3 month	$n = 890$	$n = 343$	$n = 547$
AKI	28 (1.3%)	8 (0.8%)	20 (1.8%)
CKD progression	5 (0.2%)	2 (0.2%)	3 (0.3%)
New onset AKD	5 (0.2%)	2 (0.2%)	3 (0.3%)
New onset CKD	38 (1.8%)	19 (1.9%)	19 (1.7%)
NKD	135 (6.4%)	52 (5.3%)	83 (7.4%)
Recovery from AKD	78 (3.7%)	26 (2.7%)	52 (4.6%)
Recovery from AKI	596 (28.4%)	232 (23.7%)	364 (32.4%)
Stable CKD	5 (0.2%)	2 (0.2%)	3 (0.3%)
NA	1211 (57.6%)	635 (64.9%)	576 (51.3%)
recovery at 6 month	$n = 786$	$n = 313$	$n = 473$
AKI	13 (0.6%)	7 (0.7%)	6 (0.5%)
CKD progression	4 (0.2%)	1 (0.1%)	3 (0.3%)
New onset AKD	4 (0.2%)	2 (0.2%)	2 (0.2%)
New onset CKD	24 (1.1%)	14 (1.4%)	10 (0.9%)
NKD	85 (4.0%)	37 (3.8%)	48 (4.3%)
Recovery from AKD	44 (2.1%)	18 (1.8%)	26 (2.3%)
Recovery from AKI	611 (29.1%)	233 (23.8%)	378 (33.7%)
Stable CKD	1 (0.0%)	1 (0.1%)	0 (0.0%)
NA	1315 (62.6%)	665 (68.0%)	650 (57.9%)

Figure 2:

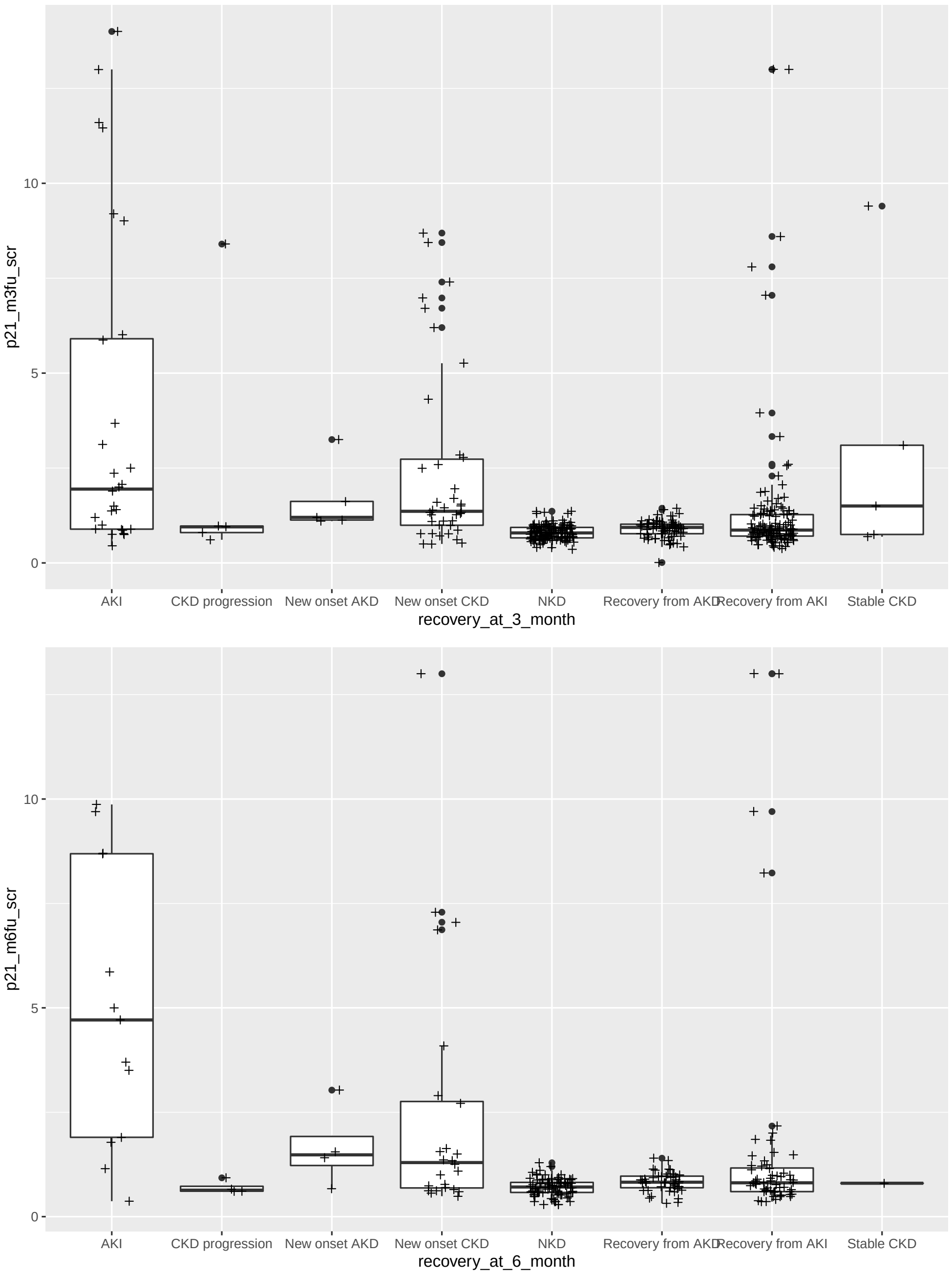
p21_m3fu_scr
0
5
10
AKI
CKD progression
New onset AKD
New onset CKD
NKD
Recovery from AKD
Recovery from AKI
Stable CKD
recovery_at_3_month
p21_m6fu_scr
0
5
10
AKI
CKD progression
New onset AKD
New onset CKD
NKD
Recovery from AKD
Recovery from AKI
Stable CKD
recovery_at_6_month

Table 10: Patient characteristics and risk factors by renal function status at enrollment.

	AKD	CKD	NKD	Overall	p-value
**Age**	$n = 1199$	$n = 197$	$n = 701$	$n = 2097$	
	43.98 (22.15)	54.99 (23.52)	38.65 (19.06)	43.23 (21.77)	<0.001***
**Adult**	$n = 1199$	$n = 197$	$n = 701$	$n = 2097$	
Yes	1031 (86.0%)	173 (87.8%)	621 (88.6%)	1825 (87.0%)	0.251
**Female**	$n = 1199$	$n = 197$	$n = 705$	$n = 2101$	
Yes	612 (51.0%)	100 (50.8%)	405 (57.4%)	1117 (53.2%)	0.020*
**Race**	$n = 1198$	$n = 197$	$n = 704$	$n = 2099$	
african	451 (37.6%)	26 (13.2%)	336 (47.7%)	813 (38.7%)	<0.001***
asian	615 (51.3%)	106 (53.8%)	167 (23.7%)	888 (42.3%)	
aymara	8 (0.7%)	4 (2.0%)	3 (0.4%)	15 (0.7%)	
hispanic	72 (6.0%)	39 (19.8%)	122 (17.3%)	233 (11.1%)	
middle eastern	0 (0.0%)	0 (0.0%)	1 (0.1%)	1 (0.0%)	
quechua	52 (4.3%)	22 (11.2%)	75 (10.7%)	149 (7.1%)	
**DM**	$n = 1199$	$n = 197$	$n = 705$	$n = 2101$	
Yes	146 (12.2%)	63 (32.0%)	47 (6.7%)	256 (12.2%)	<0.001***
**Liver**	$n = 1199$	$n = 197$	$n = 705$	$n = 2101$	
Yes	65 (5.4%)	11 (5.6%)	11 (1.6%)	87 (4.1%)	<0.001***
**Heart**	$n = 1199$	$n = 197$	$n = 705$	$n = 2101$	
Yes	39 (3.3%)	29 (14.7%)	20 (2.8%)	88 (4.2%)	<0.001***
**Lung**	$n = 1199$	$n = 197$	$n = 705$	$n = 2101$	
Yes	61 (5.1%)	19 (9.6%)	39 (5.5%)	119 (5.7%)	0.037*
**HIV**	$n = 1199$	$n = 197$	$n = 705$	$n = 2101$	
Yes	222 (18.5%)	6 (3.0%)	145 (20.6%)	373 (17.8%)	<0.001***
**Anemia**	$n = 1199$	$n = 197$	$n = 705$	$n = 2101$	
Yes	149 (12.4%)	43 (21.8%)	63 (8.9%)	255 (12.1%)	<0.001***
**Cancer**	$n = 1199$	$n = 197$	$n = 705$	$n = 2101$	
Yes	20 (1.7%)	5 (2.5%)	11 (1.6%)	36 (1.7%)	0.635
**Hypertension**	$n = 1199$	$n = 197$	$n = 705$	$n = 2101$	
Yes	254 (21.2%)	97 (49.2%)	67 (9.5%)	418 (19.9%)	<0.001***
**Dehydration**	$n = 1199$	$n = 197$	$n = 705$	$n = 2101$	
Yes	902 (75.2%)	147 (74.6%)	484 (68.7%)	1533 (73.0%)	0.007**
**Dehydration: Diarrhea**	$n = 1199$	$n = 197$	$n = 705$	$n = 2101$	
Yes	335 (27.9%)	50 (25.4%)	215 (30.5%)	600 (28.6%)	0.287
**Dehydration: Vomiting**	$n = 1199$	$n = 197$	$n = 705$	$n = 2101$	
Yes	626 (52.2%)	95 (48.2%)	329 (46.7%)	1050 (50.0%)	0.057
**Dehydration: Sweating**	$n = 1199$	$n = 197$	$n = 705$	$n = 2101$	
Yes	78 (6.5%)	11 (5.6%)	70 (9.9%)	159 (7.6%)	0.013*
**Dehydration: Thirst**	$n = 1199$	$n = 197$	$n = 705$	$n = 2101$	
Yes	170 (14.2%)	36 (18.3%)	190 (27.0%)	396 (18.8%)	<0.001***
**Dehydration: Low intake**	$n = 1199$	$n = 197$	$n = 705$	$n = 2101$	
Yes	615 (51.3%)	112 (56.9%)	294 (41.7%)	1021 (48.6%)	<0.001***
**Weakness**	$n = 1199$	$n = 197$	$n = 705$	$n = 2101$	
Yes	1098 (91.6%)	178 (90.4%)	638 (90.5%)	1914 (91.1%)	0.675
**Urinary**	$n = 1199$	$n = 197$	$n = 705$	$n = 2101$	
Yes	661 (55.1%)	125 (63.5%)	381 (54.0%)	1167 (55.5%)	0.057
**Urinary: Oliguria**	$n = 1199$	$n = 197$	$n = 705$	$n = 2101$	
Yes	399 (33.3%)	91 (46.2%)	258 (36.6%)	748 (35.6%)	0.002**
**Urinary: Polyuria**	$n = 1199$	$n = 197$	$n = 705$	$n = 2101$	
Yes	53 (4.4%)	4 (2.0%)	39 (5.5%)	96 (4.6%)	0.107

Table 11: Patient characteristics and risk factors by renal function status at enrollment.

	AKD	CKD	NKD	Overall	p-value
**Urinary: Dysuria**	$n = 1199$	$n = 197$	$n = 705$	$n = 2101$	
Yes	248 (20.7%)	36 (18.3%)	148 (21.0%)	432 (20.6%)	0.697
**Urinary: Hematuria**	$n = 1199$	$n = 197$	$n = 705$	$n = 2101$	
Yes	50 (4.2%)	10 (5.1%)	36 (5.1%)	96 (4.6%)	0.600
**Infection**	$n = 1199$	$n = 197$	$n = 705$	$n = 2101$	
Yes	868 (72.4%)	111 (56.3%)	537 (76.2%)	1516 (72.2%)	<0.001***
**Infection: Malaria**	$n = 1199$	$n = 197$	$n = 705$	$n = 2101$	
Yes	64 (5.3%)	2 (1.0%)	42 (6.0%)	108 (5.1%)	0.019*
**Infection: GI**	$n = 1199$	$n = 197$	$n = 705$	$n = 2101$	
Yes	206 (17.2%)	28 (14.2%)	176 (25.0%)	410 (19.5%)	<0.001***
**Hypotension**	$n = 1199$	$n = 197$	$n = 705$	$n = 2101$	
Yes	285 (23.8%)	38 (19.3%)	136 (19.3%)	459 (21.8%)	0.049*
**Swelling: Whole**	$n = 1199$	$n = 197$	$n = 705$	$n = 2101$	
Yes	92 (7.7%)	39 (19.8%)	31 (4.4%)	162 (7.7%)	<0.001***
**Swelling: Face**	$n = 1199$	$n = 197$	$n = 705$	$n = 2101$	
Yes	100 (8.3%)	15 (7.6%)	40 (5.7%)	155 (7.4%)	0.098
**Swelling: Lower**	$n = 1199$	$n = 197$	$n = 705$	$n = 2101$	
Yes	294 (24.5%)	50 (25.4%)	105 (14.9%)	449 (21.4%)	<0.001***
**Asthenia**	$n = 1199$	$n = 197$	$n = 705$	$n = 2101$	
Yes	175 (14.6%)	39 (19.8%)	167 (23.7%)	381 (18.1%)	<0.001***
**Dyspnea**	$n = 1199$	$n = 197$	$n = 705$	$n = 2101$	
Yes	209 (17.4%)	50 (25.4%)	76 (10.8%)	335 (15.9%)	<0.001***
**Weight loss**	$n = 1199$	$n = 197$	$n = 705$	$n = 2101$	
Yes	251 (20.9%)	34 (17.3%)	143 (20.3%)	428 (20.4%)	0.493
**loss of appetite**	$n = 1199$	$n = 197$	$n = 705$	$n = 2101$	
Yes	789 (65.8%)	112 (56.9%)	464 (65.8%)	1365 (65.0%)	0.043*
**Pallor**	$n = 1199$	$n = 197$	$n = 705$	$n = 2101$	
Yes	218 (18.2%)	47 (23.9%)	128 (18.2%)	393 (18.7%)	0.150
**ACEI**	$n = 1199$	$n = 197$	$n = 705$	$n = 2101$	
Yes	151 (12.6%)	61 (31.0%)	40 (5.7%)	252 (12.0%)	<0.001***
**NSAIDS**	$n = 1199$	$n = 197$	$n = 705$	$n = 2101$	
Yes	154 (12.8%)	35 (17.8%)	112 (15.9%)	301 (14.3%)	0.066
**Tenofovir**	$n = 1199$	$n = 197$	$n = 705$	$n = 2101$	
Yes	193 (16.1%)	6 (3.0%)	125 (17.7%)	324 (15.4%)	<0.001***

Table 12: Change in renal function status from enrollment to 7 days

	AKD ($n = 1199$)	CKD ($n = 197$)	NKD ($n = 705$)	Overall ($n = 2101$)
renal function status7d				
AKD	760 (63.4%)	0 (0.0%)	0 (0.0%)	760 (36.2%)
AKI	439 (36.6%)	91 (46.2%)	98 (13.9%)	628 (29.9%)
CKD	0 (0.0%)	106 (53.8%)	0 (0.0%)	106 (5.0%)
NKD	0 (0.0%)	0 (0.0%)	607 (86.1%)	607 (28.9%)

Table 13: Criteria for AKI at 7 days

	AKD ($n = 439$)	CKD ($n = 91$)	NKD ($n = 98$)
realAKI 48 7days			
0	207 (47.2%)	30 (33.0%)	24 (24.5%)
1	232 (52.8%)	61 (67.0%)	74 (75.5%)
decreaseAKI 48 7days.pure			
0	232 (52.8%)	61 (67.0%)	74 (75.5%)
1	207 (47.2%)	30 (33.0%)	24 (24.5%)

2 Appendix

2.1 Changes

The following changes were made to the data sets:

- 1/14/20
  - **recovery_at_3month** and **recovery_at_6month** have been reclassified to use the correct scr value (**p21_m3fu_scr** and **p21_m6fu_scr**)
  - **recovery_at_1month** was reclassified also.

Based on the new definition of gfr < 60, these patients were shown to recover from CKD.

	patient_id	recovery_at_3_month	recovery_at_6_month	sCr3month	sCr6month
148	BV3049	AKI	Recovery from AKI	1	1
731	IMW5057	AKI	Recovery from AKI	1	1
1022	INP4071	AKI	Recovery from AKI	1	1
1042	INP4094	New onset CKD	Recovery from AKD	1	1
2011	NP5159	AKI	Recovery from AKI	1	1

2.2 R Code

```
library(Hmisc)
library(plyr)
library(dplyr)
library(readxl)
library(magrittr)
library(pROC)
library(lubridate)
library(xtable)
library(randomForest)
library(ROCR)
library(nnet)
library(rpart)
library(car)
library(glmnet)
library(caret)
#library(MKmisc)
library(rms)
library(scales)
library(diagram)
library(stringr)
library(stringi)
options(digit = 3, stringsAsFactors = FALSE, width = 100)
source('/Users/e4lee/Desktop/CTRI/CTRI_Functions.R')
setwd ('/Users/e4lee/Desktop/CTRI/Projects/8425_Mehta_2/Project 2/Analysis Plan/')


RE.Names <- function(x){
  dtNames <- tools::toTitleCase(tolower(gsubVec(c('\\_', '\\.'),c(' ', ' '), x)))
  dtNames <- gsubVec(c('Hops', 'Dm', 'Cad', 'Mi', 'Aki', 'Icd9', 'Hiv', 'Dg', 'Ckd', 'dm', 'Esrd', 'Mlophaty',
                       'Poisonig', 'Be ','Acei', 'Arb', 'Cni', 'Lmwh', 'Gk', 'k at', ' b', 'Gaki', 'New ', 'Age c', 'k
                            c'),
                     c('', 'DM', 'CAD', 'MI', 'AKI', 'ICD9', 'HIV', '', 'CKD', 'DM', 'ESRD', 'Miophaty',
                       'Poisoning', '', 'ACEI', 'ARB', 'CNI', 'LMWH', 'GK', 'Potassium Level at', ' B', 'gAKI', '', '
                            Age Categorized',
                       'K Categorized'),
                     dtNames, fixed = T)
  dtNames
}


##############################################
## Preliminaries                            ##
##############################################
#dat1 <- read_xlsx('/Users/e4lee/Desktop/CTRI/Projects/8425_Mehta_2/Project 2/Data/table for Euy 7feb2018.xlsx', sheet
      = 1)
dat2 <- read_xlsx('/Users/e4lee/Desktop/CTRI/Projects/8425_Mehta_2/Project 2/Data/01_11_20/table for Euy 2020 01 08.
     xlsx', sheet = 1)
dat2 <- data.frame(dat2)
dat <- dplyr::filter(dat2, p01_enrolled == 1, noscr == 0)
dat.o <- dat
dat$phase.cat <- factor(gsubVec(c(1, 2), c('Observation', 'Intervention'), dat$phase), levels = c('Observation', '
     Intervention'))


####### New data set modification ###########
## Renal function based on < 60 is now used
## The output is located in renal_function _*time*2 columns
## Reassigned column names to make the code run

dat[which(dat$renal_function_status7d2 == 2 & dat$p21_m1fu_scr <= dat$p02_scr_us), 'recovery_at_1_month']  <- 1
dat[which(dat$renal_function_status7d2 == 2 & dat$p21_m1fu_scr > dat$p02_scr_us), 'recovery_at_1_month'] <- 0

dat[which(dat$renal_function_status7d2 == 2 & dat$p21_m3fu_scr <= dat$p02_scr_us), 'recovery_at_3_month']  <- 1
dat[which(dat$renal_function_status7d2 == 2 & dat$p21_m3fu_scr > dat$p02_scr_us), 'recovery_at_3_month'] <- 0

dat[which(dat$renal_function_status7d2 == 2 & dat$p21_m6fu_scr <= dat$p02_scr_us), 'recovery_at_6_month'] <- 1
dat[which(dat$renal_function_status7d2 == 2 & dat$p21_m6fu_scr > dat$p02_scr_us), 'recovery_at_6_month'] <- 0

dat$renal_function_at_admission.old <- dat$renal_function_at_admission
dat$renal_function_status7d.old <- dat$renal_function_status7d

dat$renal_function_at_admission <- dat$renal_function_at_admission2
dat$renal_function_status7d <- dat$renal_function_status7d2

dat$recovery_at_3_month.o <- dat$recovery_at_3_month

```

```r
dat$recovery_at_1_month <- gsubVec(c(0, 1, 2, 3, 4, 5, 6), c('AKI', 'Recovery from AKI', 'New onset AKD', 'Recovery from AKD', 'NKD', 'AKD', 'CKD'), dat$recovery_at_1_month)
dat$recovery_at_3_month <- gsubVec(c(0, 1, 2, 3, 4, 5, 6, 7),
                                   c('AKI', 'Recovery from AKI', 'New onset AKD', 'Recovery from AKD', 'NKD', 'New onset CKD', 'Stable CKD', 'CKD progression'),
                                   dat$recovery_at_3_month)
dat$recovery_at_6_month <- gsubVec(c(0, 1, 2, 3, 4, 5, 6, 7),
                                   c('AKI', 'Recovery from AKI', 'New onset AKD', 'Recovery from AKD', 'NKD', 'New onset CKD', 'Stable CKD', 'CKD progression'),
                                   dat$recovery_at_6_month)
##########

#dat$Enrol_eGFR_less_60.new <- ifelse(dat$p21_enrol_gfr < 60, 1, 0)
#dat[which(dat$renal_function_at_admission == 2 & dat$proteinuria_at_enrollment == 0), 'renal_function_at_admission_AKD_by_eGFR_only'] <- 1

#dat$renal_function_at_admission_need_change <- ifelse(dat$renal_function_at_admission_AKD_by_eGFR_only == 1 & dat$Enrol_eGFR_less_60 == 0, 1, 0)

#dat[which(dat$renal_function_at_admission_need_change == 1), 'renal_function_at_admission'] <- 1
## Appendix Table 2 - Mortality at discharge
dat[which(is.na(dat$mortality_at_discharge)), c("mortality_at_discharge")] <- 0
dat[which(dat$final_disposition_with_mortality != 1), c("mortality_at_discharge")] <- NA
dat$mortality_at_discharge <- factor(gsubVec(c(0, 1), c('No', 'Yes'), dat$mortality_at_discharge), levels = c('Yes', 'No'))
dat$renal_function_status7d <- factor(gsubVec(c(1, 2, 3, 4), c('NKD', 'AKI', 'AKD', 'CKD'), dat$renal_function_status7d), levels = c('NKD', 'AKI', 'AKD', 'CKD'))
dat$Dialysis_indication_final <- gsubVec(c(0, 1, 2), c('Not Dialyzed', 'Dialysis indication but not dialyzed', 'Dialyzed'), dat$Dialysis_indication_final)

dat$mortality_at_discharge.NKD <- dat$mortality_at_discharge.no.aki <- dat$mortality_at_discharge.aki <- dat$mortality_at_discharge.stg1 <- dat$mortality_at_discharge.stg2 <- dat$mortality_at_discharge.stg3 <- dat$mortality_at_discharge.stg23 <- dat$mortality_at_discharge.indi.nodia <- dat$mortality_at_discharge.dia <- dat$mortality_at_discharge.akd <- dat$mortality_at_discharge.ckd <- dat$mortality_at_discharge

dat[which(dat$renal_function_status7d != 'NKD'), 'mortality_at_discharge.NKD'] <- NA
dat[which(dat$renal_function_status7d == 'AKI'), 'mortality_at_discharge.no.aki'] <- NA
dat[which(dat$renal_function_status7d != 'AKI'), 'mortality_at_discharge.aki'] <- NA
dat[which(dat$renal_function_status7d != 'AKI' | dat$Final_total_AKI_stage != 1), 'mortality_at_discharge.stg1'] <- NA
dat[which(dat$renal_function_status7d != 'AKI' | dat$Final_total_AKI_stage != 2), 'mortality_at_discharge.stg2'] <- NA
dat[which(dat$renal_function_status7d != 'AKI' | dat$Final_total_AKI_stage != 3), 'mortality_at_discharge.stg3'] <- NA
dat[which(dat$renal_function_status7d != 'AKI' | !dat$Final_total_AKI_stage %in% c(2, 3)), 'mortality_at_discharge.stg23'] <- NA
dat[which(dat$Dialysis_indication_final != 'Dialysis indication but not dialyzed'), 'mortality_at_discharge.indi.nodia'] <- NA
dat[which(dat$Dialysis_indication_final != 'Dialyzed'), 'mortality_at_discharge.dia'] <- NA
dat[which(dat$renal_function_status7d != 'AKD'), 'mortality_at_discharge.akd'] <- NA
dat[which(dat$renal_function_status7d != 'CKD'), 'mortality_at_discharge.ckd'] <- NA


app.tab2.1 <- demoTab(data = dat, group = 'phase.cat', vars = c('mortality_at_discharge', 'mortality_at_discharge.NKD', 'mortality_at_discharge.no.aki', 'mortality_at_discharge.aki',
                                              'mortality_at_discharge.stg1', 'mortality_at_discharge.stg2', 'mortality_at_discharge.stg3', 'mortality_at_discharge.stg23',
                                              'mortality_at_discharge.indi.nodia', 'mortality_at_discharge.dia', 'mortality_at_discharge.akd', 'mortality_at_discharge.ckd'),
        cat.tests = 'fisher.test', rnames = c('{\\bf Mortality at discharge - Hospitalized patients}', 'NKD', 'No AKI', 'AKI', '~~~stage 1', '~~~stage 2', '~~~stage 3', '~~~severe AKI (stage2/3)',
                                          'Dialysis indication but not dialyzed', 'Dialyzed', 'AKD', 'CKD'))

rownames(app.tab2.1)[seq(2, 36, 3)] <- rownames(app.tab2.1)[seq(1, 36, 3)]
final <- app.tab2.1[seq(2, 36, 3), ]

size <- app.tab2.1[seq(1, 36, 3), 1:3]

dol.loc <- matrix(rep(NA, 36), nrow = 12, ncol = 3)
for(i in 1:(ncol(final) - 1)){
  for(j in 1:nrow(final)){
    dol.loc[j, i] <- gregexpr(pattern ='\\$', final[j, i])[[1]][2]
  }
}

deno.size <- matrix(rep(NA, 36), nrow = 12, ncol = 3)
for(i in 1:ncol(size)){
  for(j in 1:nrow(size)){
    deno.size[j, i] <- as.numeric(str_extract(size[j, i], "(?<=\\=)[^;]*(?=\\$)"))
  }
}
```

```
for(i in 1:(ncol(final) - 1)){
  for(j in 1:nrow(final)){
    stri_sub(final[j, i], dol.loc[j, i], dol.loc[j, i] - 1) <- paste('/', deno.size[j, i], sep = '')
  }
}

app.tab2.d <- final



dat[which(is.na(dat$Mortality_overall)), c("patient_id", "mortality_at_discharge", 'mortality_follow_up', "Mortality_overall")]

dat[which(dat$mortality_at_discharge == 'Yes'), c("patient_id", "mortality_at_discharge", 'mortality_follow_up')]
#pc <- "bt43xx"
#paste(append(strsplit(pc, "")[[1]], "+", nchar(pc) - 3), collapse = "")

final %<>% lapply (function(x) gregexpr(pattern ='\\$', x)[[1]][2])

#gregexpr(pattern ='\\$', final[1,1])[[1]][2]

## Appendix Table 2 - Mortality during follow up - Hospitalized
dat$mortality_follow_up.o <- dat$mortality_follow_up
dat$mortality_follow_up <- factor(gsubVec(c(0, 1), c('No', 'Yes'), dat$mortality_follow_up.o), levels = c('Yes', 'No'))
dat[which(dat$mortality_at_discharge == 'Yes'), "mortality_follow_up"] <- NA
dat[which(dat$final_disposition_with_mortality != 1), c("mortality_follow_up")] <- NA

dat$renal_function_status7d <- gsubVec(c(1, 2, 3, 4), c('NKD', 'AKI', 'AKD', 'CKD'), dat$renal_function_status7d)
dat$Dialysis_indication_final <- gsubVec(c(0, 1, 2), c('Not Dialyzed', 'Dialysis indication but not dialyzed', 'Dialyzed'), dat$Dialysis_indication_final)

dat$mortality_follow_up.NKD <- dat$mortality_follow_up.no.aki <- dat$mortality_follow_up.aki <- dat$mortality_follow_up.stg1 <- dat$mortality_follow_up.stg2 <- dat$mortality_follow_up.stg3 <- dat$mortality_follow_up.stg23 <- dat$mortality_follow_up.indi.nodia <- dat$mortality_follow_up.dia <- dat$mortality_follow_up.akd <- dat$mortality_follow_up.ckd <- dat$mortality_follow_up

dat[which(dat$renal_function_status7d != 'NKD'), 'mortality_follow_up.NKD'] <- NA
dat[which(dat$renal_function_status7d == 'AKI'), 'mortality_follow_up.no.aki'] <- NA
dat[which(dat$renal_function_status7d != 'AKI'), 'mortality_follow_up.aki'] <- NA
dat[which(dat$renal_function_status7d != 'AKI' | dat$Final_total_AKI_stage != 1), 'mortality_follow_up.stg1'] <- NA
dat[which(dat$renal_function_status7d != 'AKI' | dat$Final_total_AKI_stage != 2), 'mortality_follow_up.stg2'] <- NA
dat[which(dat$renal_function_status7d != 'AKI' | dat$Final_total_AKI_stage != 3), 'mortality_follow_up.stg3'] <- NA
dat[which(dat$renal_function_status7d != 'AKI' | !dat$Final_total_AKI_stage %in% c(2, 3)), 'mortality_follow_up.stg23'] <- NA
dat[which(dat$Dialysis_indication_final != 'Dialysis indication but not dialyzed'), 'mortality_follow_up.indi.nodia'] <- NA
dat[which(dat$Dialysis_indication_final != 'Dialyzed'), 'mortality_follow_up.dia'] <- NA
dat[which(dat$renal_function_status7d != 'AKD'), 'mortality_follow_up.akd'] <- NA
dat[which(dat$renal_function_status7d != 'CKD'), 'mortality_follow_up.ckd'] <- NA


app.tab2.2 <- demoTab(data = dat, group = 'phase.cat', vars = c('mortality_follow_up', 'mortality_follow_up.NKD', 'mortality_follow_up.no.aki', 'mortality_follow_up.aki',
                                                   'mortality_follow_up.stg1', 'mortality_follow_up.stg2', 'mortality_follow_up.stg3', 'mortality_follow_up.stg23',
                                                   'mortality_follow_up.indi.nodia', 'mortality_follow_up.dia', 'mortality_follow_up.akd', 'mortality_follow_up.ckd'),
        cat.tests = 'fisher.test', rnames = c('{\\bf Mortality during follow up - Hospitalized patients}', 'NKD', 'No AKI', 'AKI', '~~~stage 1', '~~~stage 2', '~~~stage 3', '~~~severe AKI (stage2/3)',
                                               'Dialysis indication but not dialyzed', 'Dialyzed', 'AKD', 'CKD'))


rownames(app.tab2.2)[seq(2, 36, 3)] <- rownames(app.tab2.2)[seq(1, 36, 3)]
final <- app.tab2.2[seq(2, 36, 3), ]

size <- app.tab2.2[seq(1, 36, 3), 1:3]

dol.loc <- matrix(rep(NA, 36), nrow = 12, ncol = 3)
for(i in 1:(ncol(final) - 1)){
  for(j in 1:nrow(final)){
    dol.loc[j, i] <- gregexpr(pattern ='\\$', final[j, i])[[1]][2]
  }
}

deno.size <- matrix(rep(NA, 36), nrow = 12, ncol = 3)
for(i in 1:ncol(size)){
  for(j in 1:nrow(size)){
    deno.size[j, i] <- as.numeric(str_extract(size[j, i], "(?<=\\=)[^;]*(?=\\$)"))
```

```r
  }
}

for(i in 1:(ncol(final) - 1)){
  for(j in 1:nrow(final)){
    stri_sub(final[j, i], dol.loc[j, i], dol.loc[j, i] - 1) <- paste('/', deno.size[j, i], sep = '')
  }
}

app.tab2.f <- final


## Appendix Table 2 - Mortality during follow up - Sent Home
dat$mortality_follow_up <- factor(gsubVec(c(0, 1), c('No', 'Yes'), dat$mortality_follow_up.o), levels = c('Yes', 'No'))
dat[which(dat$mortality_at_discharge == 'Yes'), "mortality_follow_up"] <- NA
dat[which(dat$final_disposition_with_mortality != 2), c("mortality_follow_up")] <- NA

dat$renal_function_status7d <- gsubVec(c(1, 2, 3, 4), c('NKD', 'AKI', 'AKD', 'CKD'), dat$renal_function_status7d)
dat$Dialysis_indication_final <- gsubVec(c(0, 1, 2), c('Not Dialyzed', 'Dialysis indication but not dialyzed', 'Dialyzed'), dat$Dialysis_indication_final)

dat$mortality_follow_up.NKD <- dat$mortality_follow_up.no.aki <- dat$mortality_follow_up.aki <- dat$mortality_follow_up.stg1 <- dat$mortality_follow_up.stg2 <- dat$mortality_follow_up.stg3 <- dat$mortality_follow_up.stg23 <- dat$mortality_follow_up.indi.nodia <- dat$mortality_follow_up.dia <- dat$mortality_follow_up.akd <- dat$mortality_follow_up.ckd <- dat$mortality_follow_up

dat[which(dat$renal_function_status7d != 'NKD'), 'mortality_follow_up.NKD'] <- NA
dat[which(dat$renal_function_status7d == 'AKI'), 'mortality_follow_up.no.aki'] <- NA
dat[which(dat$renal_function_status7d != 'AKI'), 'mortality_follow_up.aki'] <- NA
dat[which(dat$renal_function_status7d != 'AKI' | dat$Final_total_AKI_stage != 1), 'mortality_follow_up.stg1'] <- NA
dat[which(dat$renal_function_status7d != 'AKI' | dat$Final_total_AKI_stage != 2), 'mortality_follow_up.stg2'] <- NA
dat[which(dat$renal_function_status7d != 'AKI' | dat$Final_total_AKI_stage != 3), 'mortality_follow_up.stg3'] <- NA
dat[which(dat$renal_function_status7d != 'AKI' | !dat$Final_total_AKI_stage %in% c(2, 3)), 'mortality_follow_up.stg23'] <- NA
dat[which(dat$Dialysis_indication_final != 'Dialysis indication but not dialyzed'), 'mortality_follow_up.indi.nodia'] <- NA
dat[which(dat$Dialysis_indication_final != 'Dialyzed'), 'mortality_follow_up.dia'] <- NA
dat[which(dat$renal_function_status7d != 'AKD'), 'mortality_follow_up.akd'] <- NA
dat[which(dat$renal_function_status7d != 'CKD'), 'mortality_follow_up.ckd'] <- NA


app.tab2.2 <- demoTab(data = dat, group = 'phase.cat', vars = c('mortality_follow_up', 'mortality_follow_up.NKD', 'mortality_follow_up.no.aki', 'mortality_follow_up.aki',
                                                  'mortality_follow_up.stg1', 'mortality_follow_up.stg2', 'mortality_follow_up.stg3', 'mortality_follow_up.stg23',
                                                  'mortality_follow_up.indi.nodia', 'mortality_follow_up.dia', 'mortality_follow_up.akd', 'mortality_follow_up.ckd'),
        cat.tests = 'fisher.test', rnames = c('{\\bf Mortality during follow up - Non-hospitalized patients}', 'NKD', 'No AKI', 'AKI', '~~~stage 1', '~~~stage 2', '~~~stage 3', '~~~severe AKI (stage2/3)',
                                              'Dialysis indication but not dialyzed', 'Dialyzed', 'AKD', 'CKD'))


rownames(app.tab2.2)[seq(2, 36, 3)] <- rownames(app.tab2.2)[seq(1, 36, 3)]
final <- app.tab2.2[seq(2, 36, 3), ]

size <- app.tab2.2[seq(1, 36, 3), 1:3]

dol.loc <- matrix(rep(NA, 36), nrow = 12, ncol = 3)
for(i in 1:(ncol(final) - 1)){
  for(j in 1:nrow(final)){
    dol.loc[j, i] <- gregexpr(pattern ='\\$', final[j, i])[[1]][2]
  }
}

deno.size <- matrix(rep(NA, 36), nrow = 12, ncol = 3)
for(i in 1:ncol(size)){
  for(j in 1:nrow(size)){
    deno.size[j, i] <- as.numeric(str_extract(size[j, i], "(?<=\\=)[^;]*(?=\\$)"))
  }
}

for(i in 1:(ncol(final) - 1)){
  for(j in 1:nrow(final)){
    stri_sub(final[j, i], dol.loc[j, i], dol.loc[j, i] - 1) <- paste('/', deno.size[j, i], sep = '')
  }
}

app.tab2.f.1 <- final
```

```
## Appendix Table 2 — Overall Mortality
dat[which(is.na(dat$Mortality_overall)), c("Mortality_overall")] <- 0
dat$Mortality_overall <- factor(gsubVec(c(0, 1), c('No', 'Yes'), dat$Mortality_overall), levels = c('Yes', 'No'))
dat$renal_function_status7d <- gsubVec(c(1, 2, 3, 4), c('NKD', 'AKI', 'AKD', 'CKD'), dat$renal_function_status7d)
dat$Dialysis_indication_final <- gsubVec(c(0, 1, 2), c('Not Dialyzed', 'Dialysis indication but not dialyzed', '
    Dialyzed'), dat$Dialysis_indication_final)

dat$Mortality_overall.NKD <- dat$Mortality_overall.no.aki <- dat$Mortality_overall.aki <- dat$Mortality_overall.stg1
    <- dat$Mortality_overall.stg2 <- dat$Mortality_overall.stg3 <- dat$Mortality_overall.stg23 <-
    dat$Mortality_overall.indi.nodia <- dat$Mortality_overall.dia <- dat$Mortality_overall.akd <-
    dat$Mortality_overall.ckd <- dat$Mortality_overall

dat[which(dat$renal_function_status7d != 'NKD'), 'Mortality_overall.NKD'] <- NA
dat[which(dat$renal_function_status7d == 'AKI'), 'Mortality_overall.no.aki'] <- NA
dat[which(dat$renal_function_status7d != 'AKI'), 'Mortality_overall.aki'] <- NA
dat[which(dat$renal_function_status7d != 'AKI' | dat$Final_total_AKI_stage != 1), 'Mortality_overall.stg1'] <- NA
dat[which(dat$renal_function_status7d != 'AKI' | dat$Final_total_AKI_stage != 2), 'Mortality_overall.stg2'] <- NA
dat[which(dat$renal_function_status7d != 'AKI' | dat$Final_total_AKI_stage != 3), 'Mortality_overall.stg3'] <- NA
dat[which(dat$renal_function_status7d != 'AKI' | !dat$Final_total_AKI_stage %in% c(2, 3)), 'Mortality_overall.stg23']
    <- NA
dat[which(dat$Dialysis_indication_final != 'Dialysis indication but not dialyzed'), 'Mortality_overall.indi.nodia'] <-
     NA
dat[which(dat$Dialysis_indication_final != 'Dialyzed'), 'Mortality_overall.dia'] <- NA
dat[which(dat$renal_function_status7d != 'AKD'), 'Mortality_overall.akd'] <- NA
dat[which(dat$renal_function_status7d != 'CKD'), 'Mortality_overall.ckd'] <- NA


app.tab2.3 <- demoTab(data = dat, group = 'phase.cat', vars = c('Mortality_overall', 'Mortality_overall.NKD', '
    Mortality_overall.no.aki', 'Mortality_overall.aki',
                                              'Mortality_overall.stg1', 'Mortality_overall.stg2', '
                                                  Mortality_overall.stg3', 'Mortality_overall.stg23',
                                              'Mortality_overall.indi.nodia', 'Mortality_overall.dia', '
                                                  Mortality_overall.akd', 'Mortality_overall.ckd'),
        cat.tests = 'fisher.test', rnames = c('{\\bf Overall Mortality}', 'NKD', 'No AKI', 'AKI', '~~~stage 1', '~~~
            stage 2', '~~~stage 3', '~~~severe AKI (stage2/3)',
                                          'Dialysis indication but not dialyzed', 'Dialyzed', 'AKD', 'CKD'))


rownames(app.tab2.3)[seq(2, 36, 3)] <- rownames(app.tab2.3)[seq(1, 36, 3)]
final <- app.tab2.3[seq(2, 36, 3), ]

size <- app.tab2.3[seq(1, 36, 3), 1:3]

dol.loc <- matrix(rep(NA, 36), nrow = 12, ncol = 3)
for(i in 1:(ncol(final) - 1)){
  for(j in 1:nrow(final)){
    dol.loc[j, i] <- gregexpr(pattern ='\\$', final[j, i])[[1]][2]
  }
}

deno.size <- matrix(rep(NA, 36), nrow = 12, ncol = 3)
for(i in 1:ncol(size)){
  for(j in 1:nrow(size)){
    deno.size[j, i] <- as.numeric(str_extract(size[j, i], "(?<=\\=)[^;]*(?=\\$)"))
  }
}

for(i in 1:(ncol(final) - 1)){
  for(j in 1:nrow(final)){
    stri_sub(final[j, i], dol.loc[j, i], dol.loc[j, i] - 1) <- paste('/', deno.size[j, i], sep = '')
  }
}

app.tab2.o <- final

app.tab2 <- rbind(app.tab2.d, app.tab2.f, app.tab2.f.1, app.tab2.o)
app.tab2 <- app.tab2[, c(3, 1, 2, 4)]
colnames(app.tab2)[1:3] <- c("\\begin{tabular}{c}Overall\\\\ ($n = 2101$) \\end{tabular}", "\\begin{tabular}{c}
    Observation\\\\ ($n = 978$) \\end{tabular}",
                             "\\begin{tabular}{c}Intervention\\\\ ($n = 1123$) \\end{tabular}")

### Table 2


dat$p01_age.child <- dat$p01_age.adult <- dat$p01_age
dat$p01_is_child <- factor(gsubVec(c(0, 1), c('Yes', 'No'), dat$p01_is_adult), levels = c('Yes', 'No'))
dat$p01_is_adult <- factor(gsubVec(c(0, 1), c('No', 'Yes'), dat$p01_is_adult), levels = c('Yes', 'No'))
```

```r
dat[which(dat$p01_is_adult == 'No'), 'p01_age.adult'] <- NA
dat[which(dat$p01_is_adult == 'Yes'), 'p01_age.child'] <- NA

tab2 <- demoTab(vars = c('p01_is_child', 'p01_age.child', 'p01_is_adult', 'p01_age.adult'), group = 'phase.cat', data = dat, cat.tests = 'fisher.test')
tab2 <- tab2[c(2, 5, 7, 10), ]
rownames(tab2) <- c('Children (<18)', '~~~ Mean Age', 'Adult', '~~~ Mean Age')
tab2 <- tab2[, c(3, 1, 2, 4)]
colnames(tab2)[1:3] <- c("\\begin{tabular}{c}Overall\\\\ ($n = 2101$) \\end{tabular}", "\\begin{tabular}{c}Observation\\\\ ($n = 978$) \\end{tabular}",
                         "\\begin{tabular}{c}Intervention\\\\ ($n = 1123$) \\end{tabular}")
## 4 patients with unkown age/age group
dat[which(is.na(dat$p01_is_adult)), c('patient_id', 'p01_is_adult', 'p01_age')]


## Table 4

table(dat$eGFR_less_than75_at_enrol)
dat$Enrol_eGFR_less_60.new <- ifelse(dat$p21_enrol_gfr < 60, 2, 0)

table(dat$proteinuria_at_enrollment)
#dat$eGFR_less_than75_at_enrol.new <- as.numeric(gsub(1, 2, dat$Enrol_eGFR_less_60.new))
dat[which(is.na(dat$Enrol_eGFR_less_60.new)), 'Enrol_eGFR_less_60.new'] <- 0
dat$akd_cause_enrol <- dat$Enrol_eGFR_less_60.new + dat$proteinuria_at_enrollment
dat$akd_cause_enrol <- factor(gsubVec(c(0, 1, 2, 3), c(NA, 'Based on albuminuria alone', 'Based on sCr alone', 'Based on sCr and albuminuria'), dat$akd_cause_enrol),
                              levels = c('Based on sCr alone', 'Based on sCr and albuminuria', 'Based on albuminuria alone'))

adm.overall <- fisher.test(dat$renal_function_at_admission, dat$phase.cat)

dat$ckd_adm <- dat$akd_adm <- dat$NKD_adm <- dat$renal_function_at_admission

dat[which(dat$renal_function_at_admission != 1), 'NKD_adm'] <- 0
dat[which(dat$renal_function_at_admission != 2), 'akd_adm'] <- 1
dat[which(dat$renal_function_at_admission != 3), 'ckd_adm'] <- 1
dat[which(dat$renal_function_at_admission != 2), 'akd_cause_enrol'] <- NA

dat$ckd_adm_alb <- dat$ckd_adm
dat[which(dat$renal_function_at_admission != 3), 'ckd_adm_alb'] <- NA
dat[which(dat$proteinuria_at_enrollment != 1), 'ckd_adm_alb'] <- 1

dat$NKD_adm <- factor(gsubVec(c(0, 1), c('No', 'Yes'), dat$NKD_adm), levels = c('Yes', 'No'))
dat$akd_adm <- factor(gsubVec(c(1, 2), c('No', 'Yes'), dat$akd_adm), levels = c('Yes', 'No'))
dat$ckd_adm <- factor(gsubVec(c(1, 3), c('No', 'Yes'), dat$ckd_adm), levels = c('Yes', 'No'))
dat$ckd_adm_alb <- factor(gsubVec(c(1, 3), c('No', 'Yes'), dat$ckd_adm_alb), levels = c('Yes', 'No'))


tab4.1 <- demoTab(vars = c('ckd_adm', 'ckd_adm_alb', 'akd_adm', 'akd_cause_enrol', 'NKD_adm'), group = 'phase.cat', data = dat,
                  rnames= c('CKD', '~~~ With albuminuria', 'AKD', 'AKD_base', 'NKD'), cat.tests = 'fisher.test')
rownames(tab4.1)[c(2, 5, 8, 15)] <- rownames(tab4.1)[c(1, 4, 7, 14)]
tab4.1 <- tab4.1[c(2, 5, 8, 11, 12, 13, 15), ]
tab4.1[c(1, 2, 3, 7), 4] <- ''
tab4.1[4, 4] <- pvalFormat(adm.overall$p.value)

dat$ckd_7d <- dat$akd_7d <- dat$NKD_7d <- dat$aki_7d <- dat$renal_function_status7d

dat[which(dat$renal_function_status7d != 'NKD'), 'NKD_7d'] <- 'No'
dat[which(dat$renal_function_at_admission != 1), 'NKD_7d'] <- NA
dat[which(dat$renal_function_status7d != 'AKD'), 'akd_7d'] <- 'No'
dat[which(dat$renal_function_at_admission != 2), 'akd_7d'] <- NA
dat[which(dat$renal_function_status7d != 'CKD'), 'ckd_7d'] <- 'No'
dat[which(dat$renal_function_at_admission != 3), 'ckd_7d'] <- NA
dat[which(dat$renal_function_status7d != 'AKI'), 'aki_7d'] <- 'No'

dat$NKD_7d <- factor(gsubVec(c('NKD'), c('Yes'), dat$NKD_7d), levels = c('Yes', 'No'))
dat$akd_7d <- factor(gsubVec(c('AKD'), c('Yes'), dat$akd_7d), levels = c('Yes', 'No'))
dat$ckd_7d <- factor(gsubVec(c('CKD'), c('Yes'), dat$ckd_7d), levels = c('Yes', 'No'))
dat$aki_7d <- factor(gsubVec(c('AKI'), c('Yes'), dat$aki_7d), levels = c('Yes', 'No'))
dat$noaki_7d <- factor(dat$aki_7d, levels = c('No', 'Yes'))

dat$aki_7d.mod <- dat$aki_7d
dat[which(dat$aki_7d == 'No'), 'aki_7d.mod'] <- NA
dat$aki_7d_stg1 <- dat$aki_7d_stg2 <- dat$aki_7d_stg3 <- dat$aki_7d_stg23 <- dat$aki_7d.mod
dat[which(dat$renal_function_status7d == 'AKI' & dat$Final_total_AKI_stage != 1), 'aki_7d_stg1'] <- 'No'
dat[which(dat$renal_function_status7d == 'AKI' & dat$Final_total_AKI_stage != 2), 'aki_7d_stg2'] <- 'No'
dat[which(dat$renal_function_status7d == 'AKI' & dat$Final_total_AKI_stage != 3), 'aki_7d_stg3'] <- 'No'
dat[which(dat$renal_function_status7d == 'AKI' & !dat$Final_total_AKI_stage %in% c(2, 3)), 'aki_7d_stg23'] <- 'No'
```

```
tab4.2 <- demoTab(vars = c('noaki_7d', 'ckd_7d', 'akd_7d', 'NKD_7d', 'aki_7d', 'aki_7d_stg1', 'aki_7d_stg2', 'aki_7d_stg3', 'aki_7d_stg23'), group = 'phase.cat', data = dat,
                  rnames = c('No AKI', 'CKD', 'AKD', 'NKD', 'AKI', '~~~ Stage 1', '~~~ Stage 2', '~~~ Stage 3', '~~~ severe AKI (stage2/3)'), cat.tests = 'fisher.test')

rownames(tab4.2)[seq(2, 27, 3)] <- rownames(tab4.2)[seq(1, 27, 3)]
tab4.2 <- tab4.2[seq(2, 27, 3), ]


dat$final_disposition_cat <- factor(gsubVec(c(1, 2), c('Admitted', 'Sent home'), dat$final_disposition_with_mortality), levels = c('Sent home', 'Admitted'))

dat$ckd_dis <- dat$akd_dis <- dat$NKD_dis <- dat$aki_dis <- dat$renal_function_status7d

dat[which(dat$renal_function_status7d != 'NKD'), 'NKD_dis'] <- 'No'
dat[which(dat$renal_function_status7d != 'AKD'), 'akd_dis'] <- 'No'
dat[which(dat$renal_function_status7d != 'CKD'), 'ckd_dis'] <- 'No'
dat[which(dat$renal_function_status7d != 'AKI'), 'aki_dis'] <- 'No'

dat$NKD_dis <- factor(gsubVec(c('NKD'), c('Yes'), dat$NKD_dis), levels = c('Yes', 'No'))
dat$akd_dis <- factor(gsubVec(c('AKD'), c('Yes'), dat$akd_dis), levels = c('Yes', 'No'))
dat$ckd_dis <- factor(gsubVec(c('CKD'), c('Yes'), dat$ckd_dis), levels = c('Yes', 'No'))
dat$aki_dis <- factor(gsubVec(c('AKI'), c('Yes'), dat$aki_dis), levels = c('Yes', 'No'))

dat$Time_between_screening_and_discharge.new <- dat$Time_between_screening_and_discharge
dat[which(dat$Time_between_screening_and_discharge < 0), 'Time_between_screening_and_discharge.new'] <- NA

###################
dat$p01_rec_dtime <- as.numeric(dat$p01_rec_dtime)
dat[["p01_rec_dtime"]] <-
  as.POSIXct(dat[["p01_rec_dtime"]] * (60*60*24)
    , origin="1899-12-30"
    , tz="UTC")

dat$p05_rec_dtime <- as.numeric(dat$p05_rec_dtime)
dat[["p05_rec_dtime"]] <-
  as.POSIXct(dat[["p05_rec_dtime"]] * (60*60*24)
    , origin="1899-12-30"
    , tz="UTC")

dat$p05_rec_dtime - dat$p01_rec_dtime
dat$Time_between_screening_and_discharge.new <- as.numeric(difftime(dat$p05_rec_dtime, dat$p01_rec_dtime, units="hours"))
#######################
dat[which(dat$Time_between_screening_and_discharge.new < 0), 'Time_between_screening_and_discharge.new'] <- NA
dat$Time_between_screening_and_discharge.new.home <- dat$Time_between_screening_and_discharge.new.adm <- dat$Time_between_screening_and_discharge.new

dat[which(dat$final_disposition_cat != 'Admitted'), 'Time_between_screening_and_discharge.new.adm'] <- NA
dat[which(dat$final_disposition_cat != 'Sent home'), 'Time_between_screening_and_discharge.new.home'] <- NA

dat[which(dat$final_disposition_cat != 'Admitted'), c('ckd_dis', 'aki_dis', 'akd_dis', 'NKD_dis')] <- NA


time.all <- wilcox.test(dat[which(dat$phase.cat == 'Observation'), 'Time_between_screening_and_discharge.new'], dat[which(dat$phase.cat == 'Intervention'), 'Time_between_screening_and_discharge.new'])

kruskal.test(dat$Time_between_screening_and_discharge.new, dat$phase.cat)

time.home <- wilcox.test(dat[which(dat$phase.cat == 'Observation'), 'Time_between_screening_and_discharge.new.home'], dat[which(dat$phase.cat == 'Intervention'), 'Time_between_screening_and_discharge.new.home'])

time.adm <- wilcox.test(dat[which(dat$phase.cat == 'Observation'), 'Time_between_screening_and_discharge.new.adm'], dat[which(dat$phase.cat == 'Intervention'), 'Time_between_screening_and_discharge.new.adm'])


tab4.3 <- demoTab(vars = c('final_disposition_cat', 'NKD_dis', 'aki_dis', 'akd_dis', 'ckd_dis', 'Time_between_screening_and_discharge.new', 'Time_between_screening_and_discharge.new.home', 'Time_between_screening_and_discharge.new.adm'), group = 'phase.cat', data = dat,
                  rnames= c('Disposition', '~~~ NKD', '~~~ AKI','~~~ AKD','~~~ CKD', 'Time(hours) in health care facility in all patients', '~~~ Sent home', '~~~ Admitted'),
                  cent.funs = rep(c('median'), 100), var.funs = rep(c('quantile'), 100), cat.tests = 'fisher.test')
rownames(tab4.3)[c(seq(5, 15, 3), 17, 19, 21)] <- rownames(tab4.3)[c(seq(4, 15, 3), 16, 18, 20)]
tab4.3 <- tab4.3[c(1:3, c(seq(5, 15, 3), 17, 19, 21)), ]
rownames(tab4.3)[c(2, 3)] <- gsub('~~~~', '', rownames(tab4.3)[c(2, 3)])
tab4.3[1, ] <- ''
```

```r
tab4.3[8, 4] <- pvalFormat(time.all$p.value)
tab4.3[9, 4] <- pvalFormat(time.home$p.value)
tab4.3[10, 4] <- pvalFormat(time.adm$p.value)

dat$dialysis_indi <- dat$dialysis_dialyze <- dat$Dialysis_indication_final
dat$indi_7d <- dat$dialy_7d <- dat$renal_function_status7d

dat[which(dat$Dialysis_indication_final != 'Dialysis indication but not dialyzed'), 'dialysis_indi'] <- 0
dat[which(dat$Dialysis_indication_final != 'Dialyzed'), 'dialysis_dialyze'] <- 0
dat[which(dat$Dialysis_indication_final != 'Dialysis indication but not dialyzed'), 'indi_7d'] <- NA
dat[which(dat$Dialysis_indication_final != 'Dialyzed'), 'dialy_7d'] <- NA

dat$dialysis_was_indicateyn <- factor(gsubVec(c(0, 1), c('No', 'Yes'), dat$dialysis_was_indicateyn), levels = c('Yes', 'No'))
dat$dialysis_dialyze <- factor(gsubVec(c(0, 'Dialyzed'), c('No', 'Yes'), dat$dialysis_dialyze), levels = c('Yes', 'No'))
dat$dialysis_indi <- factor(gsubVec(c(0, 'Dialysis indication but not dialyzed'), c('No', 'Yes'), dat$dialysis_indi), levels = c('Yes', 'No'))

tab4.4 <- demoTab(vars = c('dialysis_was_indicateyn', 'dialysis_dialyze', 'dialy_7d', 'dialysis_indi','indi_7d'), group = 'phase.cat', data = dat, show.na = FALSE,
                  rnames = c('Dialysis Indiction', 'Dialyzed', 'dia_status', 'Dialysis indication, but not dialyzed', 'indi_status'), cat.tests = 'fisher.test')
rownames(tab4.4)[c(2, 5, 12)] <- rownames(tab4.4)[c(1, 4, 11)]
tab4.4 <- tab4.4[c(2, 5, 8, 9, 10, 12, 15, 16, 17, 18), ]

tab4 <- rbind(tab4.1, tab4.2, tab4.3, tab4.4)
tab4 <- tab4[, c(3, 1, 2, 4)]
colnames(tab4)[1:3] <- c("\\begin{tabular}{c}Overall\\\\ ($n = 2101$) \\end{tabular}", "\\begin{tabular}{c}Observation\\\\ ($n = 978$) \\end{tabular}",
                         "\\begin{tabular}{c}Intervention\\\\ ($n = 1123$) \\end{tabular}")



## Table 6
# dat$CKD_cat_3month_prog <- dat$CKD_cat_enrollment_gfr - dat$CKD_cat_m3fu_gfr
# dat[which(dat$CKD_cat_3month_prog > 0), 'CKD_cat_3month_prog'] <- 0
# dat[which(dat$CKD_cat_3month_prog < 0), 'CKD_cat_3month_prog'] <- 1
dat[, 'CKD_cat_3month_prog'] <- NA
dat[which(dat$recovery_at_3_month == 'CKD progression' & dat$sCr3month == 1), 'CKD_cat_3month_prog'] <- 'Yes'
dat[which(dat$recovery_at_3_month == 'Stable CKD' & dat$sCr3month == 1), 'CKD_cat_3month_prog'] <- 'No'
dat$CKD_cat_3month_prog <-  factor(dat$CKD_cat_3month_prog, levels = c('Yes', 'No'))

#
# dat$CKD_cat_6month_prog <- dat$CKD_cat_enrollment_gfr - dat$CKD_cat_m6fu_gfr
# dat[which(dat$CKD_cat_6month_prog > 0), 'CKD_cat_6month_prog'] <- 0
# dat[which(dat$CKD_cat_6month_prog < 0), 'CKD_cat_6month_prog'] <- 1
# dat[which(dat$renal_function_status7d != 'CKD'), 'CKD_cat_6month_prog'] <- NA
# dat$CKD_cat_6month_prog <-  factor(gsubVec(c(0, 1), c('No', 'Yes'), dat$CKD_cat_6month_prog), levels = c('Yes', 'No'))
#
dat[, 'CKD_cat_6month_prog'] <- NA
dat[which(dat$recovery_at_6_month == 'CKD progression' & dat$sCr6month == 1), 'CKD_cat_6month_prog'] <- 'Yes'
dat[which(dat$recovery_at_6_month == 'Stable CKD' & dat$sCr6month == 1), 'CKD_cat_6month_prog'] <- 'No'
dat$CKD_cat_6month_prog <-  factor(dat$CKD_cat_6month_prog, levels = c('Yes', 'No'))


# dat$Recovery_at_3months_bin <- gsub(1, 0, dat$NRecovery_at_3month)
# dat$Recovery_at_6months_bin <- gsub(1, 0, dat$NRecovery_at_6month)
# dat$Recovery_at_3months_bin <- factor(gsubVec(c(0, 2), c('No', 'Yes'), dat$Recovery_at_3months_bin), levels = c('Yes', 'No'))
# dat$Recovery_at_6months_bin <- factor(gsubVec(c(0, 2), c('No', 'Yes'), dat$Recovery_at_6months_bin), levels = c('Yes', 'No'))

dat[which(!is.na(dat$recovery_at_3_month) & dat$sCr3month == 1), 'Recovery_at_3months_bin'] <- 'No'
dat[which(dat$recovery_at_3_month %in% c('NKD', 'Recovery from AKD', 'Recovery from AKI') & dat$sCr3month == 1), 'Recovery_at_3months_bin'] <- 'Yes'

dat[which(!is.na(dat$recovery_at_6_month) & dat$sCr6month == 1), 'Recovery_at_6months_bin'] <- 'No'
dat[which(dat$recovery_at_6_month %in% c('NKD', 'Recovery from AKD', 'Recovery from AKI') & dat$sCr6month == 1), 'Recovery_at_6months_bin'] <- 'Yes'

dat$Recovery_at_3months_bin <- factor(dat$Recovery_at_3months_bin, levels = c('Yes', 'No'))
dat$Recovery_at_6months_bin <- factor(dat$Recovery_at_6months_bin, levels = c('Yes', 'No'))

dat$eGFR3months_60 <- ifelse(dat$p21_m3fu_gfr >= 60, 1, 0)
dat$eGFR6months_60 <- ifelse(dat$p21_m6fu_gfr >= 60, 1, 0)

#table(dat$eGFR3months_60, dat$recovery_at_3_month)
```

```r

dat$onset.NKD.3mon <- NA
dat[which(dat$renal_function_status7d == 'NKD' & dat$recovery_at_3_month == 'NKD' & dat$sCr3month == 1), 'onset.NKD.3mon'] <- 'No'
dat[which(dat$renal_function_status7d == 'NKD' & dat$recovery_at_3_month == 'New onset AKD' & dat$sCr3month == 1), 'onset.NKD.3mon'] <- 'Yes'

dat$onset.aki.3mon <- NA
dat[which(dat$renal_function_status7d == 'AKI' & dat$recovery_at_3_month %in% c('Recovery from AKI') & dat$sCr3month == 1), 'onset.aki.3mon'] <- 'No'
dat[which(dat$renal_function_status7d == 'AKI' & dat$recovery_at_3_month == 'AKI' & dat$sCr3month == 1), 'onset.aki.3mon'] <- 'Yes'

dat$onset.akd.3mon <- NA
dat[which(dat$renal_function_status7d == 'AKD' & dat$recovery_at_3_month == 'Recovery from AKD' & dat$sCr3month == 1), 'onset.akd.3mon'] <- 'No'
dat[which(dat$renal_function_status7d == 'AKD' & dat$recovery_at_3_month == 'New onset CKD' & dat$sCr3month == 1), 'onset.akd.3mon'] <- 'Yes'

dat$onset.NKD.6mon <- NA
dat[which(dat$renal_function_status7d == 'NKD' & dat$recovery_at_6_month == 'NKD' & dat$sCr6month == 1), 'onset.NKD.6mon'] <- 'No'
dat[which(dat$renal_function_status7d == 'NKD' & dat$recovery_at_6_month == 'New onset AKD' & dat$sCr6month == 1), 'onset.NKD.6mon'] <- 'Yes'

dat$onset.aki.6mon <- NA
dat[which(dat$renal_function_status7d == 'AKI' & dat$recovery_at_6_month == 'Recovery from AKI' & dat$sCr6month == 1), 'onset.aki.6mon'] <- 'No'
dat[which(dat$renal_function_status7d == 'AKI' & dat$recovery_at_6_month == 'AKI'  & dat$sCr6month == 1), 'onset.aki.6mon'] <- 'Yes'

dat$onset.akd.6mon <- NA
dat[which(dat$renal_function_status7d == 'AKD' & dat$recovery_at_6_month == 'Recovery from AKD' & dat$sCr6month == 1), 'onset.akd.6mon'] <- 'No'
dat[which(dat$renal_function_status7d == 'AKD' & dat$recovery_at_6_month == 'New onset CKD' & dat$sCr6month == 1), 'onset.akd.6mon'] <- 'Yes'

dat$denovo.ckd.3mon <- NA
dat[which(dat$renal_function_status7d %in% c('NKD', 'AKI', 'AKD') & !is.na(dat$recovery_at_3_month) & dat$sCr3month == 1), 'denovo.ckd.3mon'] <-'No'
dat[which(dat$renal_function_status7d %in% c('NKD', 'AKI', 'AKD') & dat$recovery_at_3_month == 'New onset CKD' & dat$sCr3month == 1), 'denovo.ckd.3mon'] <- 'Yes'
dat[which(dat$renal_function_status7d %in% c('NKD', 'AKI', 'AKD') & dat$onset.aki.3mon == 'Yes' & dat$sCr3month == 1), 'denovo.ckd.3mon'] <- 'Yes'

dat$denovo.ckd.6mon <- NA
dat[which(dat$renal_function_status7d %in% c('NKD', 'AKI', 'AKD') & !is.na(dat$recovery_at_6_month) & dat$sCr6month == 1), 'denovo.ckd.6mon'] <-'No'
dat[which(dat$renal_function_status7d %in% c('NKD', 'AKI', 'AKD') & dat$recovery_at_6_month == 'New onset CKD' & dat$sCr6month == 1), 'denovo.ckd.6mon'] <- 'Yes'
dat[which(dat$renal_function_status7d %in% c('NKD', 'AKI', 'AKD') & dat$onset.aki.6mon == 'Yes' & dat$sCr6month == 1), 'denovo.ckd.6mon'] <- 'Yes'

dat$denovo.ckd_overall <- dat$denovo.ckd.3mon
dat[which(dat$denovo.ckd.6mon == 'Yes'), 'denovo.ckd_overall'] <- 'Yes'
dat[which(dat$denovo.ckd.6mon == 'No'), 'denovo.ckd_overall'] <- 'No'

test <- dat[which(dat$recovery_at_3_month == 'AKI'), ]

plot(test$p02_scr_us, test$sCr3month)

change <- c('onset.NKD.3mon', 'onset.akd.3mon', 'onset.aki.3mon', 'onset.NKD.6mon', 'onset.akd.6mon', 'onset.aki.6mon')
dat[,change] %<>% lapply (function(x) factor(x, levels = c('Yes', 'No')))

dat$Recovery_overall <- dat$Recovery_at_3months_bin
dat[which(dat$Recovery_at_6months_bin == 'Yes'), 'Recovery_overall'] <- 'Yes'
dat[which(dat$Recovery_at_6months_bin == 'No'), 'Recovery_overall'] <- 'No'

dat$onset.NKD_overall <- dat$onset.NKD.3mon
dat[which(dat$onset.NKD.6mon == 'Yes'), 'onset.NKD_overall'] <- 'Yes'
dat[which(dat$onset.NKD.6mon == 'No'), 'onset.NKD_overall'] <- 'No'

dat$onset.aki_overall <- dat$onset.aki.3mon
dat[which(dat$onset.aki.6mon == 'Yes'), 'onset.aki_overall'] <- 'Yes'
dat[which(dat$onset.aki.6mon == 'No'), 'onset.aki_overall'] <- 'No'

dat$onset.akd_overall <- dat$onset.akd.3mon
dat[which(dat$onset.akd.6mon == 'Yes'), 'onset.akd_overall'] <- 'Yes'
```

```r
dat[which(dat$onset.akd.6mon == 'No'), 'onset.akd_overall'] <- 'No'

dat$CKD_cat_overall <- dat$CKD_cat_3month_prog
dat[which(dat$CKD_cat_6month_prog == 'Yes'), 'CKD_cat_overall'] <- 'Yes'
dat[which(dat$CKD_cat_6month_prog == 'No'), 'CKD_cat_overall'] <- 'No'

tab6 <- demoTab(vars = c('Recovery_at_3months_bin', 'onset.NKD.3mon', 'CKD_cat_3month_prog', 'onset.akd.3mon', 'onset.aki.3mon', 'Recovery_at_6months_bin', 'onset.NKD.6mon', 'CKD_cat_6month_prog', 'onset.akd.6mon', 'onset.aki.6mon', 'Recovery_overall', 'onset.NKD_overall', 'CKD_cat_overall', 'onset.akd_overall', 'onset.aki_overall'), group = 'phase.cat', data = dat, show.na = FALSE, rnames = c('Overall total recovery', 'New onset AKD on NKD', 'CKD progression', 'New onset CKD in AKD without AKI', 'New onset CKD in AKI', 'Overall total recovery', 'New onset AKD on NKD', 'CKD progression', 'New onset CKD in AKD without AKI', 'New onset CKD in AKI','Overall total recovery', 'New onset AKD on NKD', 'CKD progression', 'New onset CKD in AKD without AKI', 'New onset CKD in AKI'), cat.tests = 'fisher.test')

rownames(tab6)[seq(2, 45, 3)] <- rownames(tab6)[seq(1, 45, 3)]
final <- tab6[seq(2, 45, 3), ]

size <- tab6[seq(1, 45, 3), 1:3]

dol.loc <- matrix(rep(NA, 45), nrow = 15, ncol = 3)
for(i in 1:(ncol(final) - 1)){
  for(j in 1:nrow(final)){
    dol.loc[j, i] <- gregexpr(pattern ='\\$', final[j, i])[[1]][2]
  }
}

deno.size <- matrix(rep(NA, 45), nrow = 15, ncol = 3)
for(i in 1:ncol(size)){
  for(j in 1:nrow(size)){
    deno.size[j, i] <- as.numeric(str_extract(size[j, i], "(?<=\\=)[^;]*(?=\\$)"))
  }
}

for(i in 1:(ncol(final) - 1)){
  for(j in 1:nrow(final)){
    stri_sub(final[j, i], dol.loc[j, i], dol.loc[j, i] - 1) <- paste('/', deno.size[j, i], sep = '')
  }
}

tab6 <- final[, c(3, 1, 2, 4)]

dat$na.rec.3mon <- dat$na.rec.6mon <- 'FALSE'
dat[which(is.na(dat$NRecovery_at_3month)), 'na.rec.3mon'] <- 'TRUE'
dat[which(is.na(dat$NRecovery_at_6month)), 'na.rec.6mon'] <- 'TRUE'

dat[which(dat$na.rec.3mon == 'TRUE' & dat$na.rec.6mon == 'FALSE'), c("patient_id", 'NRecovery_at_3month', 'NRecovery_at_6month')]

dat[which(dat$na.rec.3mon == 'TRUE' & dat$na.rec.6mon == 'FALSE'), c("patient_id")]




###################################
dat$Recovery_at_1months_bin <- gsub(1, 0, dat$NRecovery_at_1month)
dat$Recovery_at_1months_bin <- factor(gsubVec(c(0, 2), c('No', 'Yes'), dat$Recovery_at_1months_bin), levels = c('Yes', 'No'))

dat$renal_function_at_admission
dat$eGFR1month

dat$new.ckd.1month <- dat$eGFR1month

dat[which(dat$renal_function_at_admission == 3), 'new.ckd.1month'] <- NA

dat$follow_up_7

dat$final_disposition_cat.7d <- dat$final_disposition_cat
dat[which(dat$follow_up_7 == 0), 'final_disposition_cat.7d'] <- NA

dat$follow_up_7 <- as.character(dat$follow_up_7)
demoTab(vars = 'follow_up_7', group = 'final_disposition_cat', data = dat)
demoTab(vars = 'follow_up_7', group = 'phase.cat', data = dat)


table(dat$p21_m1fu_scr)
```

```r
dat$scr1month.new <- 'Yes'
dat[which(is.na(dat$p21_m1fu_scr)), 'scr1month.new'] <- 'No'

dat$screnrol.new <- 'Yes'
dat[which(is.na(dat$p21_enrol_scr)), 'screnrol.new'] <- 'No'

table(dat$scr1month.new, dat$renal_function_at_admission)

dat$scr1month.new.aki <- dat$scr1month.new
dat[which(dat$renal_function_status7d != 'AKI'), 'scr1month.new.aki'] <- NA

dat$aki.scr.1mon <- dat$scr1month.new.aki
dat[which(dat$scr1month.new.aki == 'No'), 'aki.scr.1mon'] <- NA

dat$kidney.recovery.1mon <- NA
dat[which(!is.na(dat$p21_m1fu_scr/dat$p21_enrol_scr)), 'kidney.recovery.1mon'] <- 'No'
dat[which(dat$p21_m1fu_scr/dat$p21_enrol_scr <= 1), 'kidney.recovery.1mon'] <- 'Yes'

dat$Mortality_by_6months <- as.character(dat$Mortality_by_6months)
table(dat$Mortality_by_6months, dat$renal_function_status7d)
demoTab(vars = 'Mortality_by_6months', data = dat, group = 'renal_function_status7d')

dat$renal_function_at_admission.cat <- gsubVec(c(1, 2, 3), c('NKD', 'AKD', 'CKD'), dat$renal_function_at_admission)
demoTab(vars = 'renal_function_at_admission.cat', group = 'renal_function_status7d', data = dat)
demoTab(vars = 'renal_function_status7d', group = 'renal_function_at_admission.cat', data = dat)

epCr <- function(sex, race, age){
  s <- ifelse(sex == 1, 1, 0.742)
  r <- ifelse(race ==2, 1.21, 1)
  a <- age^(-0.203)
  epcr <- (75/(s * r* a* 186))^(-1/1.154)
  return(epcr)
}
dat$p01_gender
dat$p01_race
dat$p01_age

dat$gender <- gsubVec(c('female', 'male'), c(0, 1), dat$p01_gender)
dat$race <- gsub('african', '2', dat$p01_race)

dat$epcr <- mapply(epCr, dat$gender, dat$race, dat$p01_age)

dat$kidney.recovery.1mon <- NA
dat[which(!is.na(dat$p21_m1fu_scr/dat$epcr)), 'kidney.recovery.1mon'] <- 'No'
dat[which(dat$p21_m1fu_scr/dat$epcr <= 1), 'kidney.recovery.1mon'] <- 'Yes'


table(dat$screnrol.new, dat$proteinuria_at_enrollment)

toString(sprintf("'%s'", colnames(dat)[which(grepl('p21', colnames(dat)))]))

jj <- dat[which(complete.cases(dat[, c('p21_enrol_visit_seq', 'p21_enrol_visit_type', 'p21_enrol_rec_date', 'p21_enrol_scr', 'p21_enrol_gfr', 'proteinuria_at_enrollment')])), ]

change <- c('f7days', 'f1month', 'f3months', 'f6months')
dat[,change] %<>% lapply (function(x) as.character(x))

tab.fu <- demoTab(vars = c('f7days', 'f1month', 'f3months', 'f6months'), group = 'final_disposition_cat', data = dat, cat.tests = 'fisher.test')
#jWrite(tab.fu, 'follow_up.csv')

demoTab('Mortality_overall', group = 'renal_function_status7d', data = dat, cat.tests = 'fisher.test')

a <- glm('Mortality_overall ~ renal_function_status7d', data = dat, family = 'binomial')
summary(a)

demoTab(vars = c('Mortality_by_6months'), group = 'final_disposition_cat', data = dat, show.na = FALSE, cat.tests = 'fisher.test')

dat$AKI_stage_2_3 <- gsubVec(c(0, 1, 2, 3), c('None and one', 'None and one', 'two and three', 'two and three'), dat$AKI_staging)

demoTab(vars = c('Mortality_overall'), group = 'AKI_stage_2_3', data = dat, cat.tests = 'fisher.test')

#dat[which(is.na(dat$fluid_therapy)), 'fluid_therapy'] <- 0
dat[which(is.na(dat$fluid_therapy_POandIVat_initial_evaluation)), 'fluid_therapy_POandIVat_initial_evaluation'] <- 0

dat$fluid_therapy_POandIVat_initial_evaluation.cat <- factor(gsubVec(c(0, 1), c('No', 'Yes'), dat$fluid_therapy_POandIVat_initial_evaluation), levels = c('Yes', 'No'))
```

```r
dat$fluid_therapy_POandIV_during_admission.cat <- factor(gsubVec(c(0, 1), c('No', 'Yes'), dat$fluid_therapy_POandIV_during_admission), levels = c('Yes', 'No'))
dat$p05_ip_interventions__diuretic_therapy.cat <- factor(gsubVec(c(0, 1), c('No', 'Yes'), dat$p05_ip_interventions__diuretic_therapy), levels = c('Yes', 'No'))
dat$p05_ip_interventions__fluid_therapy_iv.cat <- factor(gsubVec(c(0, 1), c('No', 'Yes'), dat$p05_ip_interventions__fluid_therapy_iv), levels = c('Yes', 'No'))
dat$t02_fluid_therapy_oral.cat <- factor(gsubVec(c(0, 1), c('No', 'Yes'), ifelse(is.na(dat$t02_fluid_therapy_oral), 0, 1)), levels = c('Yes', 'No'))
#dat$overall_fluid <- dat$fluid_therapy + dat$fluid_therapy_POandIVat_initial_evaluation # Fluid Therapy covers PO and IV fluid mentioned at 'fluid_therapy_POandIVat_initial_evaluation



all <- demoTab(vars = c('fluid_therapy_POandIV_during_admission.cat', 'total_volume_fluid_therapy_POandIVat_initial_evaluation'), data = dat, group = 'phase.cat', con.tests = 'kruskal.test',
    cent.funs = c('', 'median'), var.funs = c('', 'quantile'))
all <- all[c(2, 4, 5), ]

all.s <- demoTab(vars = c('total_volume_fluid_therapy_POandIVat_initial_evaluation'), data = dat[which(dat$final_disposition_cat == 'Sent home'), ], group = 'phase.cat', con.tests = 'kruskal.test',
    cent.funs = c('median'), var.funs = c('quantile'))
all.s <- rbind(paste('$', as.numeric(str_extract(colnames(all.s), "(?<=\\=)[^;]*(?=\\$)")), '$', sep = ''), all.s)
all.s[1, 4] <- ''
all.a <- demoTab(vars = c('total_volume_fluid_therapy_POandIVat_initial_evaluation'), data = dat[which(dat$final_disposition_cat == 'Admitted'), ], group = 'phase.cat', con.tests = 'kruskal.test',
    cent.funs = c('median'), var.funs = c('quantile'))
all.a <- rbind(paste('$', as.numeric(str_extract(colnames(all.a), "(?<=\\=)[^;]*(?=\\$)")), '$', sep = ''), all.a)
all.a[1, 4] <- ''


all.s <- demoTab(vars = c('fluid_therapy_POandIV_during_admission.cat', 'total_volume_fluid_therapy_POandIVat_initial_evaluation'), data = dat[which(dat$final_disposition_cat == 'Sent home'), ], group = 'phase.cat', con.tests = 'kruskal.test',
    cent.funs = c('', 'median'), var.funs = c('', 'quantile'))
all.s <- all.s[c(2, 4, 5), ]
all.a <- demoTab(vars = c('fluid_therapy_POandIV_during_admission.cat', 'total_volume_fluid_therapy_POandIVat_initial_evaluation'), data = dat[which(dat$final_disposition_cat == 'Admitted'), ], group = 'phase.cat', con.tests = 'kruskal.test',
    cent.funs = c('', 'median'), var.funs = c('', 'quantile'))
all.a <- all.a[c(2, 4, 5), ]


tab.all <- rbind(all, all.s, all.a)
rownames(tab.all) <- c('Any fluid - all patients', '', '', 'Sent home', '', '', 'Admitted', '', '')
tab.all[c(4, 7), 4] <- ''

all <- demoTab(vars = c('p05_ip_interventions__fluid_therapy_iv.cat', 't02_fluid_therapy_iv_amount'), data = dat, group = 'phase.cat', con.tests = 'kruskal.test',
    cent.funs = c('', 'median'), var.funs = c('', 'quantile'))
all <- all[c(2, 4, 5), ]

all.s <- demoTab(vars = c('t02_fluid_therapy_iv_amount'), data = dat[which(dat$final_disposition_cat == 'Sent home'), ], group = 'phase.cat', con.tests = 'kruskal.test',
    cent.funs = c('median'), var.funs = c('quantile'), rnames = 'Sent home')
all.s <- rbind(paste('$', as.numeric(str_extract(colnames(all.s), "(?<=\\=)[^;]*(?=\\$)")), '$', sep = ''), all.s)
all.s[1, 4] <- ''
all.a <- demoTab(vars = c('t02_fluid_therapy_iv_amount'), data = dat[which(dat$final_disposition_cat == 'Admitted'), ], group = 'phase.cat', con.tests = 'kruskal.test',
    cent.funs = c('median'), var.funs = c('quantile'), rnames = 'Admitted')
all.a <- rbind(paste('$', as.numeric(str_extract(colnames(all.a), "(?<=\\=)[^;]*(?=\\$)")), '$', sep = ''), all.a)
all.a[1, 4] <- ''

all.s <- demoTab(vars = c('p05_ip_interventions__fluid_therapy_iv.cat', 't02_fluid_therapy_iv_amount'), data = dat[which(dat$final_disposition_cat == 'Sent home'), ], group = 'phase.cat', con.tests = 'kruskal.test',
    cent.funs = c('', 'median'), var.funs = c('', 'quantile'))
all.s <- all.s[c(2, 4, 5), ]
all.a <- demoTab(vars = c('p05_ip_interventions__fluid_therapy_iv.cat', 't02_fluid_therapy_iv_amount'), data = dat[which(dat$final_disposition_cat == 'Admitted'), ], group = 'phase.cat', con.tests = 'kruskal.test',
    cent.funs = c('', 'median'), var.funs = c('', 'quantile'))
all.a <- all.a[c(2, 4, 5), ]


tab.iv <- rbind(all, all.s, all.a)
rownames(tab.iv) <- c('IV fluid', '', '', 'Sent home', '', '', 'Admitted', '', '')
tab.iv[c(4, 7), 4] <- ''



all <- demoTab(vars = c('t02_fluid_therapy_oral.cat', 't02_fluid_therapy_oral'), data = dat, group = 'phase.cat', con.
```

```
     tests = 'kruskal.test',
        cent.funs = c('', 'median'), var.funs = c('', 'quantile'), rnames = 'Any fluid − all patients')
all <− all[c(2, 5), ]

all.s <− demoTab(vars = c('t02_fluid_therapy_oral'), data = dat[which(dat$final_disposition_cat == 'Sent home'), ],
     group = 'phase.cat', con.tests = 'kruskal.test',
        cent.funs = c('median'), var.funs = c('quantile'), rnames = 'Sent home')
all.s <− rbind(paste('$', as.numeric(str_extract(colnames(all.s), "(?<=\\=)[^;]*(?=\\$)")), '$', sep = ''), all.s)
all.s[1, 4] <− ''
all.a <− demoTab(vars = c('t02_fluid_therapy_oral'), data = dat[which(dat$final_disposition_cat == 'Admitted'), ],
     group = 'phase.cat', con.tests = 'kruskal.test',
        cent.funs = c('median'), var.funs = c('quantile'), rnames = 'Admitted')
all.a <− rbind(paste('$', as.numeric(str_extract(colnames(all.a), "(?<=\\=)[^;]*(?=\\$)")), '$', sep = ''), all.a)
all.a[1, 4] <− ''

all.s <− demoTab(vars = c('t02_fluid_therapy_oral.cat', 't02_fluid_therapy_oral'), data = dat[which(
     dat$final_disposition_cat == 'Sent home'), ], group = 'phase.cat', con.tests = 'kruskal.test',
        cent.funs = c('', 'median'), var.funs = c('', 'quantile'))
all.s <− all.s[c(2, 5), ]
all.a <− demoTab(vars = c('t02_fluid_therapy_oral.cat', 't02_fluid_therapy_oral'), data = dat[which(
     dat$final_disposition_cat == 'Admitted'), ], group = 'phase.cat', con.tests = 'kruskal.test',
        cent.funs = c('', 'median'), var.funs = c('', 'quantile'))
all.a <− all.a[c(2, 5), ]


tab.po <− rbind(all, all.s, all.a)
rownames(tab.po) <− c('PO fluid', '', 'Sent home', '', 'Admitted', '')
tab.po[c(3, 5), 4] <− ''



all <− demoTab(vars = c('p05_ip_interventions__diuretic_therapy.cat'), data = dat, group = 'phase.cat', con.tests = '
     kruskal.test',
        cent.funs = c('', 'median'), var.funs = c('', 'quantile'), rnames = 'Diuretic use')
tab.d <− all[c(1, 2), ]
rownames(tab.d) <− c('Diuretic use', '')

tab5 <− rbind(tab.all, tab.iv, tab.po, tab.d)
tab5 <− cbind(tab5, 'a' = c('', '', 'volume', '', '', 'volume', '', '', 'volume', '', '', 'volume', '', '', 'volume',
     '', '', 'volume', '', 'volume', '', 'volume', '', 'volume', '', ''))

tab5 <− tab5[, c(5, 3, 1, 2, 4)]

#colnames(tab5) <− c("", "\\begin{tabular}{c}Overall\\\\ ($n = 2101$) \\end{tabular}", "\\begin{tabular}{c}Observation
     \\\\ ($n = 978$) \\end{tabular}", "\\begin{tabular}{c}Intervention\\\\ ($n = 1123$) \\end{tabular}", 'P')

colnames(tab5) <− c("", "Overall", "Observation",
                              "Intervention", 'P')

change <− c('follow_up_7', 'follow_up_1m', 'follow_up_3m', 'follow_up_6m', 'Mortality_by_7days', 'Mortality_by_1month
     ', 'Mortality_by_3months', 'Mortality_by_6months')
dat[,change] %<>% lapply (function(x) as.character(x))

tab.fu <− demoTab(c('follow_up_7', 'follow_up_1m', 'follow_up_3m', 'follow_up_6m', 'Mortality_by_7days', '
     Mortality_by_1month', 'Mortality_by_3months', 'Mortality_by_6months'),
                  group = 'phase.cat', data = dat,
                  rnames = c('follow up 7d', 'follow up 1m', 'follow up 3m', 'follow up 6m', 'Mortality by 7days', '
                       Mortality by 1month', 'Mortality by 3months', 'Mortality by 6months'))

table(dat$scr1month.new)

## Mortality at

dat$Mortality_at_7days <− dat$Mortality_by_7days
dat$Mortality_at_1month <− dat$Mortality_by_1month
dat$Mortality_at_3months <− dat$Mortality_by_3months
dat$Mortality_at_6months <− dat$Mortality_by_6months

dat[which(dat$mortality_at_discharge == 'Yes'), 'Mortality_at_7days'] <− NA
dat[which(dat$Mortality_by_7days == 1), c('Mortality_at_1month', 'Mortality_at_3months', 'Mortality_at_6months')] <−
     NA
dat[which(dat$Mortality_by_1month == 1), c('Mortality_at_3months', 'Mortality_at_6months')] <− NA
dat[which(dat$Mortality_by_3months == 1), c('Mortality_at_6months')] <− NA


tab.mor <− demoTab(c('follow_up_7', 'follow_up_1m', 'follow_up_3m', 'follow_up_6m', 'Mortality_by_7days', '
     Mortality_by_1month', 'Mortality_by_3months', 'Mortality_by_6months',
                        'mortality_at_discharge', 'Mortality_at_7days', 'Mortality_at_1month', 'Mortality_at_3months', '
                            Mortality_at_6months'),
```

```
                  group = 'phase.cat', data = dat,
                  rnames = c('follow up 7d', 'follow up 1m', 'follow up 3m', 'follow up 6m', 'Mortality by 7days', '
                       Mortality by 1month', 'Mortality by 3months', 'Mortality by 6months',
                             'mortality at discharge', 'Mortality at 7days', 'Mortality at 1month', 'Mortalityat 3
                                 months', 'Mortality at 6months'))


change <- c('denovo.ckd.3mon', 'denovo.ckd.6mon', 'denovo.ckd_overall')
dat[,change] %<>% lapply (function(x) factor(x, levels = c('Yes', 'No')))

tab6.ext <- demoTab(vars = c('denovo.ckd.3mon', 'denovo.ckd.6mon', 'denovo.ckd_overall'), group = 'phase.cat', data =
     dat, show.na = FALSE, rnames = c('De novo CKD', 'De novo CKD', 'De novo CKD'), cat.tests = 'fisher.test')

rownames(tab6.ext)[seq(2, 9, 3)] <- rownames(tab6.ext)[seq(1, 9, 3)]
final <- tab6.ext[seq(2, 9, 3), ]

size <- tab6.ext[seq(1, 9, 3), 1:3]

dol.loc <- matrix(rep(NA, 9), nrow = 15, ncol = 3)
for(i in 1:(ncol(final) - 1)){
  for(j in 1:nrow(final)){
    dol.loc[j, i] <- gregexpr(pattern ='\\$', final[j, i])[[1]][2]
  }
}

deno.size <- matrix(rep(NA, 9), nrow = 15, ncol = 3)
for(i in 1:ncol(size)){
  for(j in 1:nrow(size)){
    deno.size[j, i] <- as.numeric(str_extract(size[j, i], "(?<=\\=)[^;]*(?=\\$)"))
  }
}

for(i in 1:(ncol(final) - 1)){
  for(j in 1:nrow(final)){
    stri_sub(final[j, i], dol.loc[j, i], dol.loc[j, i] - 1) <- paste('/', deno.size[j, i], sep = '')
  }
}

tab6.ext <- final[, c(3, 1, 2, 4)]


dat$p21_enrol_scr
dat$p21_m1fu_scr

dat$recovery_1month_enrollment_scr <- ifelse(dat$p21_m1fu_scr <= dat$p21_enrol_scr, 'Yes', 'No')

dat$renal_function_status7d_scr <- dat$renal_function_status7d

dat[which(dat$scr1month.new != 'Yes'), 'renal_function_status7d_scr'] <- NA

table(dat$recovery_1month_enrollment_scr, dat$renal_function_status7d_scr)

demoTab(vars = 'recovery_1month_enrollment_scr', data = dat, group = 'renal_function_status7d_scr', show.na = FALSE)

dat$eGFR_less_than75_at_1month.ch <- as.character(dat$eGFR_less_than75_at_1month)

demoTab(vars = 'eGFR_less_than75_at_1month.ch', data = dat, group = 'renal_function_status7d_scr' , show.na = FALSE)

head(dat[, c("p21_enrol_scr", "p21_m1fu_scr", "recovery_1month_enrollment_scr")])

demoTab(vars = 'kidney.recovery.1mon', data = dat, group = 'renal_function_status7d_scr')

table(dat$dialysis_at_1month, dat$renal_function_status7d_scr, dat$eGFR_less_than75_at_1month.ch)


## KDIGO and Decline

dat$realAKI_48_7days

dat$decreaseAKI_48_7days

dat$decreaseAKI_48_7days.pure <- dat$decreaseAKI_48_7days

dat[which(dat$realAKI_48_7days == 1), "decreaseAKI_48_7days.pure"] <- 0

change <- c('realAKI_48_7days', 'decreaseAKI_48_7days.pure')
dat[,change] %<>% lapply (function(x) as.character(x))

demoTab(vars = c('realAKI_48_7days', 'decreaseAKI_48_7days.pure'), group = 'renal_function_at_admission.cat', data =
```

```
    dat)

dat$renal_function_at_admission.cat.aki.at.7d.only <- dat$renal_function_at_admission.cat
dat[which(dat$renal_function_status7d != 'AKI'), 'renal_function_at_admission.cat.aki.at.7d.only'] <- NA

demoTab(vars = c('realAKI_48_7days', 'decreaseAKI_48_7days.pure'), group = 'renal_function_at_admission.cat.aki.at.7d.
    only', data = dat)

dat$in_hospital_mortality
dat$time_in_health_care_facility_less_than_24hours

table(dat$in_hospital_mortality, dat$time_in_health_care_facility_less_than_24hours)


demoTab(vars = c('renal_function_status7d'), group = 'renal_function_at_admission.cat', data = dat)


## 11/27/19
## Identify patients who would not be classified as AKD if cut off is <60 instead of <75
dat$p21_enrol_gfr
dat$Enrol_eGFR_less_60

dat$renal_function_at_admission.cat
dat$proteinuria_at_enrollment

dat$AKD_at_admission

dat$AKD_at_adm_by_egfr_only <- NA
dat[which(dat$akd_adm == 'Yes'), 'AKD_at_adm_by_egfr_only'] <- 1
dat[which(dat$proteinuria_at_enrollment == 1), 'AKD_at_adm_by_egfr_only'] <- NA

#table(dat$AKD_at_adm_by_egfr_only, dat$eGFR_less_than75_at_enrol.new)

table(dat$AKD_at_adm_by_egfr_only, dat$Enrol_eGFR_less_60) # 7 missing

dat$Enrol_eGFR_less_60.new <- ifelse(dat$p21_enrol_gfr < 60, 1, 0)

table(dat$AKD_at_adm_by_egfr_only, dat$Enrol_eGFR_less_60.new)
#     0   1
#  1 193 789
## 193 patients will no longer be classified as AKD if the cut off was lowered

dat$renal_function_status7d_NKD_vs <- gsubVec(c('AKD', 'AKI', 'CKD'), c('Other', 'Other', 'Other'),
    dat$renal_function_status7d)

ext.tab <- demoTab(vars = 'Mortality_overall', group = 'renal_function_status7d_NKD_vs', data = dat, cat.tests = '
    fisher.test',
                   rnames = c('Mortality Overall'))


dat$renal_function_status7d_NKD_vs
dat$eGFR1month
dat$eGFR_less_than75_at_1month.ch

table(dat$renal_function_at_admission.cat, dat$eGFR_less_than75_at_1month)

table(dat$renal_function_status7d_scr)

demoTab(vars = 'eGFR_less_than75_at_1month.ch', data = dat, group = 'renal_function_status7d_scr' , show.na = FALSE)

dat$p21_m1fu_scr

dat$eGFR_less_than75_at_1month.new <- ifelse(dat$p21_m1fu_gfr < 75, 'Yes', 'No')

demoTab(vars = 'eGFR_less_than75_at_1month.new', data = dat, group = 'renal_function_status7d_scr' , show.na = FALSE)

dat[which(dat$renal_function_status7d_scr == 'NKD' & is.na(dat$eGFR_less_than75_at_1month.new)), c("p21_m1fu_gfr", "
    p21_m1fu_scr")]

###12/11/19 Additional Request

dat$onset.NKD.3mon.75 <- NA
dat[which(dat$renal_function_status7d == 'NKD' & dat$eGFR_less_than75_at_3month == 0), 'onset.NKD.3mon.75'] <- 'No'
dat[which(dat$renal_function_status7d == 'NKD' & dat$eGFR_less_than75_at_3month == 1), 'onset.NKD.3mon.75'] <- 'Yes'

dat$eGFR_less_than75_at_6month <- ifelse(dat$p21_m6fu_gfr < 75, 1, 0)

dat$onset.NKD.6mon.75 <- NA
dat[which(dat$renal_function_status7d == 'NKD' & dat$eGFR_less_than75_at_6month == 0), 'onset.NKD.6mon.75'] <- 'No'
```

```
dat[which(dat$renal_function_status7d == 'NKD' & dat$eGFR_less_than75_at_6month == 1), 'onset.NKD.6mon.75'] <- 'Yes'

table(dat$onset.NKD.3mon, dat$onset.NKD.3mon.75)

table(dat$renal_function_status_1month, dat$onset.NKD.3mon)

dat$renal_function_status_1month.chr <- factor(gsubVec(c(1, 2, 3), c('NKD', 'AKD', 'CKD'),
     dat$renal_function_status_1month), levels = c('NKD', 'AKD', 'CKD'))

table(dat$renal_function_status_1month.chr, dat$onset.NKD.3mon)

dat[which(dat$onset.NKD.3mon == 'Yes'), 'renal_function_status_1month.chr']
dat[which(dat$onset.NKD.3mon == 'Yes'), 'renal_function_status_1month']

# dat$renal_function_at_admission.cat
# dat$renal_function_status7d
# dat$renal_function_status_1month.chr
# dat$renal_function_status_3month

library(tidyr)
library(data.table)
dat$renal_function_status_3month.chr <- gsubVec(c(1, 2, 3), c('NKD', 'AKD', 'CKD'), dat$renal_function_status_3month)
dat$renal_function_status_1month.chr <- as.character(dat$renal_function_status_1month.chr)
dat[which(is.na(dat$renal_function_status_1month.chr)), ]

#dat[, .N, by=.(renal_function_at_admission.cat, renal_function_status7d, renal_function_status_1month.chr,
     renal_function_status_3month)]

library(tidyverse)

sankey1 <- dat %>% count(renal_function_at_admission.cat, renal_function_status7d)
colnames(sankey1) <- c("source", "target", "value")

sankey1$source <- gsubVec(c('NKD', 'AKD', 'CKD'), c(0, 1, 2), sankey1$source)
sankey1$target <- gsubVec(c('NKD', 'AKD', 'AKI', 'CKD'), c(3, 4, 5, 6), sankey1$target)

sankey2 <- dat %>% count(renal_function_status7d, renal_function_status_1month.chr)
colnames(sankey2) <- c("source", "target", "value")
sankey2[is.na(sankey2)] = "NA"

sankey2$source <- gsubVec(c('NKD', 'AKD', 'AKI', 'CKD'), c(3, 4, 5, 6), sankey2$source)
sankey2$target <- gsubVec(c('NKD', 'AKD', 'CKD', 'NA'), c(7, 8, 9, 10), sankey2$target)

sankey3 <- dat %>% count(renal_function_status_1month.chr, renal_function_status_3month.chr)
colnames(sankey3) <- c("source", "target", "value")
sankey3[is.na(sankey3)] = "NA"

sankey3$source <- gsubVec(c('NKD', 'AKD', 'CKD', 'NA'), c(7, 8, 9, 10), sankey3$source)
sankey3$target <- gsubVec(c('NKD', 'AKD', 'CKD', 'NA'), c(11, 12, 13, 14), sankey3$target)

sankey.tot <- data.frame(rbind(sankey1, sankey2, sankey3))

change <- c('source', 'target', 'value')
sankey.tot[,change] %<>% lapply (function(x) as.numeric(x))

sankey.tot <- arrange(sankey.tot, source, target)

nodes = data.frame("name" =
 c("NKD",
 "AKD",
 "CKD",
 "NKD",
 "AKD",
 "AKI",
 "CKD",
 "NKD",
 "AKD",
 "CKD",
 "NA",
 "NKD",
 "AKD",
 "CKD",
 "NA"))

nodes1 = data.frame("name" =
 c("NKD",
 "AKD",
 "CKD",
 "NKD",
```

```r
 "AKD",
 "AKI",
 "CKD"))

library(networkD3)

require(RColorBrewer)

match(sankey.tot$source, nodes$name)

f <- function(pal) brewer.pal(brewer.pal.info[pal, "maxcolors"], pal)
cols <- f("Set1")

nodes[which(nodes$name == "NKD"), 'color'] <- '#FFFFFF'
nodes[which(nodes$name == "AKD"), 'color'] <- '33CCCC'  #'#87CEEB' skyblue
nodes[which(nodes$name == "CKD"), 'color'] <- '#00008B'
nodes[which(nodes$name == "AKI"), 'color'] <- 'C55A11'
nodes[which(nodes$name == "NA"), 'color'] <- '#808080'
nodes[which(nodes$name == "NA"), 'color'] <- '#000000'

sankey.1 <- as.data.frame(sankey1)

sankeyNetwork(Links = sankey.1, Nodes = nodes1,
 Source = "source", Target = "target",
 Value = "value", NodeID = "name")

library(plotly)

p <- plot_ly(
    type = "sankey",
    orientation = "h",

    node = list(
      label = nodes$name,
      color = nodes$color,
      pad = 15,
      thickness = 20,
      line = list(
        color = "black",
        width = 0.5
      )
    ),

    link = list(
      source = sankey.tot$source,
      target = sankey.tot$target,
      value =  sankey.tot$value
    )
  ) %>%
  layout(
    font = list(
      size = 10
    )
)
print(p)

if (!require("processx")) install.packages("processx")

orca(p, "sankey.pdf")



#mytable <- xtabs( ~ Enrol_eGFR_less_60.new + eGFR_less_than75_at_enrol.new + renal_function_at_admission.cat, data = dat)


library(gmodels)


#CrossTable(dat$Enrol_eGFR_less_60.new, dat$eGFR_less_than75_at_enrol.new, dat$renal_function_at_admission.cat)


#devtools::install_github("erblast/easyalluvial")



#library(magrittr)
#change <- c('renal_function_at_admission.cat', 'renal_function_status7d', 'renal_function_status_1month.chr', 'renal_function_status_3month.chr')
```

```r
#dat[,change] %<>% lapply (function(x) as.character(x))




# library(easyalluvial)
# alluvial_wide( dplyr::select(dat, admission = renal_function_at_admission.cat, '7day' = renal_function_status7d,
#                              '1month' = renal_function_status_1month.chr, '2month' = renal_function_status_3month.chr),
#                fill_by = 'first_variable', auto_rotate_xlabs = FALSE)

library(ggalluvial)
library(tidyverse)
dat.g <- dat

dat.g[which(dat.g$sCr3month == 0), 'recovery_at_3_month'] <- NA
dat.g[which(dat.g$sCr6month == 0), 'recovery_at_6_month'] <- NA

dat.g$renal_function_status7d <- gsub('NKD', 'NKD', dat.g$renal_function_status7d)
dat.g[which(dat.g$Mortality_by_7days == 1), 'renal_function_status7d'] <- 'Mortality'
dat.g[which(is.na(dat.g$renal_function_status7d)), 'renal_function_status7d'] <- 'Loss to Follow Up'

dat.g[which(dat.g$Mortality_by_1month == 1), 'recovery_at_1_month'] <- 'Mortality'
dat.g[which(is.na(dat.g$recovery_at_1_month)), 'recovery_at_1_month'] <- 'Loss to Follow Up'

dat.g[which(dat.g$Mortality_by_3months == 1), 'recovery_at_3_month'] <- 'Mortality'
dat.g[which(is.na(dat.g$recovery_at_3_month)), 'recovery_at_3_month'] <- 'Loss to Follow Up'

dat.g[which(dat.g$Mortality_by_6months == 1), 'recovery_at_6_month'] <- 'Mortality'
dat.g[which(is.na(dat.g$recovery_at_6_month)), 'recovery_at_6_month'] <- 'Loss to Follow Up'

dat.g[which(dat.g$recovery_at_1_month == 'New onset AKD'), 'recovery_at_1_month'] <- 'AKD'
dat.g[which(dat.g$recovery_at_1_month == 'Recovery from AKD'), 'recovery_at_1_month'] <- 'NKD'
dat.g[which(dat.g$recovery_at_1_month == 'Recovery from AKI'), 'recovery_at_1_month'] <- 'NKD'

dat.g[which(dat.g$recovery_at_3_month == 'New onset AKD'), 'recovery_at_3_month'] <- 'AKD'
dat.g[which(dat.g$recovery_at_3_month == 'Recovery from AKD'), 'recovery_at_3_month'] <- 'NKD'
dat.g[which(dat.g$recovery_at_3_month == 'Recovery from AKI'), 'recovery_at_3_month'] <- 'NKD'
dat.g[which(dat.g$recovery_at_3_month %in% c('CKD progression', 'New onset CKD', 'Stable CKD')), 'recovery_at_3_month'] <- 'CKD'
dat.g[which(dat.g$renal_function_status7d == 'AKI' & dat.g$recovery_at_3_month == 'AKI'), 'recovery_at_3_month'] <- 'CKD'

dat.g[which(dat.g$recovery_at_6_month == 'New onset AKD'), 'recovery_at_6_month'] <- 'AKD'
dat.g[which(dat.g$recovery_at_6_month == 'Recovery from AKD'), 'recovery_at_6_month'] <- 'NKD'
dat.g[which(dat.g$recovery_at_6_month == 'Recovery from AKI'), 'recovery_at_6_month'] <- 'NKD'
dat.g[which(dat.g$recovery_at_6_month %in% c('CKD progression', 'New onset CKD', 'Stable CKD')), 'recovery_at_6_month'] <- 'CKD'
dat.g[which(dat.g$renal_function_status7d == 'AKI' & dat.g$recovery_at_6_month == 'AKI'), 'recovery_at_6_month'] <- 'CKD'

dat.g$renal_function_at_admission.cat <- factor(levels = c('NKD', 'AKD', 'AKI', 'CKD', 'Loss to Follow Up', 'Mortality'), dat.g$renal_function_at_admission.cat)
dat.g$renal_function_status7d <- factor(levels = c('NKD', 'AKD', 'AKI', 'CKD', 'Mortality'), dat.g$renal_function_status7d)
dat.g$recovery_at_1_month <- factor(levels = c('NKD', 'AKD', 'AKI', 'CKD', 'Loss to Follow Up', 'Mortality'), dat.g$recovery_at_1_month)
dat.g$recovery_at_3_month <- factor(levels = c('NKD', 'AKD', 'AKI', 'CKD', 'Loss to Follow Up', 'Mortality'), dat.g$recovery_at_3_month)
dat.g$recovery_at_6_month <- factor(levels = c('NKD', 'AKD', 'AKI', 'CKD', 'Loss to Follow Up', 'Mortality'), dat.g$recovery_at_6_month)

count.dat <- dat.g %>% count(renal_function_at_admission.cat, renal_function_status7d, recovery_at_1_month, recovery_at_3_month, recovery_at_6_month)

nodes[which(nodes$name == "NKD"), 'color'] <- '#FFFFFF'
nodes[which(nodes$name == "AKD"), 'color'] <- '33CCCC'  #'#87CEEB' skyblue
nodes[which(nodes$name == "CKD"), 'color'] <- '#00008B'
nodes[which(nodes$name == "AKI"), 'color'] <- 'C55A11'
nodes[which(nodes$name == "NA"), 'color'] <- '#808080'
nodes[which(nodes$name == "NA"), 'color'] <- '#000000'

ggplot(as.data.frame(count.dat),
       aes(y = n, axis1 = renal_function_at_admission.cat, axis2 = renal_function_status7d,
           axis3 = recovery_at_1_month, axis4 = recovery_at_3_month, axis5 = recovery_at_6_month)) +
  geom_alluvium(aes(fill = renal_function_at_admission.cat)) +
  geom_stratum() +
  geom_text(stat = "stratum", infer.label = TRUE) +
  scale_fill_manual(values = c("#FFFFFF", "#33CCCC", '#C55A11', "#00008B", '#808080', '#000000'))
```

```
dRG.long <- to_lodes_form(count.dat,
                          axes = 1:5,
                           id = "Cohort")

p <- ggplot(dRG.long,
       aes(x = x, stratum = stratum, alluvium = Cohort,
           y = n,
           fill = stratum, label = stratum)) +
  scale_x_discrete(expand = c(.1, .1)) +
  geom_flow(color = 'darkgray') +
  geom_stratum(alpha = 0.85) +
  geom_text(stat = "stratum", size = 3) +
  #geom_text(aes(x = stratum), dRG.long, col = 'white') +
  theme(legend.position = "none") +
  scale_fill_manual(values = c("#FFFFFF", "#33CCCC", '#C55A11', "#00008B", '#808080', '#000000'))


pdf('alluvial.pdf', height = 20, width = 30)
print(p)
dev.off()


raw.tab <- demoTab(vars = c('renal_function_at_admission.cat', 'renal_function_status7d', 'recovery_at_1_month', '
    recovery_at_3_month', 'recovery_at_6_month'), data = dat, group = 'phase.cat')

rownames(raw.tab) <- gsub('\\_', ' ', rownames(raw.tab))


dat[which(dat.g$recovery_at_6_month == 'NKD' & dat.g$recovery_at_3_month == 'CKD'), c("patient_id", "
    recovery_at_3_month", "recovery_at_6_month", "sCr3month", "sCr6month")]

dat[which(is.na(dat$recovery_at_3_month) & dat$sCr3month == 1), c("patient_id", "recovery_at_3_month", "sCr3month", '
    p21_m3fu_scr', "renal_function_status7d2", "p02_scr_us", "Mortality_by_3months", "p21_m3fu_gfr", "
    p21_m1fu_gfr_original")]

dat[which(is.na(dat$recovery_at_6_month) & dat$sCr6month == 1), c("patient_id", "recovery_at_6_month", "sCr6month", '
    p21_m6fu_scr', "renal_function_status7d2", "p02_scr_us", "Mortality_by_6months", "p21_m6fu_gfr", "
    p21_m1fu_gfr_original")]


pdf('box_plot.pdf', height = 6, width = 9)
p <- ggplot(dat[complete.cases(dat$recovery_at_3_month), ], aes(x = recovery_at_3_month, y = p21_m3fu_scr)) +
  geom_boxplot() + geom_jitter(shape=3, position=position_jitter(0.2))
p

p <- ggplot(dat[complete.cases(dat$recovery_at_6_month), ], aes(x = recovery_at_6_month, y = p21_m6fu_scr)) +
  geom_boxplot() + geom_jitter(shape=3, position=position_jitter(0.2))
p
dev.off()
```
