# Supplementary material for: Recognition and management of community-acquired acute kidney injury in low-resource settings in the ISN 0by25 trial: A multi-country feasibility study
Source: PLoS Med. 2021 Jan 14;18(1):e1003408. doi: 10.1371/journal.pmed.1003408 (PMC7808595; doi:10.1371/journal.pmed.1003408)
Supplement: S1 STOP Protocol — (DOCX) [file pmed.1003408.s007.docx]

Supporting Information

S1 ***STOP protocol***

**Protocol for Management of High Risk for AKI During the Intervention Phase**

In patients with AKI or at risk of AKI, we initiated the STOP protocol that has 4 major components, and it is described below:

1. ***S – screen for sepsis***
2. ***T – toxin / avoid toxins and drug review***
3. ***O- optimize bp/Volume Status***
4. ***P – Prevention***
5. ***S – Screen for sepsis***
   1. *Identify the source of infection and treat*

Choice of antibiotics and protocol for treatment was specific for each cluster. No recommendations were made.

1. ***T – Toxin / avoid toxins and drug review***

*2.1 Drugs requiring dose reduction or cessation*

The research coordinators recorded the most common medications that are metabolized and excreted by the kidneys and adjusted for the estimated eGFR with help from the teleconsultation physicians.

***Medication review***

Patients at risk for AKI or those who develop AKI required an adjustment of all prescribed medications. Teleconsultation physicians recommended temporary cessation of ACEi and ARBs in patients with dehydration, hypotension, and/or deteriorating renal function. Use of non-steroidal anti-inflammatory (NSAIDs) drugs was actively questioned and recommendation to withhold made. Where clinically indicated, aminoglycosides were continued to be used, paying careful attention to renal function and drug levels (if levels were available).

2.2 Patient had drug card with doses of:

- Aspirin
- Simvastatin
- ACEi – angiotensin converter enzyme inhibitor - Captopril/ Enalapril/ Lisinopril / Ramipril
- ARBs - angiotensin receptor blocker - losartan/valsartan
- Furosemide
- Tenofovir

2.3 Suspend drug when:

- Evaluation of causal relationship indicates that the nephrotoxin is the potential cause of AKI/AKD.
- A suitable and less nephrotoxic drug is available
- Nephrotoxin is considered non-essential

2.4 Drugs that need to be suspended:

- ACEi / ARBs – patient hypotensive and has AKI
  - Except in patients with scleroderma renal crises
- Simvastatin - Statins muscular toxicity
- Furosemide – Patient fluid depleted – diuretic will worsen hypovolemia
- Tenofovir

2.5 Drug that needs to be reviewed in AKI:

- Non-Steroidal anti-inflammatory drugs
- Aminoglycoside antibiotics - Gentamicin
- Drugs that accumulate – e.g., other antibiotics/digoxin

1. ***O- Optimize bp/Volume Status***
   1. ***Fluid Status Assessment***

***Optimization of fluid status***

Fluid volume status was assessed concerning both fluid depletion and fluid overload. Patients at risk of dehydration due to poor oral intake were prescribed PO or IV fluids according to health care provider discretion. Protocol directions were provided by the teleconsultation physicians and assessed by the research coordinators. The degree to which protocol was followed, and reasons for not following were recorded.

***Optimization of blood pressure***

Hypotension systolic blood pressure (SBP) < 90 mmHg / mean arterial pressure (MAP) < 65 mmHg) in patients with signs/symptoms of hypotension were treated with IV fluids or vasopressor agents where indicated and available. In cases of relative hypotension, when there was a decrease in BP from known pre-morbid levels in the absence of symptoms of hypotension, treatment was according to teleconsultation, and health care provider discretion as maintenance of SBP according to premorbid values may play an important role in preventing kidney injury.

In patients with overt hypotension, blood pressure targeted was to a MAP of ≥ 65 mmHg. In order to achieve the goal teleconsultation, physicians recommended cessation of all drugs that could induce hypotension, such as antihypertensive drugs, diuretics, withholding drugs that interfere with renal autoregulation (ACEi / ARBs), and correction of hypovolemic. Vasopressors were not usually available, thus if those drugs were considered, transfer for a higher level of care was recommended.

1. ***P-Prevention***

Determination of AKI etiology and initiate appropriate treatment. The teleconsultation health care provider and teleconsultation physician determined the best approach for determining AKI etiology. No guidelines were provided.
