# Supplementary material for: Recognition and management of community-acquired acute kidney injury in low-resource settings in the ISN 0by25 trial: A multi-country feasibility study
Source: PLoS Med. 2021 Jan 14;18(1):e1003408. doi: 10.1371/journal.pmed.1003408 (PMC7808595; doi:10.1371/journal.pmed.1003408)
Supplement: S1 Table — Table A: Patient characteristics and risk factors by renal function status at enrollment. Table B: Patient characteristics by country. (DOCX) [file pmed.1003408.s008.docx]

**Supporting Information**

**S1 Table A** – Patient characteristics and risk factors by renal function status at enrollment.

|  | | **All** | **CKD** | **AKD** | **No kidney disease** | | **P** | |
| --- | --- | --- | --- | --- | --- | --- | --- | --- |
|  |  | **N=2101** | **N=197** | **N=1199** | **N=705** | |  | |
| **Adult** | | **1825 (87.0%)** | **621 (88.6%)** | **1031 (86.0%)** | **173 (87.8%)** | | **0.251** | |
| **Gender** | |  |  |  |  | |  | |
| **female** | | **1117 (53.2%)** | **405 (57.4%)** | **612 (51.0%)** | **100 (50.8%)** | | **0.02** | |
| **Race** | |  |  |  |  | |  | |
| **African** | | **813 (38.7%)** | **336 (47.7%)** | **451 (37.6%)** | **26 (13.2%)** | | **< 0.001** | |
| **Asian** | | **888 (42.3%)** | **167 (23.7%)** | **615 (51.3%)** | **106 (53.8%)** | |  | |
| **Aymara** | | **15 (0.7%)** | **3 (0.4%)** | **8 (0.7%)** | **4 (2.0%)** | |  | |
| **Hispanic** | | **233 (11.1%)** | **122 (17.3%)** | **72 (6.0%)** | **39 (19.8%)** | |  | |
| **Middle Eastern** | | **1 (0.0%)** | **1 (0.1%)** | **0 (0.0%)** | **0 (0.0%)** | |  | |
| **Quechua** | | **149 (7.1%)** | **75 (10.7%)** | **52 (4.3%)** | **22 (11.2%)** | |  | |
| **Comorbidities** | |  |  |  |  | |  | |
| **DM** | | **256 (12.2%)** | **47 (6.7%)** | **146 (12.2%)** | **63 (32.0%)** | | **< 0.001** | |
| **Liver disease** | | **87 (4.1%)** | **11 (1.6%)** | **65 (5.4%)** | **11 (5.6%)** | | **< 0.001** | |
| **Heart disease** | | **88 (4.2%)** | **20 (2.8%)** | **39 (3.3%)** | **29 (14.7%)** | | **< 0.001** | |
| **Lung disease** | | **119 (5.7%)** | **39 (5.5%)** | **61 (5.1%)** | **19 (9.6%)** | | **0.037** | |
| **HIV** | | **373 (17.8%)** | **145 (20.6%)** | **222 (18.5%)** | **6 (3.0%)** | | **< 0.001** | |
| **Previous diagnosis of anemia** | | **255 (12.1%)** | **63 (8.9%)** | **149 (12.4%)** | **43 (21.8%)** | | **< 0.001** | |
| **Cancer** | | **36 (1.7%)** | **11 (1.6%)** | **20 (1.7%)** | **5 (2.5%)** | | **0.635** | |
| **Hypertension** | **418 (19.9%)** | | **67 (9.5%)** | **254 (21.2%)** | **97 (49.2%)** | | **< 0.001** | |
| **Signs and symptoms associated with AKI risk** | | | | | |  | |  |
| **Dehydration** | | **1533 (73.0%)** | **484 (68.7%)** | **902 (75.2%)** | **147 (74.6%)** | | **0.007** | |
| **Diarrhea** | | **600 (28.6%)** | **215 (30.5%)** | **335 (27.9%)** | **50 (25.4%)** | | **0.287** | |
| **Vomiting** | | **1050 (50.0%)** | **329 (46.7%)** | **626 (52.2%)** | **95 (48.2%)** | | **0.057** | |
| **Sweating** | | **159 (7.6%)** | **70 (9.9%)** | **78 (6.5%)** | **11 (5.6%)** | | **0.013** | |
| **Thirst** | | **396 (18.8%)** | **190 (27.0%)** | **170 (14.2%)** | **36 (18.3%)** | | **< 0.001** | |
| **Low intake** | | **1021 (48.6%)** | **294 (41.7%)** | **615 (51.3%)** | **112 (56.9%)** | | **< 0.001** | |
| **Weakness** | | **1914 (91.1%)** | **638 (90.5%)** | **1098 (91.6%)** | **178 (90.4%)** | | **0.675** | |
| **Urinary symptoms** | | **1167 (55.5%)** | **381 (54.0%)** | **661 (55.1%)** | **125 (63.5%)** | | **0.057** | |
| **Oliguria** | | **748 (35.6%)** | **258 (36.6%)** | **399 (33.3%)** | **91 (46.2%)** | | **0.002** | |
| **Polyuria** | | **96 (4.6%)** | **39 (5.5%)** | **53 (4.4%)** | **4 (2.0%)** | | **0.107** | |
| **Dysuria** | | **432 (20.6%)** | **148 (21.0%)** | **248 (20.7%)** | **36 (18.3%)** | | **0.697** | |
| **Hematuria** | | **96 (4.6%)** | **36 (5.1%)** | **50 (4.2%)** | **10 (5.1%)** | | **0.6** | |
| **Infection** | | **1516 (72.2%)** | **537 (76.2%)** | **868 (72.4%)** | **111 (56.3%)** | | **< 0.001** | |
| **Malaria** | | **108 (5.1%)** | **42 (6.0%)** | **64 (5.3%)** | **2 (1.0%)** | | **0.019** | |
| **GI Infection** | | **410 (19.5%)** | **176 (25.0%)** | **206 (17.2%)** | **28 (14.2%)** | | **< 0.001** | |
| **Hypotension** | | **459 (21.8%)** | **136 (19.3%)** | **285 (23.8%)** | **38 (19.3%)** | | **0.049** | |
| **Whole body swelling** | | **162 (7.7%)** | **31 (4.4%)** | **92 (7.7%)** | **39 (19.8%)** | | **< 0.001** | |
| **Face swelling** | | **155 (7.4%)** | **40 (5.7%)** | **100 (8.3%)** | **15 (7.6%)** | | **0.098** | |
| **Lower limb swelling** | | **449 (21.4%)** | **105 (14.9%)** | **294 (24.5%)** | **50 (25.4%)** | | **< 0.001** | |
| **Asthenia** | | **381 (18.1%)** | **167 (23.7%)** | **175 (14.6%)** | **39 (19.8%)** | | **< 0.001** | |
| **Dyspnea** | | **335 (15.9%)** | **76 (10.8%)** | **209 (17.4%)** | **50 (25.4%)** | | **< 0.001** | |
| **Weight loss** | | **428 (20.4%)** | **143 (20.3%)** | **251 (20.9%)** | **34 (17.3%)** | | **0.493** | |
| **loss of appetite** | | **1365 (65.0%)** | **464 (65.8%)** | **789 (65.8%)** | **112 (56.9%)** | | **0.043** | |
| **Pallor** | | **393 (18.7%)** | **128 (18.2%)** | **218 (18.2%)** | **47 (23.9%)** | | **0.15** | |
| **Nephrotoxic** | |  |  |  |  | |  | |
| **ACEI** | | **252 (12.0%)** | **40 (5.7%)** | **151 (12.6%)** | **61 (31.0%)** | | **< 0.001** | |
| **NSAIDS** | | **301 (14.3%)** | **112 (15.9%)** | **154 (12.8%)** | **35 (17.8%)** | | **0.066** | |
| **Tenofovir** | | **324 (15.4%)** | **125 (17.7%)** | **193 (16.1%)** | **6 (3.0%)** | | **< 0.001** | |
| **Use of other antibiotic** | | **348 (16.6%)** | **115 (16.3%)** | **216 (18.0%)** | **17 (8.6%)** | | **0.004** | |

P tests assume equal variances. Tests are adjusted for all pairwise comparisons within a row using the Bonferroni correction.

**S1 Table B** – Patient characteristics by country.

|  | | **All** | **Bolivia** | **Malawi** | **Nepal** | | **P** | |
| --- | --- | --- | --- | --- | --- | --- | --- | --- |
|  |  | **N=2101** | **N=399** | **N=813** | **N=889** | |  | |
| **Adult** | | 1825 (87.0%) | 385 (96.7%) | 703 (86.6%) | 737 (83.1%) | | < 0.001 | |
| **Gender** | |  |  |  |  | |  | |
| **female** | | 1117 (53.2%) | 223 (55.9%) | 434 (53.4%) | 460 (51.7%) | | 0.38 | |
| **Race** | |  |  |  |  | |  | |
| **African** | | 813 (38.7%) | 0 (0.0%) | 812 (99.9%) | 1 (0.1%) | | **< 0.001** | |
| **Asian** | | 888 (42.3%) | 0 (0.0%) | 0 (0.0%) | 888 (99.9%) | |  | |
| **Aymara** | | 15 (0.7%) | 15 (3.8%) | 0 (0.0%) | 0 (0.0%) | |  | |
| **Hispanic** | | 233 (11.1%) | 233 (58.7%) | 0 (0.0%) | 0 (0.0%) | |  | |
| **Middle Eastern** | | 1 (0.0%) | 0 (0.0%) | 1 (0.1%) | 0 (0.0%) | |  | |
| **Quechua** | | 149 (7.1%) | 149 (37.5%) | 0 (0.0%) | 0 (0.0%) | |  | |
| **Comorbidities** | |  |  |  |  | |  | |
| **DM** | | 256 (12.2%) | 92 (23.1%) | 55 (6.8%) | 109 (12.3%) | | < 0.001 | |
| **Liver disease** | | 87 (4.1%) | 23 (5.8%) | 15 (1.8%) | 49 (5.5%) | | < 0.001 | |
| **Heart disease** | | 88 (4.2%) | 35 (8.8%) | 25 (3.1%) | 28 (3.1%) | | < 0.001 | |
| **Lung disease** | | 119 (5.7%) | 34 (8.5%) | 48 (5.9%) | 37 (4.2%) | | 0.008 | |
| **HIV** | | 373 (17.8%) | 1 (0.3%) | 365 (44.9%) | 7 (0.8%) | | < 0.001 | |
| **Previous diagnosis of anemia** | | 255 (12.1%) | 22 (5.5%) | 118 (14.5%) | 115 (12.9%) | | < 0.001 | |
| **Cancer** | | 36 (1.7%) | 18 (4.5%) | 15 (1.8%) | 3 (0.3%) | | < 0.001 | |
| **Hypertension** | 418 (19.9%) | | 89 (22.3%) | 130 (16.0%) | 199 (22.4%) | | 0.002 | |
|  | | | | | |  | |  |

P tests assume equal variances. Tests are adjusted for all pairwise comparisons within a row using the Bonferroni correction.
