# Supplementary material for: Recognition and management of community-acquired acute kidney injury in low-resource settings in the ISN 0by25 trial: A multi-country feasibility study
Source: PLoS Med. 2021 Jan 14;18(1):e1003408. doi: 10.1371/journal.pmed.1003408 (PMC7808595; doi:10.1371/journal.pmed.1003408)
Supplement: S2 Table — (DOCX) [file pmed.1003408.s009.docx]

**S2 Table –** Screening and enrolling by center: Number of patients seen in each health care center, and proportion of screened and enrolled and AKD during observation and intervention phase.

|  | **# patients seen in HCC** | **screened** | % screened | **enrolled** | % enrolled | |
| --- | --- | --- | --- | --- | --- | --- |
| **Observation Phase** | | | | | |  |
| **BV** | | | | | |  |
| **Hospital Obrero 2** | **4638** | **138** | 3.02% | **90** | 64% | |
| **Quillacollo Health** | **1,030** | **101** | 9.81% | **22** | 22% | |
| **Sacaba Health** | **873** | **57** | 6.53% | **47** | 82% | |
| **PUNATA Healt care center** | **340** | **11** | 3.24% | **3** | 27% | |
| **MW** | | | | | |  |
| **Bangwe Health** | **33,398** | **242** | 0.72% | **98** | 40% | |
| **Chikwawa Distric** | **12,683** | **299** | 2.36% | **83** | 28% | |
| **Chileka Health** | **28,330** | **236** | 0.83% | **53** | 22% | |
| **Gateway Health** | **26,551** | **193** | 0.73% | **122** | 63% | |
| **Queen Elizabeth** | **18,020** | **73** | 0.41% | **33** | 45% | |
| **NP** | | | | | |  |
| **Amda Hospital** | **1,720** | **102** | 5.93% | **98** | 96% | |
| **B.P.Koirala Institut** | **12,416** | **454** | 3.66% | **326** | 72% | |
| **Dankuta Health** | **1,630** | **21** | 1.29% | **3** | 14% | |
| **Intervention Phase** | | | | | |  |
| **BV** | | | | | |  |
| **Hospital Obrero 2** | **10,055** | **227** | 2.26% | **140** | 62% | |
| **Quilacollo Health** | **2,276** | **102** | 4.48% | **49** | 48% | |
| **Sacaba Health** | **2,260** | **42** | 1.86% | **43** | 102% | |
| **Vila Galindo Health** | **940** | **55** | 5.85% | **20** | 36% | |
| **MW** | | | | | |  |
| **Bangwe Health Care** | **44,416** | **110** | 0.25% | **59** | 54% | |
| **Chikwawa Distric Hos** | **17,808** | **148** | 0.83% | **110** | 74% | |
| **Gateway Health** | **31,240** | **137** | 0.44% | **113** | 82% | |
| **Queen Elizabeth** | **31,854** | **177** | 0.56% | **149** | 84% | |
| **NP** | | | | | |  |
| **Amda Hospital** | **2,669** | **127** | 4.76% | **127** | 100% | |
| **B.P.Koirala Institut** | **18,935** | **421** | 2.22% | **310** | 74% | |
| **Dankuta Health** | **3,800** | **84** | 2.21% | **25** | 30% | |
