# Supplementary material for: Recognition and management of community-acquired acute kidney injury in low-resource settings in the ISN 0by25 trial: A multi-country feasibility study
Source: PLoS Med. 2021 Jan 14;18(1):e1003408. doi: 10.1371/journal.pmed.1003408 (PMC7808595; doi:10.1371/journal.pmed.1003408)
Supplement: S3 Table — (DOCX) [file pmed.1003408.s010.docx]

**Supporting Information**

**S3 Table** – Number and proportion of patients receiving fluid and diuretic therapy on enrollment day.

|  | | Overall | Observation | Intervention | P |
| --- | --- | --- | --- | --- | --- |
| **Any fluid - all patients** |  | **1588 (75.6%)** | **714 (73.0%)** | **874 (77.8%)** | **0.012** |
|  |  | *n* = 637 | *n* = 322 | *n* = 315 |  |
|  | volume | 1600 (1130 - 3000 ) | 1200 (900 - 1600 ) | 2500 (1725 - 3500 ) | <0.001 |
| Sent home |  | 598 (61.3%) | 220 (55.0%) | 378 (65.7%) |  |
|  |  | *n* = 239 | *n* = 79 | *n* = 160 |  |
|  | volume | 2000 (1200 - 3000 ) | 1200 (900 - 1600 ) | 2500 (1500 - 3037) | <0.001 |
| Admitted |  | 990 (87.9%) | 494 (85.5%) | 496 (90.5%) |  |
|  |  | *n* = 398 | *n* = 243 | *n* = 155 |  |
|  | volume | 1500 (1100 - 2500 ) | 1200 (850 - 1575 ) | 2500 (2000 - 3500 ) | <0.001 |
| **IV fluid** |  | **1377 (69.7%)** | **592 (62.2%)** | **785 (76.6%)** | **<0.001** |
|  |  | *n* = 1186 | *n* = 494 | *n* = 692 |  |
|  | volume | 1000 (1000 - 2000 ) | 1000 (500 - 1000 ) | 1500 (1000 - 2000 ) | <0.001 |
| Sent home |  | 469 (55.1%) | 140 (37.4%) | 329 (69.0%) |  |
|  |  | *n* = 449 | *n* = 138 | *n* = 311 |  |
|  | volume | 1000 (1000 - 1500 ) | 1000 (500 - 1000 ) | 1000 (1000 - 2000 ) | <0.001 |
| Admitted |  | 908 (80.6%) | 452 (78.2%) | 456 (83.2%) |  |
|  |  | *n* = 737 | *n* = 356 | *n* = 381 |  |
|  | volume | 1000 (1000 - 2000 ) | 1000 (500 - 1200 ) | 1500 (1000 - 2000 ) | <0.001 |
| **PO fluid** |  | **813 (38.7%)** | **421 (43.0%)** | **392 (34.9%)** | **<0.001** |
|  | volume | 600 (300 - 1000 ) | 350 (200 - 600 ) | 1000 (500 - 1500 ) | <0.001 |
| Sent home |  | 316 (32.4%) | 108 (27.0%) | 208 (36.2%) |  |
|  | volume | 1000 (400 - 1500 ) | 475 (200 - 1000 ) | 1000 (500 - 1962) | <0.001 |
| Admitted |  | 497 (44.1%) | 313 (54.2%) | 184 (33.6%) |  |
|  | volume | 500 (200 - 1000 ) | 350 (200 - 550 ) | 1000 (775 - 1200 ) | <0.001 |
| **Diuretic use** |  |  |  |  |  |
|  |  | 170 (8.6%) | 86 (9.0%) | 84 (8.2%) | 0.559 |

Number indicates median and in parentheses percentiles (25-75%); volume is expressed in ml. IV = intravenous; PO = per os (oral administration)

Sent home: if course following the initial encounter was discharge to home within 24 hours; admitted if their stay in health care facility was longer than 24 hours

P values based on Fisher’s exact test for categorical variables and Mann-Whitney U test for continuous variables.
